# Computer-assisted discovery of natural inhibitors for platelet-derived growth factor alpha as novel therapeutics for thyroid cancer

**DOI:** 10.3389/fphar.2024.1512864

**Published:** 2025-01-09

**Authors:** Hira Khalid, Farah Sattar, Iqra Ahmad, Valdir Ferreira de Paula Junior, Umar Nishan, Riaz Ullah, Hanna Dib, Khaled W. Omari, Mohibullah Shah

**Affiliations:** ^1^ Department of Biochemistry, Bahauddin Zakariya University, Multan, Pakistan; ^2^ Postgraduate Program in Veterinary Sciences, Faculty of Veterinary Medicine, State University of Ceara, Fortaleza, Brazil; ^3^ Department of Animal Science, Federal University of Ceara, Fortaleza, Brazil; ^4^ Hainan International Joint Research Center of Marine Advanced Photoelectric Functional Materials, College of Chemistry and Chemical Engineering, Hainan Normal University, Haikou, China; ^5^ Department of Chemistry, Kohat University of Science and Technology, Kohat, Pakistan; ^6^ Department of Pharmacognosy, College of Pharmacy, King Saud University, Riyadh, Saudi Arabia; ^7^ College of Engineering and Technology, American University of the Middle East, Egaila 54200, Kuwait

**Keywords:** anticancer, natural products, drug design, ethnopharmacology, virtual screening

## Abstract

Platelet-derived growth factor alpha (PDGFRA) plays a significant role in various malignant tumors. PDGFRA expression boosts thyroid cancer cell proliferation and metastasis. Radiorefractory thyroid cancer is poorly differentiated, very aggressive, and resistant to radioiodine therapy. Thus, novel anticancer drugs that inhibit its metastasis are urgently required. In this context, we proposed the PDGFRA inhibitors by an optimized structure-based drug design approach. We performed a virtual screening of metabolites derived from anticancer medicinal plants (Swertia chirayita, Myristica fragrans, and Datura metel) and successfully identified seven hits, namely cis-Grossamide K, Daturafoliside O, N-cis-feruloyltyramine, Maceneolignan H, Erythro-2-(4-allyl-2, 6-dimethoxyphenoxy)-1-(3, 4, 5-trimethoxyphenyl) propan-1, 3-diol, Myrifralignan C, and stigmasteryl-3-O-β-glucoside as potential PDGFRA inhibitors. Not only the top 7 hits exhibited higher docking scores in docking simulation but also optimal drug-likeness and non-toxic profiles in pharmacokinetics analysis among 119 compounds. Our top hits are non-mutagenic, can cross the blood-brain barrier, and inhibit p-glycoprotein, while the N-cis-feruloyltyramine has the potential to become a lead compound. The protein-ligand stability of the top 3 hits, namely cis-Grossamide K, Daturafoliside O, and N-cis-feruloyltyramine, and their interactions at the potential binding site of target protein were confirmed through molecular dynamic simulations. We also analyzed pharmacophoric features for stable binding in the PDGFRA active site. These drug candidates were further characterized to predict their biological activity spectra in the human body and medicinal characteristics to know their extensive behavior in laboratory testing. This study necessitates the *in-vitro* and *in-vivo* studies to confirm the potential of our hits for the discovery of novel therapeutics against the thyroid cancer.

## Introduction

Multiple variations or mutations in gene expression cause an imbalance in cellular proliferation, subsequently leading to cancerous cell development. Cancer has been identified as the most prevalent cause of mortality in developed as well as developing countries globally over the years, making it a chronic public health concern (Choudhari et al., 2020). A study reports that, by 2050, there will probably be 35 million novel cases of cancer reported yearly and 77% increase from 2022 level (Bray et al., 2024). Cancerous cells hold peculiar characteristics such as continued development signal of self-sufficiency, uncontrolled replication, gene instability, resisting programmed cell death, ongoing angiogenesis, and a variety of mutations (Madhavan et al., 2021).

Platelet-derived growth factor receptors alpha and beta (PDGFRA and PDGFRB) are membrane receptors that are involved in transduction of extra-cellular signals into cells, which is a critical process in a number of diseases, including many types of cancer, such as prostate cancer, ovarian, breast, pancreatic, and liver cancer, immune-mediated pathologies like systemic sclerosis (SSc), and viral infections (Mozzicafreddo et al., 2023). PDGFRs are glycoprotein dimer molecules comprised of an extracellular ligand-binding region separated into five Ig-like domains that connect to an intracellular receptor tyrosine kinase (RTK) domain via a single transmembrane alpha helix. PDGFRA binds to its corresponding ligand, platelet-derived growth factor (PDGF), and subsequently activates downstream signaling pathways to regulate cell proliferation, migration, and angiogenesis. However, these signaling pathways can also cause endothelial cell adhesion, collagen synthesis, and myofibroblast proliferation and chemotaxis. They are increased in pathological situations, which are also associated with viral infections, in patients suffering from SSc or other progressive fibrotic diseases (Alvarez et al., 2006). Overexpression of PDGFRA is strongly linked to radioiodine resistance and distant metastasis in human thyroid cancer, particularly radiorefractory thyroid cancer (RAIR-TC). In all cell and animal models, overexpression and abnormal activation of PDGFRA lowered thyroglobulin protein (TG) and sodium-iodide symporter (NIS) expression levels via altering thyroid transcription factor 1 (TTF1) transcriptional activity and nuclear translocation. Moreover, the findings show that when PDGFRA was suppressed, radioiodine absorption was restored, although migration, invasion potential, and tumor burden were all reduced (Salem et al., 2020).

Because the human PDGFRA has emerged as one of the most significant therapeutic targets for the aforementioned diseases, its modulation or inhibition is an important aspect that directs the pursuit of novel ligands or newly acquired information for developing novel, potent drugs, particularly for the treatment of cancer. Recently, Sorafenib was approved as a PDGFRA inhibitor by the National Medical Product Administration, and Lenvatinib has now entered clinical trials. While Sorafenib is known to cause severe hypertension along with fatigue, diarrhea, hand-foot skin reactions, and treatment-emerging adverse effects (Hansen et al., 2017), Lenvatinib showed rare side effects like encephalopathy syndrome and takotsubo cardiomyopathy added to hypertension, weight loss, and fatigue (Hamidi et al., 2022).

Structure-based drug design (SBDD), which stimulates the development of new drugs with a potential affinity for therapeutic targets, has proven to be an invaluable resource for rapid and cost-effective lead identification. SBDD is a dynamic, iterative, and effective strategy for drug development that involves the structural evaluation of targets. It has the potential to lower the time and expense of creating novel drug-lead compounds with therapeutic applications. In the current study, we applied the bioinformatics approach to demonstrate the correlation between PDGFRA and radioiodine resistance in thyroid cancer and created a molecular docking and molecular dynamic simulation model of PDGFRA inhibitors. In this regard, we selected three herbal medicinal plants by thorough literature survey that are also reported to have anti-cancer activity, namely *Swertia chirayita* (Roxb.) H. Karst. [Gentianaceae] (Verma et al., 2024), *Datura metel* L. [Solanaceae] (Islam et al., 2023), and *Myristica fragrans* Houtt. [Myristicacea] (Prakash and Gupta, 2013). We prepared an *in-house* library of 804 phytochemicals from these anti-cancer plants and screened them against PDGFRA to predict the best binding affinities. Post-docking analysis consisting of in-depth biological and metabolic behavior of top hits within the human body, followed by pharmacokinetic and pharmacophoric analysis, molecular dynamic simulations, biological activity spectra, and medicinal characteristics to assist in the treatment of radiorefractory thyroid cancer (RAIR-TC) more safely and potentially.

## Material and methods

### Receptor identification, retrieval, and refinement

By an extensive literature survey, we identified PDGFRA protein that belongs to the family of RTKs as a potent therapeutic target against cancer. Studies confirmed the role of PDGFRA mRNA and protein expression levels increased dramatically in thyroid cancer (Ong et al., 2018).

PDGFRA X-ray crystallographic structure was retrieved from PDB (ID: 6JOL) with a resolution of 1.9Å because protein structures with resolution between 1.5 and 2.5Å are promising for docking studies. Its structure contains an active site with the co-crystallized ligand imatinib and also has complete side chains.

The downloaded PDB structure of PDGFRA was refined in MOE (Molecular Operating Environment (MOE), 2022.02 Chemical Computing Group ULC, 1010 Sherbooke St. West, Suite #910, Montreal, QC, Canada, H3A 2R7, MOE2022. v11.18.1) for docking purposes. Firstly, all water molecules and extra ligands were removed from the protein structure to optimize the PDGFRA structure and to avoid any interference during docking. These eliminations cause empty spaces in the structure, which were filled by protonation with default parameters. After incorporating hydrogen ions, the protein structure was subjected to an energy minimization process to ensure stability using the Merck molecular force field (MMFF94x) and the conjugate gradient method (Dib et al., 2024).

### Active site selection

The already reported binding site of PDGFRA was selected using the MOE site finder tool. This tool created dummy atoms at the active site residues to estimate the binding site within accessible pockets. Consequently, ligands were confined to interact only within these specified binding sites.

### Ligand database preparation

**FIGURE 1:**
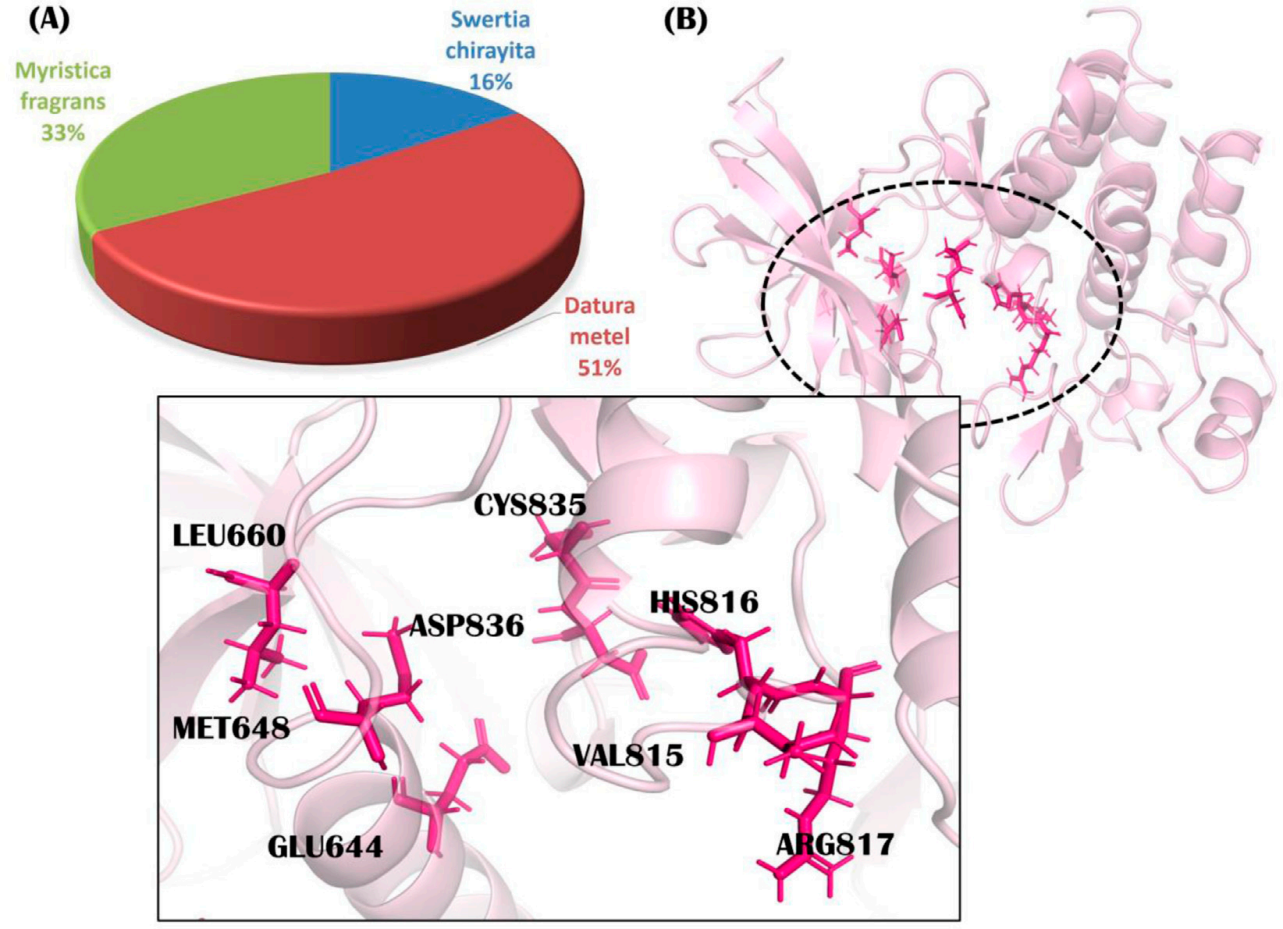
**(A)** Percentage of metabolites from each selected plant, including Swertia chirayita (16%–125 metabolites), Datura metel (51%–414 metabolites), and Myristica fragrans (33%–265 metabolites); **(B)** Important active site residues of platelet-derived growth factor receptor alpha (PDGFRA). The demonstrated active site residues are frequently involved in catalytic binding of target receptors.

We investigated the three herbal medicinal plants known to possess anticancer activity by a broad literature survey. We prepared an *in-house* set of 804 phytochemicals from *S. chirayita* (125 metabolites), *D. metel* (414 metabolites), and *M. fragrans* (265 metabolites) (Figure 1). We selected native ligand imatinib as standard for our study.

Imatinib was chosen as a standard drug due to its established efficacy as a tyrosine kinase inhibitor in the treatment of chronic myeloid leukemia (CML) and gastrointestinal stromal tumors (GISTs). Its ability to specifically inhibit PDGFRA makes it a relevant benchmark for evaluating the binding and inhibitory effects of novel compounds in our study (Joensuu et al., 2023).

The structures of standard and all ligands were downloaded from the PubCHEM database (https://pubchem.ncbi.nlm.nih.gov) or drawn in ChemDraw and saved in mdl mol format. In MOE, all structures were converted into mdb database for the docking process, followed by energy minimization (Muhammad et al., 2021).

### Molecular docking

Induced-fit docking was performed using MOE default parameters as performed previously (Shah et al., 2021; Dib et al., 2024). The results were obtained as S-scores with best-docked conformations of ligands. The S-score measures the optimized orientation, binding mode, and site of ligand and binding affinity between ligand and receptor. Five conformations of the protein-ligand complex were generated, and one with the best binding free energy and binding interactions was selected for subsequential analysis. A set of 804 phytochemicals and standard imatinib was docked against the PDGFRA receptor. The compounds showed an S-score higher than imatinib, which was selected for in-depth study.

### Docking validation

To ensure the reliability and accuracy of screening by docking protocol, we used a standard validation process using PyMOL (The PyMOL Molecular Graphics System, Version 3.0 Schrödinger, LLC). The standard imatinib docked complex, prepared in MOE, was superimposed on the original PDB complex (PDB ID: 6JOL) to derive the RMSD (root mean square deviation) value. The acceptable RMSD value is less than 2Å, and superimposition validates the docking protocol as both complexes are aligned on each other at the same binding site (Shah et al., 2024b).

### Drug-likeness analysis

The drug-likeness analysis is the way of determining the capability of a compound or metabolite to be used as a drug. The drug-likeness rules by Lipinski et al. (1997), Viswanadhan et al. (2011), Veber et al. (2002), Egan et al. (2000), Muegge et al. (2001) and bioavailability score were employed to determine the drug-likeness of our compounds. The compounds showed docking scores higher than standard imatinib and were selected for drug-likeness analysis using the SwissADME server (http://www.swissadme.ch/).

### Pharmacokinetic analysis

The pharmacokinetic analysis of top compounds was executed by ADMETlab 3.0 (https://admetlab3.scbdd.com/), a web-based program that can determine ADMET (absorption, distribution, metabolism, excretion, and toxicity) properties. ADMET outlines the effects of drugs in the human body, which include human intestinal absorption, VDss, BBB permeability, CaCo2 cell line permeability, cytochrome P450 metabolic enzyme inhibition, clearance, the AMES test, and carcinogenicity, among others.

### Generating essential pharmacophore

The Pharmacophore Query Editor in the MOE software package was used to develop an intricate pharmacophore model to investigate the molecular interactions of the top two active ligands within the PDGFRA receptor’s active site. This optimized model identified multiple essential pharmacophoric variables required for binding, such as hydrogen bond donors and acceptors, aromatic and Pi ring centers, hydrophobic atoms, and charged anionic and cationic atoms. To ensure precision, all pharmacophoric features were fine-tuned to a radius of 1.0 Å, with a tolerance of 1.2 and a minimum inclusion criterion of 50% (Ahmad et al., 2024).

### Preparation of the complex

The complexes were prepared by filling in the missing residues using SwissPDBViewer (Guex and Peitsch, 1997). Subsequently, followed by protonation adjustment using the APBS (Adaptive Poisson-Boltzmann Solver) web service (Jurrus et al., 2018). The visualization of the molecular poses of the ligand, exploring the different form configurations extracted over time, was performed using the PyMOL software (Schrödinger and DeLano, 2020).

### Molecular dynamics simulation analysis

The protein-ligand complex was subjected to study using the GROningenMAchine for Chemical Simulations (GROMACS) v2024.2 (Abraham et al., 2015). The atomistic simulations were conducted to analyze the structural changes of the complex formed by platelet-derived growth factor receptor alpha (PDGFRA) and natural inhibitors, utilizing the CHARMM36 all-atom force field based on the protocol already published (de Paula Junior et al., 2022). Subsequently, a “cubic” type box with edges of 2 nm was created for solvating the system with water molecules using TIP3P (Jorgensen et al., 1983). For the interactions studied, the following number of solvent (SOL) molecules were added: 25,374 for the PDGFRA-imatinib complex, 25,381 for the PDGFRA-daturafoliside O complex, 25,389 for the PDGFRA-N-cis-feruloyltyramine complex, and 25,379 for the PDGFRA-cis-Grossamide K complex. Additionally, ions were employed to neutralize the system. Electrostatic interactions were simulated using the fast Particle-Mesh Ewald approach, while van der Waals interactions (vdW) were calculated using the cutoff scheme. Initially, the complex underwent energy minimization using the steepest descent approach to remove bad contacts and clashes (Khan et al., 2024). The first step of equilibration was (constant number of particles, volume, and temperature) NVT ensemble using a V-rescale thermostat at 300 K, and pressure equilibration was attained using Berendsen coupling with a compressibility of 4.5 × 10^−5^ bar in NPT ensemble. Molecular dynamics (MD) simulations were conducted for a time interval of 100 ns under periodic boundary conditions. The MD results were subjected to various analyses using built-in modules of GROMACS, including the dynamic stability of the complex assessed via RMSD, the root mean square fluctuation (RMSF) of the protein residues, the radius of gyration (Rg) to determine the protein’s compactness, the number of hydrogen bond interactions between protein and ligand atoms, interaction energy (which computes both Coulombic short-range and Lennard-Jones short-range energy interactions), Gibbs free energy landscapes of the first two principal components (PC1 and PC2) evaluated using the *gmx sham* command, principal component analysis (PCA) performed with the *gmx anaeig* command using the -proj parameter, and the solvent-accessible surface area (SASA) evaluated in nm^2^, which is calculated using *gmx sasa*. These analyses were conducted to understand the protein folding dynamics and stability.

### PASS prediction

The Way2Drug informational computational platform (https://www.way2drug.com/passonline/predict.php) provides access to drug approval data in the United States and Russia, as well as computational capabilities for predicting the biological activity of drug-like organic compounds. The Prediction of Activity Spectra of Substances (PASS) algorithm examines the training set’s structure-activity relationships (SAR). It contains a considerable number of noncongeneric molecules with diverse biological activity (Poroikov et al., 2000). PASS prediction was performed on our recognized hits.

### Medicinal characteristics

The SwissADME server, which assesses the efficacy of drug candidates in biological testing, was employed to determine the medicinal qualities of the top ligands. Permitted toxicity level, chemical susceptibility, potent reaction independent of the primary target, and synthetic accessibility were determined.

## Result and discussion

Cancer, a second-leading cause of mortality, is a genetic disorder characterized by the uncontrollable multiplication of aberrant cells in the body and their dissemination to other body parts (Gomes et al., 2021). Thyroid cancer is one of the most frequent endocrine tumors, with an increasing global incidence, and RAIR-TC has a tragic survival rate due to low radioiodine uptake. PDGFRA is known to minimize the production of TG protein and NIS by interrupting the transcriptional activity and nuclear localization of TTF1, reducing the uptake of radioiodine (Yu et al., 2022). PDGFRA inhibition led to the restoration of radioiodine absorption, while invasion potential and tumor burden can be dropped.

### Potential binding site of PDGFRA

The MOE site-finding tool indicated several active sites, though only one active site previously reported in the literature was selected (Keretsu et al., 2020). The selected active site contains amino acid residues Arg597, Leu599, Lys627, Glu644, Val658, Leu660, Thr674, Cys677, Val815, His816, Arg817, Cys835, Asp836, and Phe856, among others. The important binding residues of this active site (Glu644, Met648, Leu660, Val815, His816, Arg817, Cys835, and Asp836) (Keretsu et al., 2020) are represented in Figure 1.

### Molecular docking analysis

MOE software was used for docking, and ligands were graded according to their binding affinities (S-score). The highest S-scores are related to larger negative binding energies, indicating stronger binding affinity. S-scores estimate binding energies, which correlate with Gibbs free energy. Non-covalent interactions such as hydrogen bonds (H-bonds), ionic bonds, and hydrophobic interactions are critical for the stability and selectivity of protein-ligand complexes. H-bonds have a considerable effect on drug binding affinity to target receptors.

**FIGURE 2:**
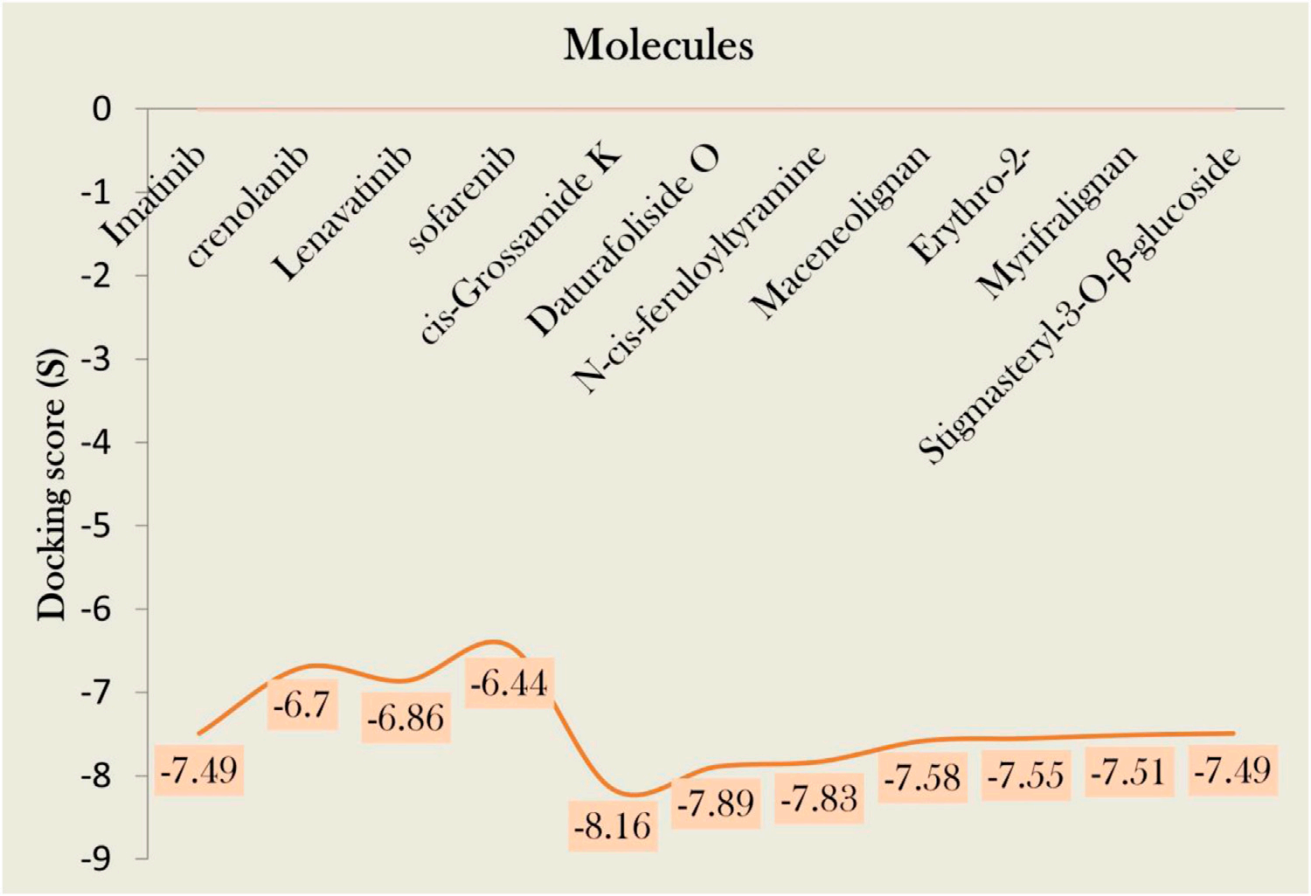
Docking score of various standards against PDGFRA and their comparisons with our hits, namely cis-Grossamide K, Daturafoliside O, and N-cis-feruloyltyramine.

In this study, we screened 804 phytochemicals from three herbal anti-cancer plants against PDGFRA. Native ligand imatinib showed a docking score of −7.49 kcal/mol (Supplementary Table S1). Our 119 compounds showed docking scores greater than standard and were subjected to drug-likeness analyses. We further compared our results with other renowned standards of PDGFRA protein, i.e., Crenolanib, Lenvatinib, and Sorafenib. These drugs represent significant advances in targeting PDGFRA mutations in various cancers. Crenolanib is a potent inhibitor of imatinib-resistant PDGFRA mutations, such as the D842V mutation, making it effective for malignancies like gastrointestinal stromal tumors with PDGFRA mutations (Heinrich et al., 2012). Lenvatinib is a multi-kinase inhibitor targeting PDGFRA, showing efficacy in improving outcomes in radioiodine-refractory thyroid cancer and hepatocellular carcinoma (Zschäbitz and Grüllich, 2018) and lastly, Sorafenib, though less effective against PDGFRA D842V mutations, can inhibit certain PDGFRA-driven tumors and is used to treat PDGFRA-mutant cancers, particularly in cases of imatinib resistance (Lierman et al., 2006). Significantly, our more than half library showed docking scores greater than Crenolanib, Lenavatinib, and Sofarenib against PDGFRA. The docking scores of these standards and their comparisons with our hits have been described in Figure 2. Out of 119, we identified 7 hits that follow the acceptable druglikeness criteria. Among 804 phytochemicals, 36 were found to be inactive against the PDGFRA receptor (Supplementary Table S1). These inactive metabolites emerged based on their absence from the MOE docking simulation output files. This absence implies that the compounds did not have substantial binding affinity for PDGFRA, as indicated by the S-scores. Focusing on compounds with higher binding affinities increases selection efficiency, prioritizes active compounds for further assessment and optimization, and ensures that resources are directed toward those with the greatest therapeutic effects.

### Lead-likeness and bioavailability

Physicochemical parameters and drug-like assets are integral drug properties that must be addressed during the drug development process (Rasgania et al., 2023). The drug-likeness rules for the top 119 compounds, along with the standard drug imatinib, which have been identified as potent PDGFRA inhibitors with relatively high S-scores, were analyzed (Supplementary Table S2). Their drug-likeness is confirmed by rules, namely Lipinski, Ghose, Veber, EGAN, MUEGGE, and bioavailability score using the SwissADME server. According to the Lipinski rule, a most authentic chemo-informatics filter, the molecular weight of a drug should be less than 500 g/mol, with no more than 5 hydrogen bond donors and 10 acceptors, a logP value of less than 5, and a TPSA value of less than 140 Å^2^ (Lipinski et al., 1997). Ghose filter sets drug-likeness requirements as measured log P is between −0.4 and 5.6, molecular weight is between 160 and 480, molar refractivity is between 40 and 130, and total atom count is between 20 and 70 (Viswanadhan et al., 2011). Veber’s rule suggests a drug can attain adequate oral bioavailability if it has 10 or fewer rotatable bonds (RTB) and TPSA of 140 Å^2^ (Veber et al., 2002; Shah et al., 2024a). The Egan filter uses multivariate statistics to develop guidelines (logP ≤5.88, TPSA <131.6 Å2) for compounds that humans well and poorly absorb (Egan et al., 2000). When assessing membrane permeability, just two descriptors (logP and TPSA) were chosen to ensure good bioavailability. It is important to consider that bioavailability filters may remove substances that enter cells via carrier-mediated transport or active absorption. Muegge’s rule modified the property ranges and provided further specifications to distinguish between drug-like and nondrug-like molecules. These are MW (200–600), LogP (-2–5), PSA ≤150, number of rings (NR)≤7, number of carbons (NC) > 4, number of heteroatoms (NH) > 1, RB ≤ 15, HBD ≤5, and HBA ≤10 (Muegge et al., 2001). After detailed analysis of 119 compounds according to the standard drug-likeness rules (Table 1), we identified 7 hits that fall in standard drug-likeness criteria. These compounds are cis-Grossamide K (compound 1), Daturafoliside O (compound 2), N-cis-feruloyltyramine (compound 3), Maceneolignan H (compound 4), Erythro-2-(4-allyl-2,6-dimethoxyphenoxy)-1-(3,4,5-trimethoxyphenyl) propan-1,3-diol (compound 5), Myrifralignan C (compound 6), and stigmasteryl-3-O-β-glucoside (compound 7).

**TABLE 1:** Hits that follow drug-likeness criteria.

Sr No.	Compound name	Lipinski rule	Ghose rule	Veber rule	Egan rule	Muegge rule	Bioavailability score
1.	*cis*-Grossamide K	Yes; 0 violation	No; 2 violations	Yes	Yes	Yes	0.55
2.	Daturafoliside O	Yes; 1 violation	No; 3 violations	Yes	Yes	Yes	0.55
3.	*N*-*cis*-feruloyltyramine	Yes; 0 violation	Yes	Yes	Yes	Yes	0.55
4.	Maceneolignan H	Yes; 0 violation	Yes	No; 1 violation	Yes	Yes	0.55
5.	Erythro-2-(4-allyl-2,6-dimethoxyphenoxy)-1-(3,4,5-trimethoxyphenyl) propan-1,3-dio*l*	Yes; 0 violation	Yes	No; 1 violation	Yes	Yes	0.55
6.	Myrifralignan C	Yes; 0 violation	Yes	Yes	Yes	Yes	0.55
7.	stigmasteryl-3-O-β-glucoside	Yes; 1 violation	No; 4 violations	Yes	Yes	No; 1 Violation	0.55

Our all hits follow the Lipinski rule and the Egan rule completely. Compounds 1, 2, and 7 showed some violations of Ghose filters. Compounds 4 and 5 have one violation of the Veber filter. Compound 7 showed only one violation of Muegge filter. The bioavailability score of 0.55 validates that all the compounds have good absorption (Oashi et al., 2011). These remarkable results confirm that our hits share structural similarities with ideal drugs. Our hits satisfactory adherence to druglikeness standards is due to their physicochemical qualities falling within an acceptable range (Table 1). This association is significant because it improves the suitability of hits for potential drug development.

### Pose validation

**FIGURE 3:**
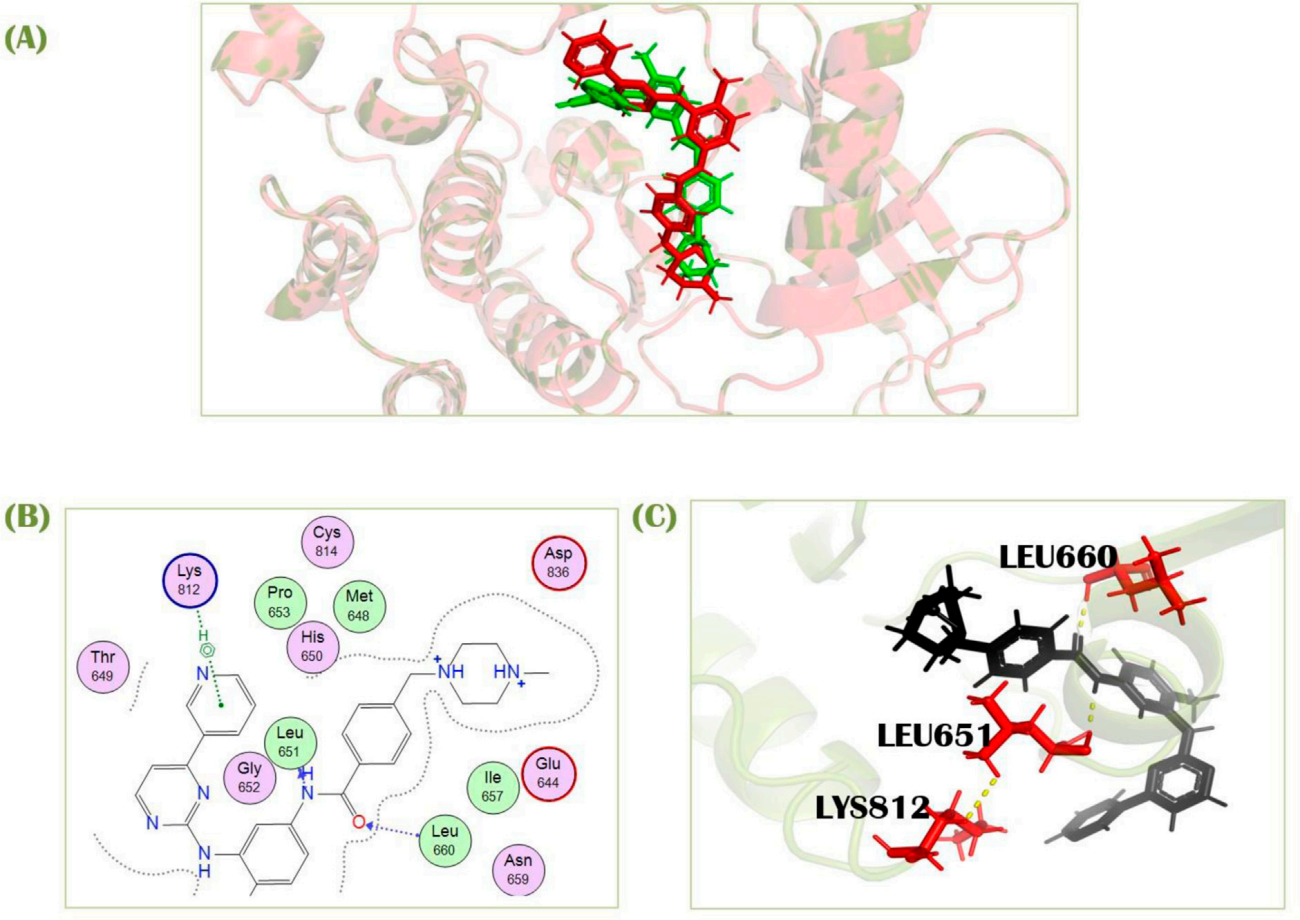
**(A)** Superimposition of standard docked complex and PDB reference complex (ID; 6JOL) **(B)** 2D **(C)** 3D interaction of imatinib with PDGFRA.

To assure the docking protocol, two complexes were prepared, one as a reference complex downloaded from pdb (ID; 6JOL), and one was our docked complex with identical co-crystallized ligand used as standard in this study. These both complexes were aligned in PyMOL. Firstly, there was a pair-wise alignment of 279 atoms from both of the complexes, but many atoms were rejected. There were 3 refinement cycles and 3 atoms rejected in the first cycle. One atom for each second and third cycle was rejected, which reduced the number of atoms of alignment to 274 for each complex. Then atoms were processed to final alignment (Figure 3A) with an executed RMSD value of 0.75Å. Our RMSD score is in the ideal range, closer to zero, indicating that our docked complex is almost perfectly aligned with the PDB reference complex. It verifies the validity of our docking approach because the docking of our other compounds in the library performed on the same active site as the docking of the standard because they co-crystallized in the same place, as indicated by the PDB ID. Our docking results show that the standard compound attaches to a comparable pocket, which supports the validity of our docking method (Figure 3A).

### Protein-ligand interaction analysis

Protein-ligand interaction analysis was performed on the top 7 hits, namely cis-Grossamide K (compound 1), Daturafoliside O (compound 2), N-cis-feruloyltyramine (compound 3), Maceneolignan H (compound 4), Erythro-2-(4-allyl-2,6-dimethoxyphenoxy)-1-(3,4,5-trimethoxyphenyl) propan-1,3-diol (compound 5), Myrifralignan C (compound 6), and stigmasteryl-3-O-β-glucoside (compound 7).

**FIGURE 4:**
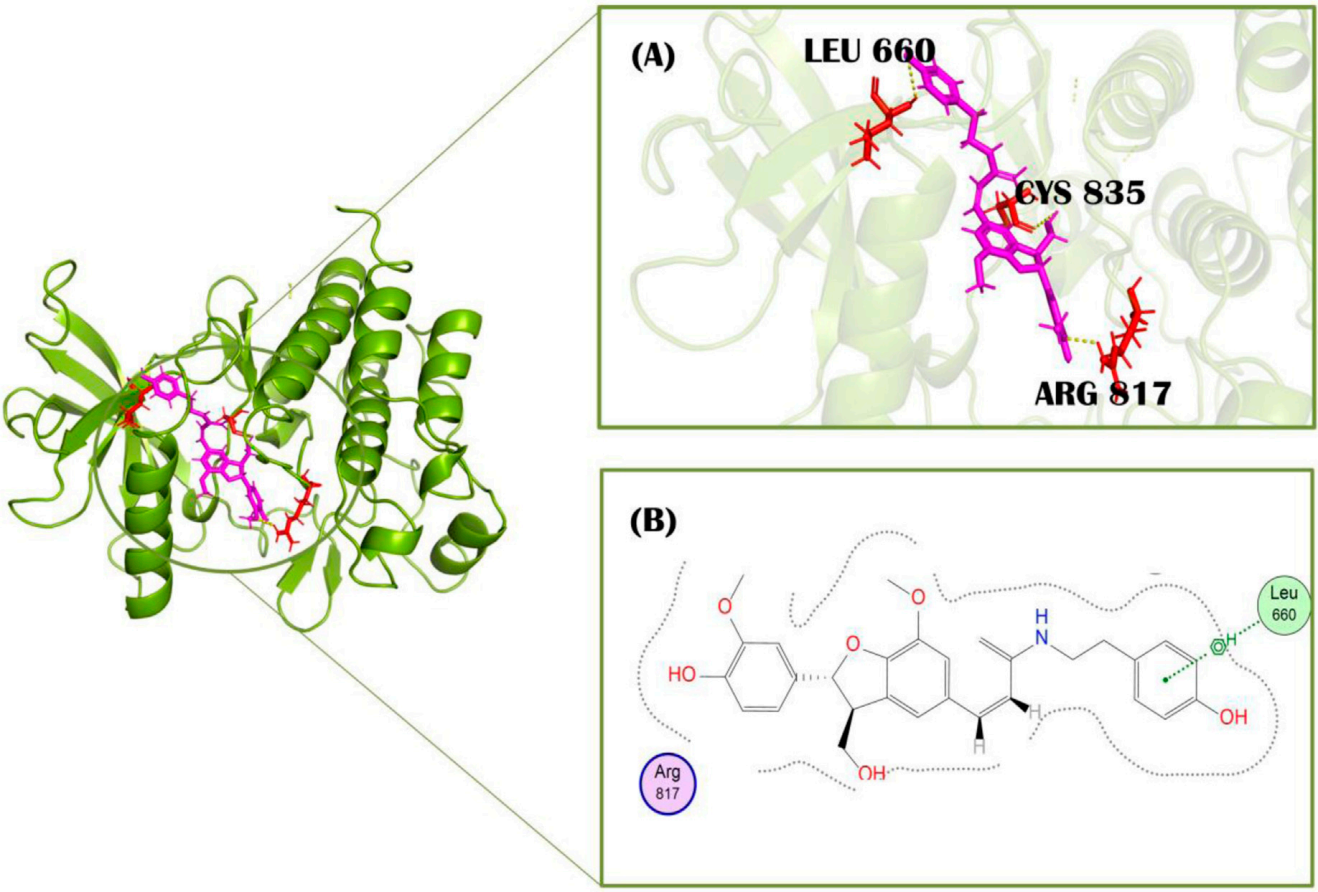
Molecular interactions of protein PDGFRA (green) with Cis-grossamide inhibitor (purple) (compound 1). **(A)** 3D image of interacting residues (red) of PDGFRA; **(B)** 2D interaction profile of Cis-grossamide.

Standard imatinib, a co-crystallized ligand of PDGFRA protein, has a docking score of −7.49 kcal/mol against this receptor. It showed two polar interactions with Leu651 and Leu660 of PDGFRA at the distances of 2.88Å for each having energies −1.9 and −3.0 kcal/mol, respectively. The standard showed one pi-H with Lys812 having binding energy −0.6 kcal/mol at the distance of 4.89 Å (Figures 3B,C). Compound 1, cis-Grossamide K, exhibited the highest S-score of −8.16 against the PDGFRA protein. The target protein showed interactions in MOE with this ligand by pi-H bond with Leu660 having energy −0.8 kcal/mol at the distance of 3.99Å. This compound showed two additional interactions with Arg817 and Cys835 in PyMol (Figure 4). That drug candidate was isolated from *D. metel*. Cis-grossamide K was reported for effectively inhibiting melanin formation and cell viability in Melan-a cells, with IC50 and LD50 values of 54.24 and 163.60 μM, respectively. The chemical group ascribed to their anti-melanogenic effect is the 3,4-disubstituted phenyl, used as a depigmenting and skin-whitening agent (Leonard et al., 2021).

Compound 2, Daturafoliside O, displayed a higher docking score of −7.89 against the target receptor isolated from *D. metel* L. [Solanaceae]. This ligand showed interactions with the target receptor by forming H-bonds (Supplementary Figure S1). It forms one interaction with His816 at the distance of 3.22 Å, having −0.9 kcal/mol energy. It also showed one H-bond with Asp836 having energy −3.1 kcal/mol at the distance of 2.83 Å. This compound showed additional interaction in Pymol with Lys627 residue. Daturafoliside O is reported for its inhibitory activity in nitric oxide production and also a good source of bioactive substances (Guo et al., 2018).

**FIGURE 5:**
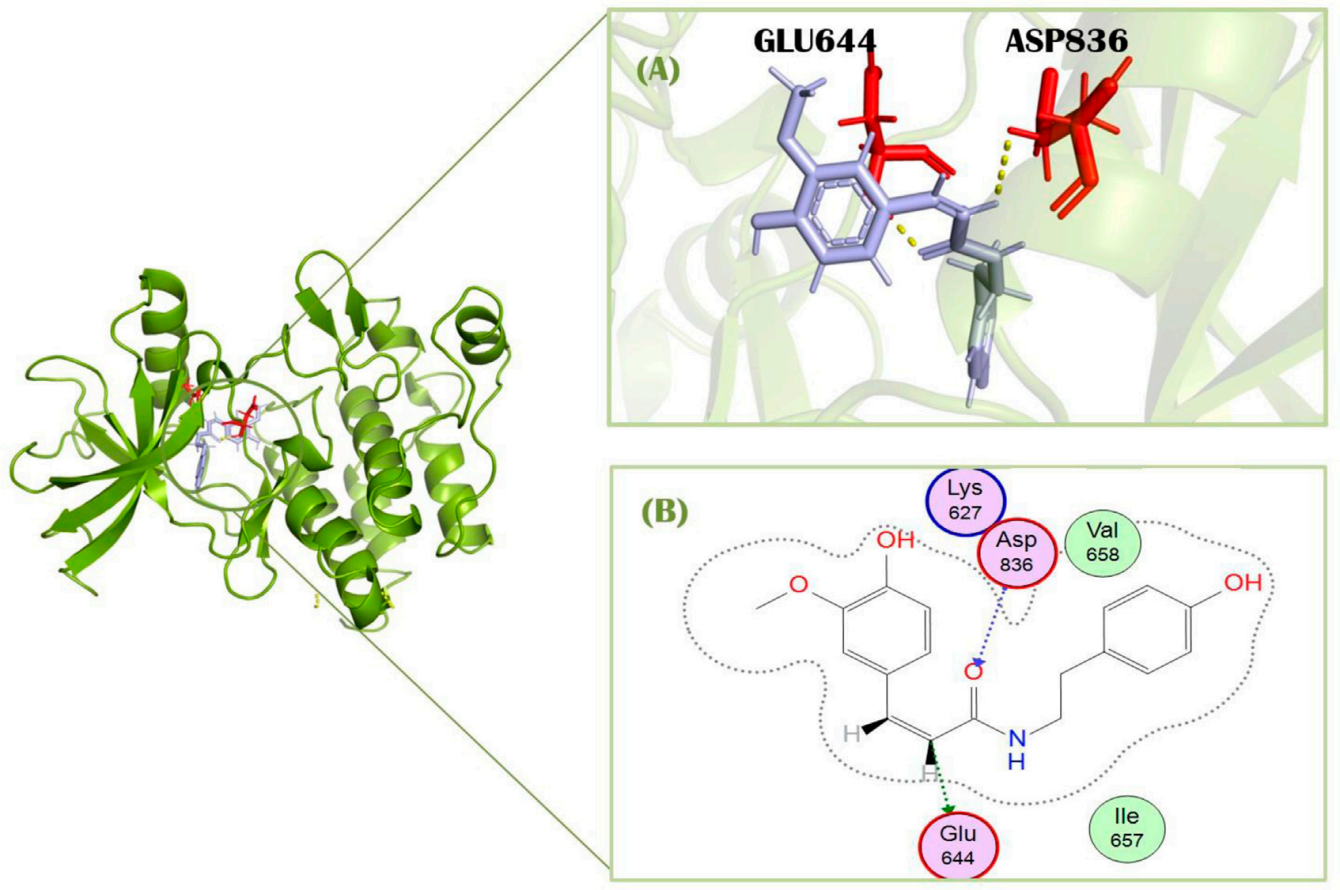
Molecular interactions of protein PDGFRA (green) with N-cis feruloyltyramine (light blue) (compound 3). **(A)** 3D image of interacting residues (red) of PDGFRA; **(B)** 2D interaction profile of N-cis feruloyltyramine.

Compound 3, N-cis-feruloyltyramine, has a docking score of −7.83 kcal/mol. This ligand was isolated from *D. metel* L. [Solanaceae] and showed interaction with the target protein by two polar bonds (Figure 5). It formed one H-bond with Glu644 at the distance of 2.99 Å with energy −1.0 kcal/mol. It also showed one significant interaction with Asp836 having 2.82 Å distance and −2.88 kcal/mol energy. N-cis-feruloyltyramine showed the highest relevance for treatment of rheumatoid arthritis as screened from pathway target compounds (Zhang et al., 2023).

Compound 4, Maceneolignan H, has a docking score of −7.58 kcal/mol against cancerous proteins. This compound was isolated from *M. fragrans* Houtt. [Myristicacea] and didn’t show any interacting residues with PDGFRA in MOE. It showed one interaction with Arrg817 in pyMOL (Supplementary Figures S2A, B). Maceneolignans are known to inhibit soluble epoxide hydrolase (sEH) (Oanh et al., 2023).

Compound 5, Erythro-2-(4-allyl-2, 6-dimethoxyphenoxy)-1-(3, 4, 5-trimethoxyphenyl) propan-1, 3-diol isolated from M. fragrans Houtt. [Myristicacea] showed docking score of −7.55 kcal/mol against target receptor. This compound showed one polar interaction with Phe856 at the distance of 2.83 Å with energy −2.5 kcal/mol (Supplementary Figures S2C, D). It showed further interaction with Arg817 in PyMOL. It is effective in antibacterial and antimicrobial treatment.

Compound 6, Myrifralignan C, exhibited a docking score of −7.51 against PDGFRA. This compound showed only one interaction in PyMOL with Arg817 (Supplementary Figures S3A, B). It was isolated from M. fragrans Houtt. [Myristicacea]. A study shows that Myrifralignan C can considerably inhibit the expression of nitric oxide synthase mRNA. This compound also possesses inflammatory activities (Cao et al., 2015).

Compound 7, stigmasteryl-3-O-β-glucoside, has a docking score of −7.49 against the PDGFRA protein. This compound showed two polar interactions with Glu644 and Met648 at the distances 3.00 Å and 3.19 Å, having energy −0.8 and −2.3 kcal/mol (Supplementary Figures S3C, D). It showed additional interaction with Asp836 of the target protein in pyMOL. It was isolated from *M. fragrans* Houtt. [Myristicacea]. Stigmasteryl-3-O-β-glucoside is reported for its antioxidant and anti-inflammatory activity (Zhanga et al., 2015).

Owing to the chemical diversity, confirmed affinity in the active site of PDGFRA, and the promising results in our drug-likeness analysis of our top 7 hits, this provides compelling evidence for proceeding with these compounds into experimental analysis to investigate their therapeutic potential further.

### Pharmacophoric features

**FIGURE 6:**
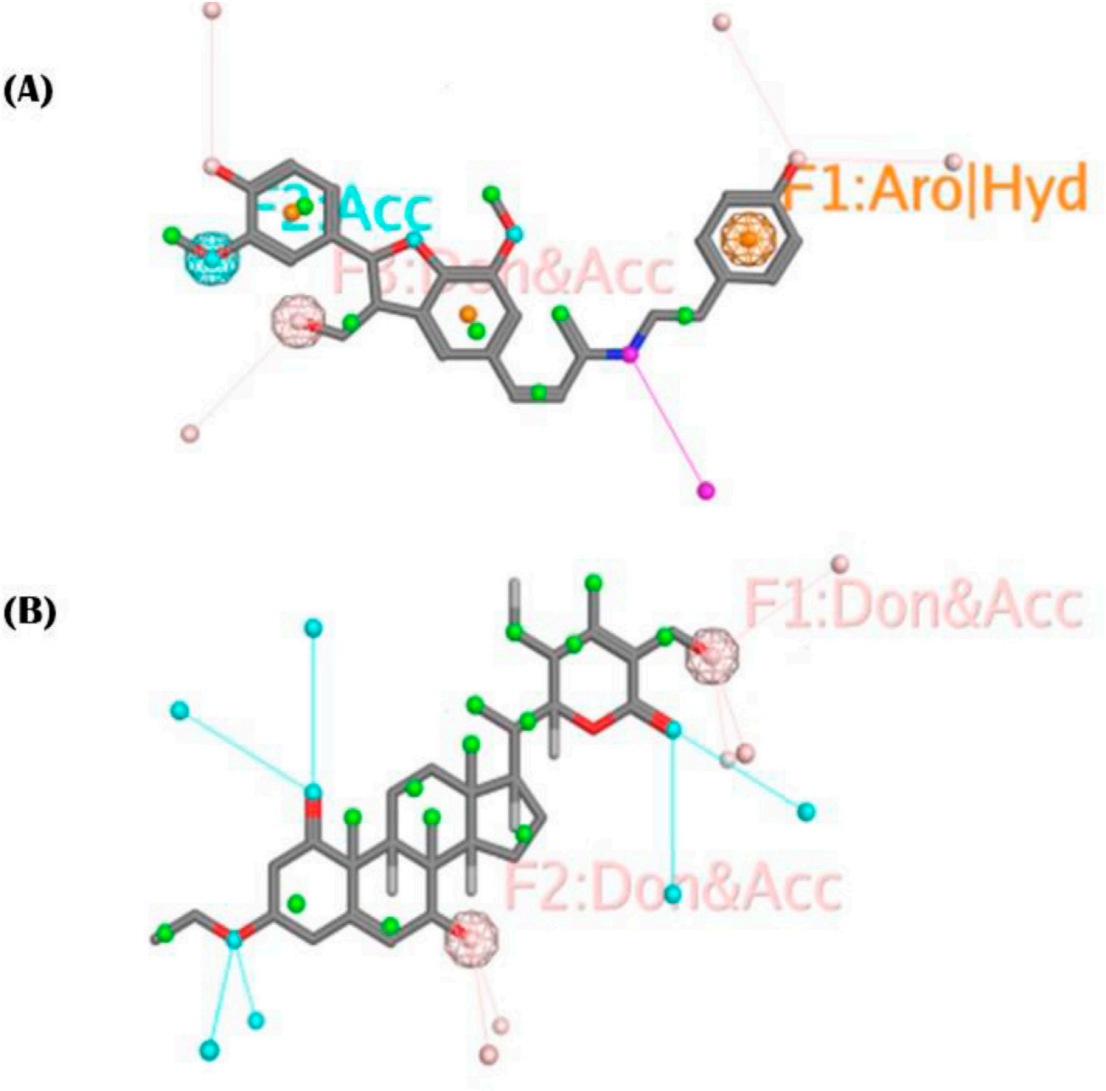
**(A)** cis-Grossamide K; **(B)** Daturafoliside O, representing molecular structure with annotated pharmacophoric features, contribute in binding to PDGFRA protein.

The objective was to selectively identify the different pharmacophoric characteristics of our hits by using a phenolic scaffold. This approach is intended to maximize their affinity and efficacy in inhibiting the PDGFRA receptor, hence improving their potential as pharmaceutical agents. To construct a pharmacophore, we introduced all the pharmacophoric features of phenolic scaffold in cis-Grossamide K and Daturafoliside O. The feature identified in cis-Grossamide K (Figure 6) is G7 labeled “Aro,” along with “hybrid,” with a radius of 0.50Å, suggesting a region in the compound that can participate in both π−π stacking and hydrophobic interactions. G17 labeled as “Don&Acc” indicates that this region is important for forming specific hydrogen bond acceptors and donors in the active site of PDGFRA. G19 in this compound is labeled as “Acc,” describing its specificity in forming hydrogen bond acceptors. These interactions can define the binding affinity, specificity, and overall biological activity of cis-Grossamide K. The features identified in Daturafoliside O (Figure 6) are G24 and G25 bonds, both labeled with “Don&Acc,” with a radius of 0.50Å for forming H-bond acceptors and donors with active site residues of PDGFRA.

The interaction profile of cis-Grossamide K reveals different bonding types, while Daturafoliside O solely shows hydrogen bonding. Despite the presence of the same functional components, such peculiar bonding can be important to these compounds’ therapeutic efficacy. This also explains the disparity in their docking scores, as cis-Grossamide K (−8.16 kcal/mol) has greater docking scores for target proteins than Daturafoliside O (−7.89 kcal/mol) (Supplementary Table S1). The specified pharmacophoric aspects are critical in the drug development process for PDGFRA inhibitors because they represent the important chemical capabilities required for compounds to interact with key residues of PDGFRA. Further investigation into these properties might help develop more potent and selective drugs by ensuring that future pharmaceuticals contain the structural components required for efficient binding.

### MD simulation analysis

**FIGURE 7:**
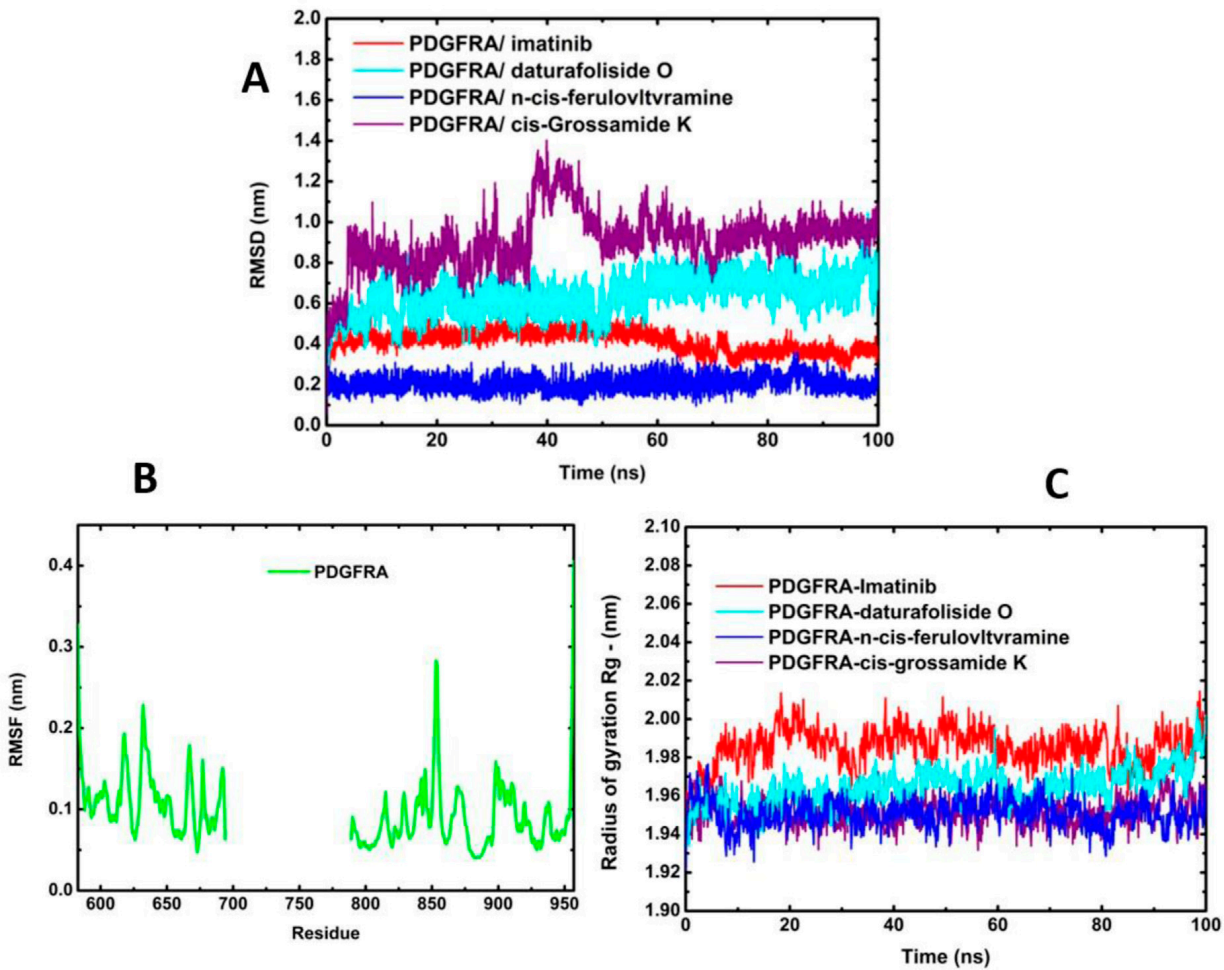
Molecular dynamics of protein 6JOL with its respective inhibitors. **(A)** Overlay of RMSD of four inhibitors; **(B)** Fluctuation analysis of the four systems overlaid in RMSF; **(C)** Analysis of radius of gyration over 100 ns.

The co-crystallized structure (6JOL-imatinib) demonstrated stability throughout the entire simulation, with the variation in the pose of imatinib ranging from 0.07 nm to 0.55 nm, and an average of 0.41 nm. This was followed by the stability of the system (6JOL-Daturafoliside O), with a variation of 1.04 nm to 0.06 nm, and an average of 0.63 nm. The complex (6JOL-N-cis-feruloyltyramine) showed the best performance in the variation of the ligand pose in the catalytic pocket of the target, with a range of 0.36 nm to 0.04 nm, and an average of 0.20 nm. However, the same performance was not observed in the system (6JOL-cis-Grossamide K), which produced a variation of 1.40 nm to 0.04 nm and an average of 0.91 nm (Figure 7A).

The RMSF graphs reveal that all systems were stable without significant fluctuation, with variation below 0.3 nm as described in the literature (Figure 7B) (Lindahl et al., 2001). The principle of Rg is defined as the ratio between the accessible surface area of a protein and that of a sphere of the same volume. This principle is fundamental for understanding the protein folding mechanism (Galzitskaya et al., 2008). As shown in Figure 7C, all systems were highly stable and compact without evidence of residue disorder.

**FIGURE 8:**
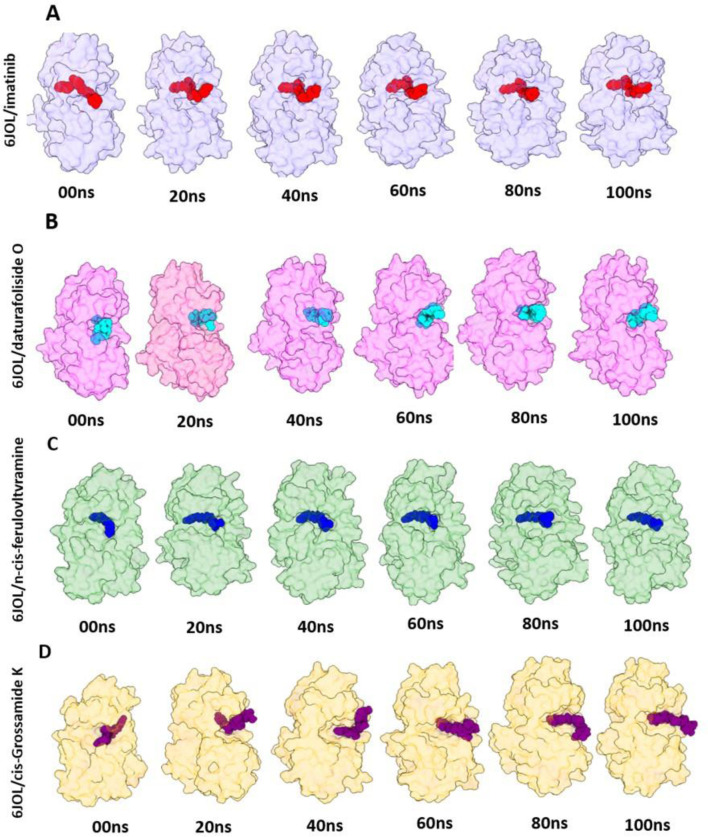
3D visualization of the molecular poses of the four inhibitors in the 6JOL target at intervals of 00 ns, 20 ns, 40 ns, 60 ns, 80 ns, and 100 ns. **(A)** Snapshots of 6JOL/imatinib; **(B)** Snapshots of 6JOL/daturafoliside O; **(C)** Snapshots of 6JOL/n-cis-feruloyltyramine; **(D)** Snapshots of 6JOL/cis-Grossamide K.

The stability of the four systems under analysis becomes evident when observing their geometric conformations. In Figure 8A–C, we observe low perturbation of the ligands imatinib, daturafoliside O, and n-cis-feruloyltyramine in the binding site of 6JOL. This analysis supports the hypothesis of high complementarity of the studied complexes, as they occupy the same catalytic pocket as the co-crystallized structure. However, in Figures 8D, despite the protein being compact and showing low variation in protein residues, the ligand cis-Grossamide K forces its way out of the catalytic pocket, causing fluctuation in the RMSD. The crystallographic structure of PDGFRA with imatinib (PDB ID 6JOL) shows a gap of 80 residues between domains 2 and 3 of the protein, this means there is a part of the protein where the resolution was not sufficient to capture these specific residues. These missing residues may be located in regions of high flexibility or areas that are difficult to resolve.

**FIGURE 9:**
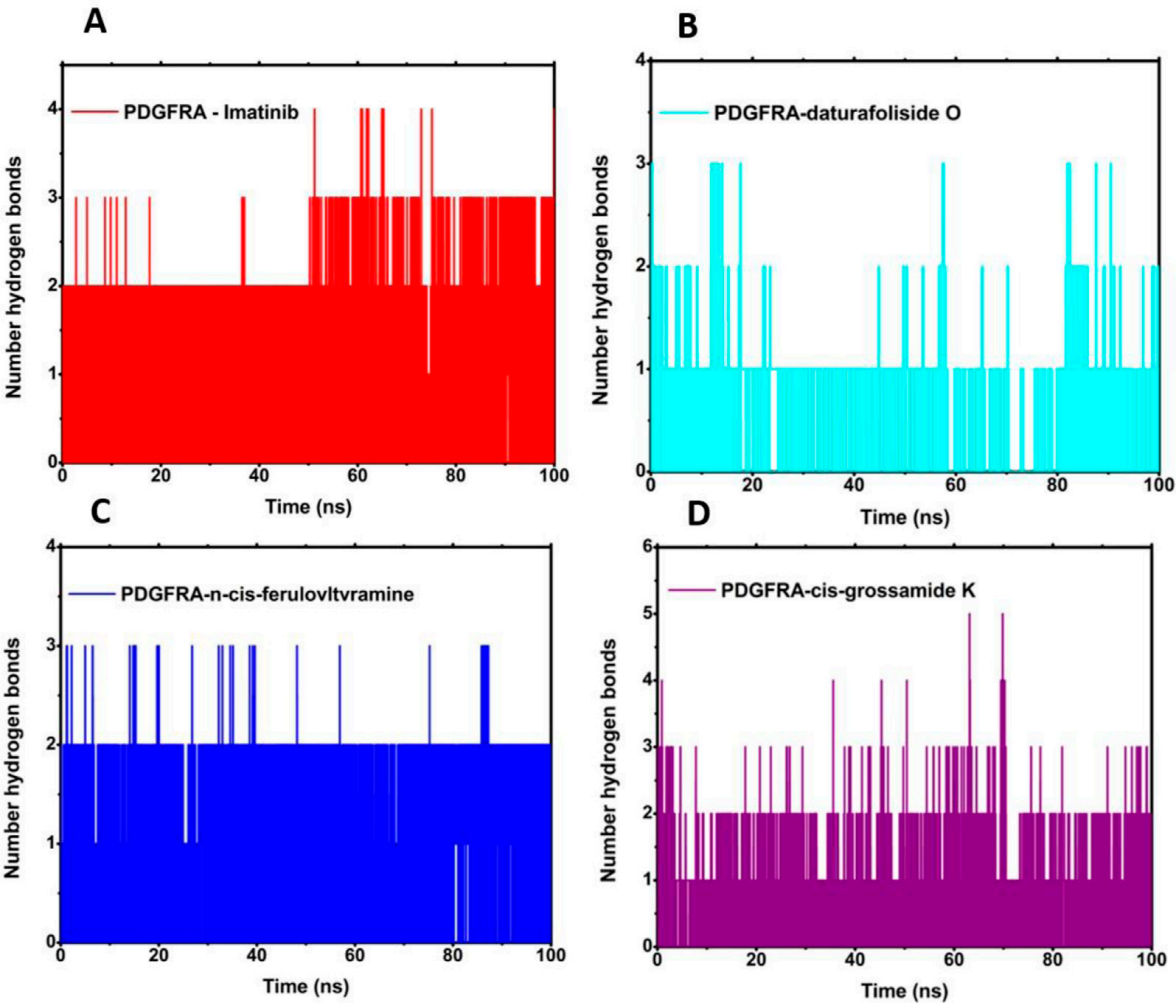
Analysis of the number of hydrogen bonds: **(A)** 6JOL/imatinib; **(B)** 6JOL/daturafoliside O; **(C)** 6JOL/n-cis-ferulovltvramine; **(D)** 6JOL/cis-Grossamide K.

The quantification of the number of hydrogen bonds revealed the existence of hydrogen interactions between the protein and the ligand throughout the entire simulation for all four evaluated systems (Figure 9). The highest participation in these interactions, with an average of 2.0, was evident for 6JOL/imatinib and 6JOL/n-cis-feruloyltyramine. The presence of these interactions suggests high complementarity of the ligand to the target, with minimal perturbation in the protein’s binding pocket (Rehman et al., 2023).

**FIGURE 10:**
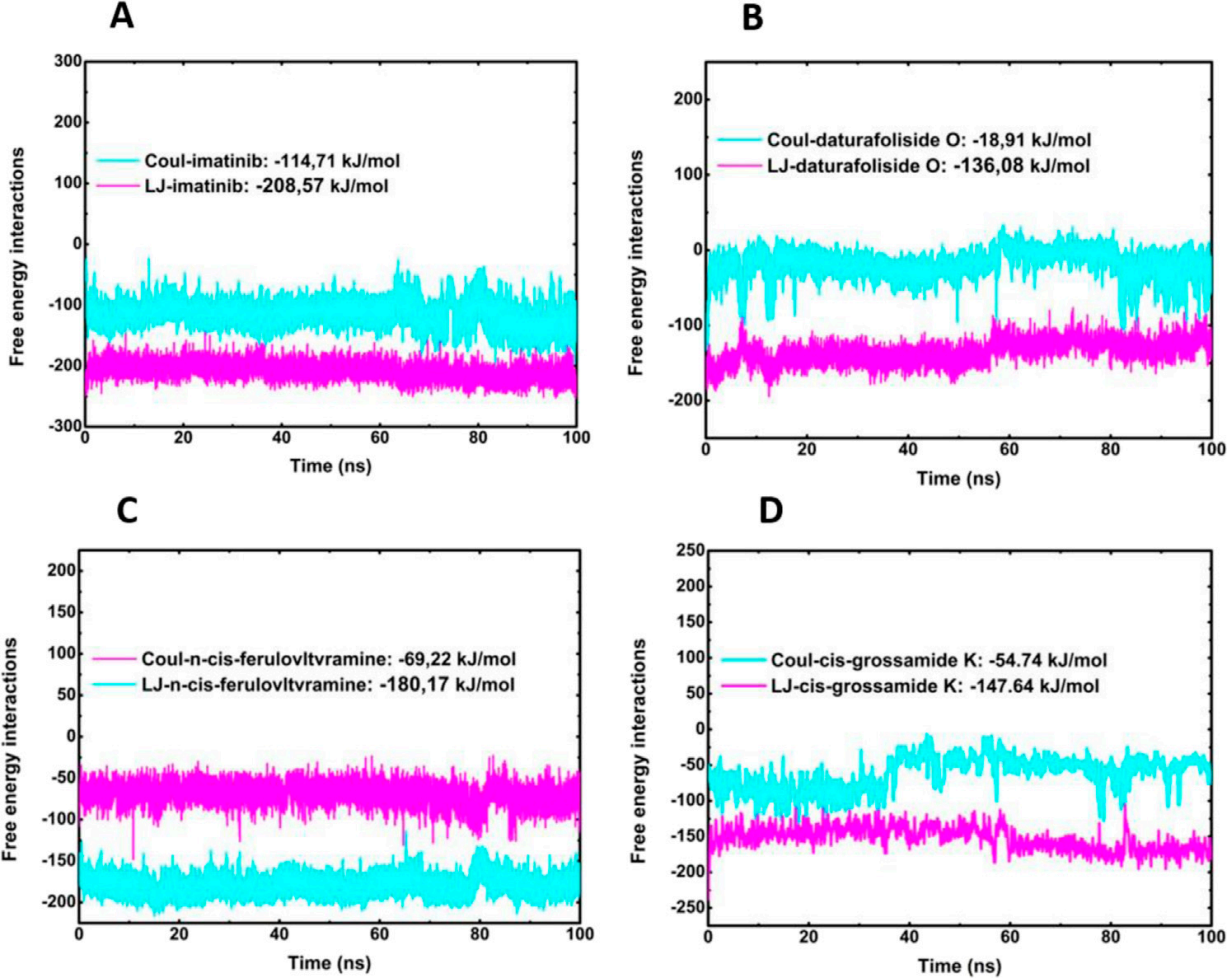
Calculation of Coul-SR and LJ-SR interaction energies of Protein-LIG. **(A)** 6JOL/imatinib; **(B)** 6JOL/daturafoliside O; **(C)** 6JOL/n-cis-ferulovltvramine; **(D)** 6JOL/cis-Grossamide K.

The protein-ligand interaction energies were confirmed by calculating the Coulomb (Coul-SR) and Lennard-Jones (LJ-SR) terms. Protein-ligand interaction energies calculated by Coulomb (Coul-SR) and Lennard-Jones (LJ-SR) terms help identify strong inhibitors. Coul-SR reflects electrostatic interactions, where more negative values indicate stronger ionic or dipole binding. LJ-SR accounts for van der Waals forces, with lower values suggesting better fit and fewer steric clashes. Strong candidates typically show highly negative total interaction energy, indicating stable and specific binding to the target site. This balance of electrostatic and van der Waals contributions highlights ligands that effectively inhibit protein activity. During the simulation, the total energy values obtained were as follows: 6JOL/imatinib produced a value of −323,28 kJ/mol, 6JOL/daturafoliside O−154,99 kJ/mol, 6JOL/n-cis-ferulovltvramine −249,39 kJ/mol, and 6JOL/cis-Grossamide K −202,38 kJ/mol. These findings suggest that the compounds daturafoliside O and n-cis-ferulovltvramine are strong candidates for inhibiting PDGFRA (Figures 10A–D).

**FIGURE 11:**
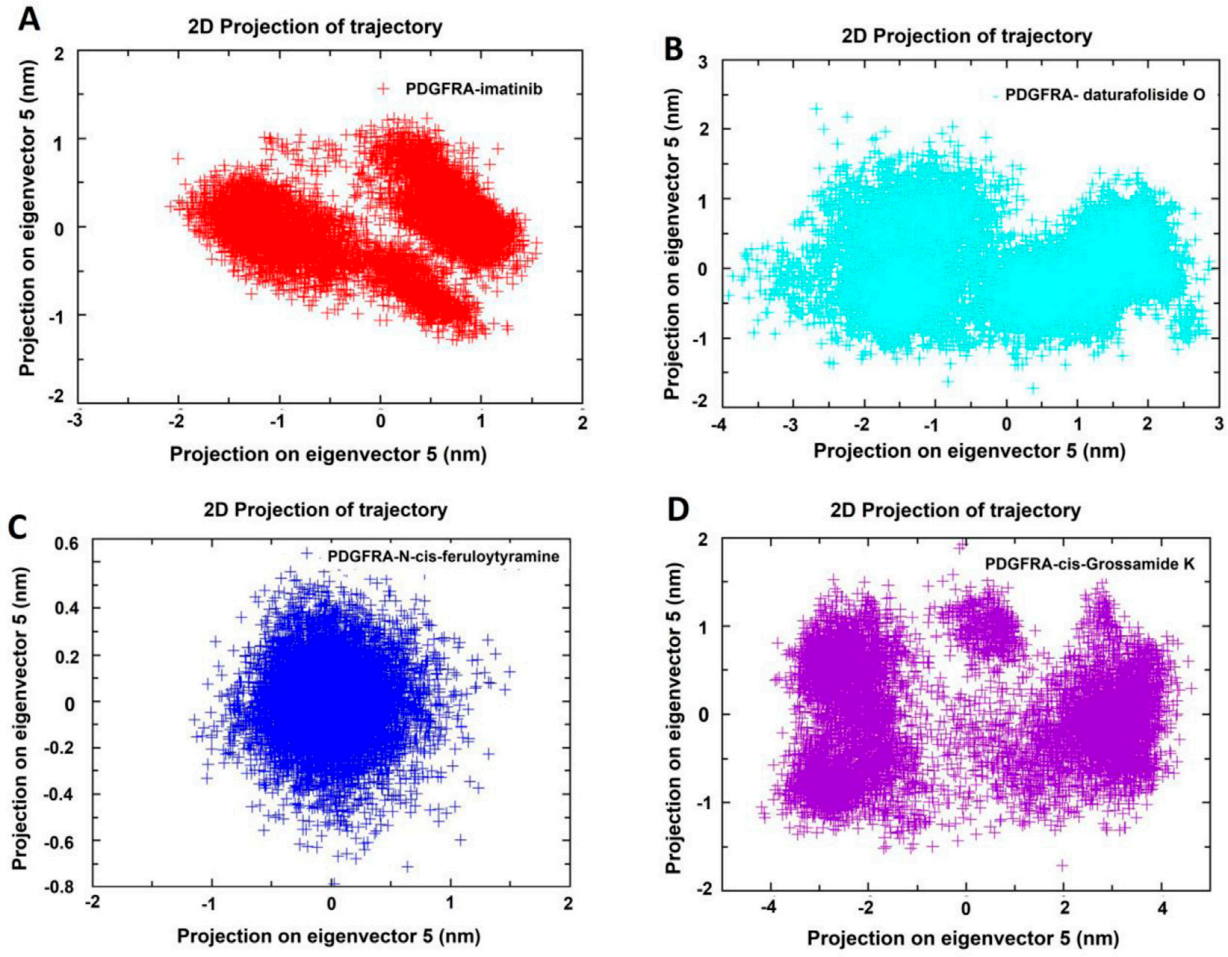
Principal Component Analysis (PCA) of PDGFRA in four systems: **(A)** PDGFRA - imatinib; **(B)** PDGFRA - daturafoliside O; **(C)** PDGFRA - N-cis-feruloyltyramine; **(D)** PDGFRA - cis-Grossamide K.

Principal Component Analysis (PCA) was performed for the four systems (PDGFRA - imatinib; PDGFRA - daturafoliside O; PDGFRA - N-cis-feruloyltyramine; PDGFRA - cis-Grossamide K) from the trajectories produced in GROMACS. The two-dimensional projections of conformational changes during the simulation were plotted in a 2D graph based on the first five eigenvectors, highlighting differences in their trajectories and conformational changes over 100 ns. In the PDGFRA - imatinib system, two regions were occupied in the PCA plot, suggesting a conformational change with overlap and sharing with the systems PDGFRA - daturafoliside O, PDGFRA - N-cis-feruloyltyramine, and PDGFRA - cis-Grossamide K (Figures 11A–D). In the 2D plot of PDGFRA - N-cis-feruloyltyramine, a more restricted conformational space was observed, suggesting a stabilizing effect of the compound with reduced structural fluctuations. However, in the PCA analysis of the PDGFRA - cis-Grossamide K system, regions outside the plane observed in PDGFRA - imatinib were noted, with a broader range of conformational states, reflecting structural fluctuations. In conclusion, the PCA analyses indicate that the inhibitors prevent the movement of the protein and conformational changes, reducing its conformational variability and internal motions, and have a different coupling mechanism compared to PDGFRA - imatinib.

**FIGURE 12:**
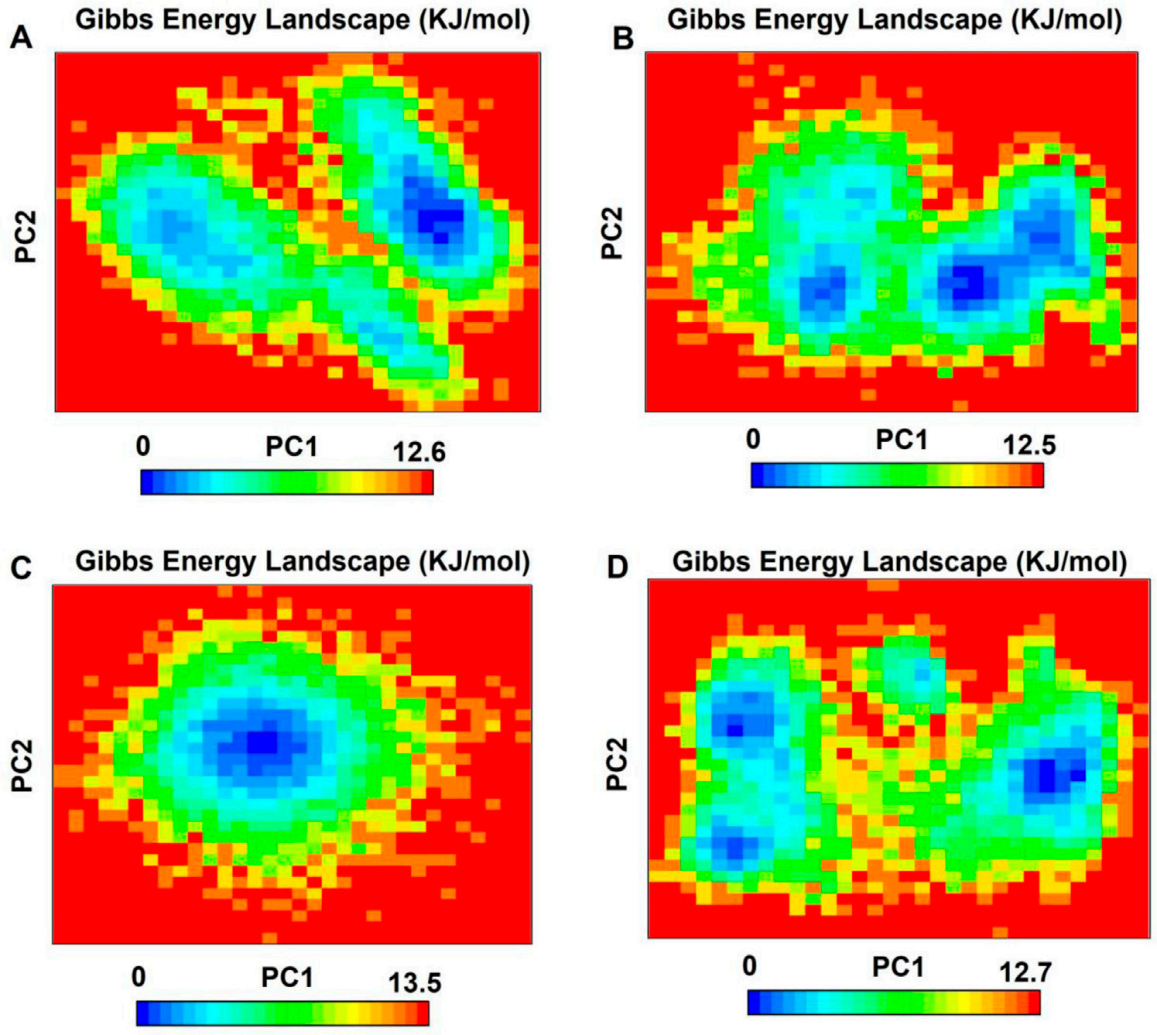
The Gibbs free energy landscape, displayed through PC1 and PC2, shows lower energy systems as the areas in deeper blue on the contour map. **(A)** PDGFRA - imatinib; **(B)** PDGFRA - daturafoliside O; **(C)** PDGFRA - N-cis-feruloyltyramine; **(D)** PDGFRA - cis-Grossamide K.

The free energy landscape reveals differences in the conformations adopted by the analyzed systems. In the co-crystalized structure, two regions with two energy minima are observed after binding with imatinib, resulting in a free energy of 12.5 kJ/mol. In the PDGFRA - daturafoliside system, two regions of lower energy are also identified, with a total free energy of 12.5 kJ/mol, located near the regions of the co-crystalized system in terms of position. In the free energy landscape of PDGFRA - N-cis-feruloyltyramine, a single region with thermodynamic stability and a free energy of 13.5 kJ/mol appears. Finally, in the PDGFRA - cis-Grossamide K complex, three energy minima are formed with distinct positions compared to the co-crystalized complex, presenting an interaction energy of 12.7 kJ/mol (Figures 12A–D). Based on these results, it can be concluded that the binding of the inhibitors to the protein blocks its activity by stabilizing specific conformations and altering the formation of energy minima.

**FIGURE 13:**
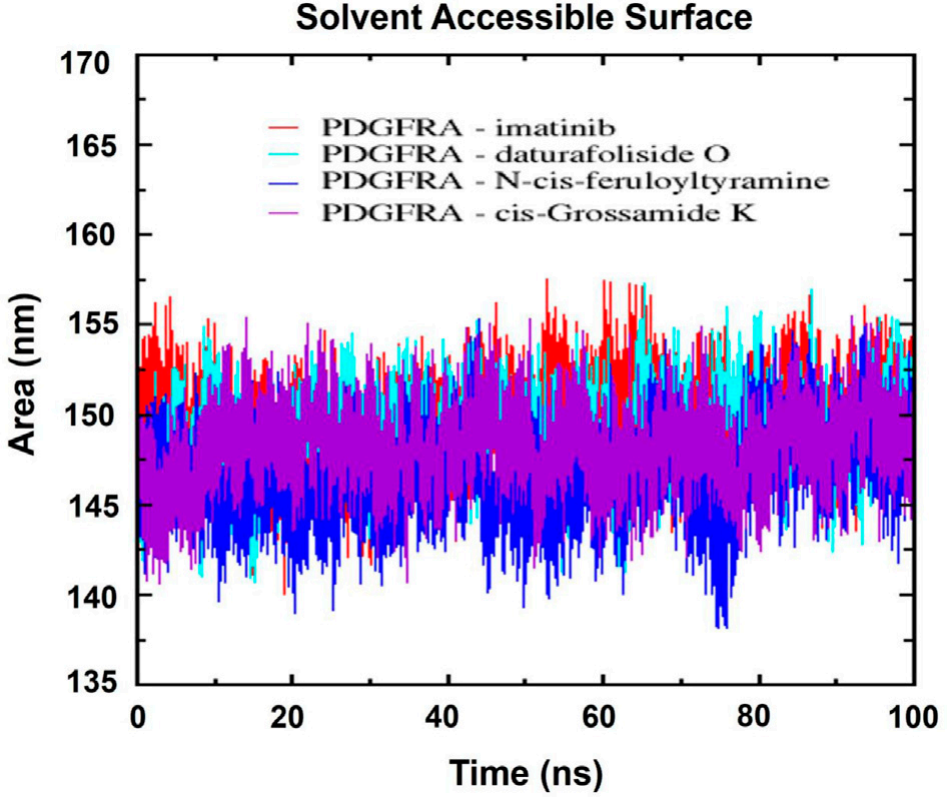
Solvent-accessible surface area (SASA) of the respective complexes: In red, PDGFRA-imatinib; in cyan, PDGFRA-daturafoliside O; in blue, PDGFRA-N-cis-feruloyltyramine; in violet, PDGFRA-cis-Grossamide K.

Next, the analysis of the solvent-accessible surface area (SASA) was performed to understand the dynamics of the hydrophilic and hydrophobic regions and the stability of the folding of the PDGFRA protein. The consistent average SASA values, observed in Figure 13 for the complexes of the compounds imatinib (149.61 nm^2^), daturafoliside O (148.76 nm^2^), N-cis-feruloyltyramine (146.58 nm^2^), and cis-Grossamide K (148.24 nm^2^), indicated more compact structures with reduced solvent exposure, suggestive of a stabilized protein conformation over 100 ns.

### ADMET screening

Understanding ADMET properties is critical for ensuring the overall safety and efficacy of drug candidates, as more than half of drug molecules are unable to be tested in clinical trials due to insufficient ADMET characteristics. 7 most active ligands that follow the standard druglikeness rules (Table 1) were chosen for ADMET analysis. ADMET characteristics of medicinal compounds were predicted using the ADMETlab 3.0 server. The studied parameters include P-gp inhibitor, Caco2 cell line permeability, human intestinal absorption (HIA), volume of distributions (VDss), BBB permeation metabolic enzyme inhibitors, total clearance, AMES toxicity, and carcinogenicity (Table 2).

**TABLE 2:** ADMET analysis of hit compounds.

Compound name	Absorption			Distribution		Metabolism		Excretion	Toxicity	
	P-gp inhibitor	Caco2 cells permeability (log papp) ×10–6	HIA	VDss	BBB permeability (cm/s)	CYP 1A2 inhibitor	CYP 3A4 inhibitor	Total clearance (mL/min/kg)	AMES toxicity	Carcino-genicity
*cis*-Grossamide K	0.625	−5.236	0.016	0.795	0.01	0.567	0.85	10.393	0.394	Inactive
Daturafoliside O	0.999	−4.779	0.006	0.62	0.509	0.021	0.926	9.875	0.019	Inactive
*N*-*cis*-feruloyltyramine	0.005	−4.733	0.015	0.564	0.076	0.946	0.75	13.105	0.144	Inactive
Maceneolignan H	0.999	−4.564	0.009	1.003	0.167	0.101	0.963	6.888	0.102	Inactive
Erythro-2-(4-allyl-2,6-dimethoxyphenoxy)-1-(3,4,5-trimethoxyphenyl) propan-1,3-dio*l*	0.93	−4.704	0.014	0.903	0.295	0.027	0.853	8.295	0.031	Inactive
Myrifralignan C	0.985	−4.683	0.009	0.589	0.176	0.473	0.648	6.449	0.014	Inactive
stigmasteryl-3-O-β-glucoside	0.971	−4.862	0.415	1.15	0.009	0.012	0.402	1.362	0.023	Inactive

PDGFRA inhibition can also decrease neurological defects, edema (brain swelling), and Evan blue extravasation within 1–3 days after intracerebral hemorrhage (ICB) (Ma et al., 2011). PDFRA inhibitors should cross the blood-brain barrier to reduce brain impairment. All the hit compounds predicted permeation for the blood-brain barrier (BBB). All seven compounds showed BBB + permeation value in the range of 0–1. P-glycoprotein (P-gp) is abundantly present in cancerous cells and removes chemotherapy drugs from inside the cells. An anti-cancer drug should block P-gp to prevent it from expelling chemotherapy drugs out of the cells. The value of P-gp inhibitors should be in between 0 and 1, and all hit compounds meet this criterion (Table 2). The capacity of oral medicine to pass through the Caco-2 cell line, which originates from human colorectal adenocarcinoma epithelial cells, is used to assess absorption parameters. A high Caco-2 permeability score (−5.15 log cm/s) suggests the drug is absorbed efficiently (Awortwe et al., 2014). Compound 1 *cis*-Grossamide K showed a CaCO_2_ permeability value of −5.23, indicating higher absorption in the body. All top hits showed CaCO_2_ values greater than −4.5 log cm/s.

The drugs show good intestinal absorption in humans if their value is from 0 to 0.3. Notably, our hit compounds fall in this range (Table 2). VDss reveals how the drug is dispersed throughout the body, with more remaining in the plasma. A low VDss suggests strong water solubility or high plasma protein binding. In contrast, a high VDss shows a high concentration in tissues, possibly due to high lipid solubility or tissue binding (Obach et al., 2008). Log L/kg < -0.15 implies low VDss, whereas log L/kg > 0.45 suggests excellent VDss. Compounds 1, 2, 3, 5, and 6 showed optimal VDss values, while compound 4 and 7 showed the highest VDss values greater than 1 (Table 2).

CYP1A2 is mostly present in the liver and plays a role in the metabolism of a variety of drugs, the activation of heterocyclic and aryl amines, which can cause cancer. The most common CYP enzyme present in the small intestine and liver, CYP3A4, is particularly intriguing because it has been proven to catalyze the metabolism of over half of all therapeutic medicines (Yim et al., 2020). Compounds showed values above 0.8 are good inhibitors of these metabolic enzymes (Table 2). Clearance is calculated by dividing the amount of drug in the plasma (measured in mg/min) by its concentration (measured in mg/mL). The total clearance capacity of the body is determined by the sum of drug clearance from the plasma by the liver, kidneys, and other organs. The top 3 compounds, cis-Grossamide K, Daturafoliside O, and N-cis-feruloyltyramine, have the highest clearance value from the body, while all compounds showed a good clearance value greater than 1 mL/min/kg. The AMES test is used to predict the toxicity of chemical compounds. Compounds with an AMES value greater than 0.1 are likely to be non-toxic. Compounds 1, 3, and 4 are found to be non-toxic, among others. All hits showed negative results for the carcinogenic test (Table 2). This analysis helped identify the safe, efficient, and non-toxic compounds to be used as PDGFRA inhibitors.

### Biological activity prediction

One of the significant characteristics of a chemical compound is its biological activity, which is caused by their interaction in a biological system. The PASS training set includes a list of the activities and properties that each organic chemical may exhibit under optimum conditions. The depicted activity spectra are represented in PASS as an ordered list of activities with the probabilities “to be active” (Pa) and “to be inactive” (Pi). We filtered the top 3 hits cis-Grossamide K, Daturafoliside O, and N-cis-feruloyltyramine for PASS prediction (Supplementary Figure S4). A high Pa value for any biological activity suggests that the compound is very probable to have that particular trait.

Compound 1, cis-Grossamide K, showed free radical scavenger and antioxidant properties with a *p*-value of 0.827 and 0.741, respectively, to protect the human body from the harmful effects of free radicals. This compound also possesses HIF1-alpha inhibition activity and appears to block hypoxia-inducible factor 1-alpha (HIF1A) expression, which may result in lowered expression of HIF1A downstream target genes important for tumor growth and survival, as well as a reduction in tumor cell proliferation [46]. Compound 2, Daturafoliside O, showed antineoplastic property to be used as an anti-cancer drug with a probability of 0.853. This compound also showed hepatoprotectant, antieczematic, and hepatic disorder treatment activity, among others (Supplementary Figure S4). Compound 3, N-cis-feruloyltyramine, showed preneoplastic activity to inhibit the formation of malignant tumors (Chodak et al., 1980) with a pa value of 0.812. This compound showed JAK2 expression inhibition activity to treat inflammatory conditions such as ulcerative colitis and rheumatoid arthritis (Miller et al., 2014). It also showed MMP9 expression inhibition activity to suppress the invasion, proliferation, and tumor cell growth to help in the therapy of thyroid cancer (Li et al., 2023). These predictions might help to confirm the promising activities of our hits within the human body.

### Medicinal chemistry

Following the ADMET study of the top 7 hits, we investigated their many medicinal attributes in biological experiments, including reactivity, potent response, and biosynthetic availability using SwissADME (Supplementary Table S3). PAINS (pan-assay interference compounds) are promiscuous or hitter substances that contain substructures demonstrating strong activity in biological assays regardless of the main target (Prabha and Ezhilarasi, 2021). Our all hits showed activity only against the primary target PDGFRA, as they showed no alerts against the PAIN assay. This indicates that they are likely to exhibit specific biological activity, which is crucial for targeting specific therapeutic pathways. Brenk detects molecules with acceptable toxicity, metabolic stability, and appropriate chemical reactivity (Prabha and Ezhilarasi, 2021). All top compounds showed one alert for the Brenk assay, while compound 6 didn’t show any alert, suggesting they possess suitable chemical reactivity, metabolic stability, and acceptable toxicity levels, essential for safe and effective drug design. Compound 3 completely followed the lead-likeness rule, while the others showed two or three violations. Synthetic accessibility refers to how easy it is to synthesize compounds. However, all of the leading drug candidates have a high synthetic accessibility score (SAS) (Supplementary Table S3), suggesting these compounds can be synthesized relatively easily, facilitating their production and further development. Overall, these results underscore the strong potential of the identified compounds in medicinal chemistry, highlighting their suitability for further exploration as therapeutic agents with favorable medicinal properties.

## Conclusion

This research is invaluable for identifying worthwhile hits and is a significant advancement in the development of PDGFRA inhibitor drug design. Through a series of advanced *in silico* techniques, our study suggests that the seven leading natural products, including cis-Grossamide K, Daturafoliside O, N-cis-feruloyltyramine, Maceneolignan H, Erythro-2-(4-allyl-2, 6-dimethoxyphenoxy)-1-(3, 4, 5-trimethoxyphenyl) propan-1, 3-diol, Myrifralignan C, and stigmasteryl-3-O-β-glucoside, might serve as potential inhibitors for cancer. These compounds showed docking scores higher than standard imatinib, Crenolanib, Lenavatinib, and Sofarenib. The detailed ligand-protein interaction analysis of top hits demonstrated their strong affinity in the binding pocket of PDGFRA protein, including frequently binding residues, i.e., Leu660, Arg817, and Asp836. The MD simulations confirmed the stability of the top 3 hits, namely cis-Grossamide K, Daturafoliside O, and N-cis-feruloyltyramine. The druglikeness and pharmacokinetic analysis of our compounds evaluate their ability to be safe and efficient drugs. Additionally, pharmacokinetic analysis, PASS prediction, and medicinal characteristics calculation contributed to their detailed biological activity. Notably, among our top hits, N-cis-feruloyltyramine has the potential to become a lead compound according to the results of the lead-likeness analysis. The efficiency and least toxic effects of our hits demand the experimental studies to prevent tumor malignancies.

## Data Availability

The original contributions presented in the study are included in the article/Supplementary Material, further inquiries can be directed to the corresponding authors.
