# Supplementary material for: Computer-assisted discovery of natural inhibitors for platelet-derived growth factor alpha as novel therapeutics for thyroid cancer
Source: Front Pharmacol. 2025 Jan 9;15:1512864. doi: 10.3389/fphar.2024.1512864 (PMC11754405; doi:10.3389/fphar.2024.1512864)
Supplement: Supplementary file 1 [file Table1.docx]

**Table S1.** Phytochemicals collected from three ayurvedic anticancer plants namely *S. chirayita* (Roxb.) H. Karst. [Gentianaceae], *D. metel* L. [Solanaceae], and *M. fragrans* Houtt. [Myristicacea] showed docking score against PDGFRA (6JOL).

| **Sr. No.** | **Compound Name** | **Structures** | | **Docking Score (S)** | **Plant name** | **References** |
| --- | --- | --- | --- | --- | --- | --- |
|  | Standard Imatinib |  | | -7.49 | Tradescantia | (Pichler et al., 2014) |
|  | Nonacosanyl hentriacontanoate |  | | -11.5081682 | Swertia Chirayita | (Pant et al., 2002) |
|  | Trimyristin |  | | -10.3942289 | Myristica Fragrans | (Ha et al., 2020) |
|  | Glycerine-1,3-dimyristate |  | | -10.2102823 | Myristica Fragrans | (Ha et al., 2020) |
|  | Melongenamide D |  | | -9.93865681 | Datura Metel | (B.Y. Yang, H.B. Jiang, Y. Liu, Z.P. Xu, 2018) |
|  | (+)-Lariciresinol-9-*O*-*β*-D-glucopyranoside |  | | -9.74741268 | Datura Metel | (B.Y. Yang, H.B. Jiang, Y. Liu, Z.P. Xu, 2018) |
|  | Daturametelin G-Ac |  | | -9.62409115 | Datura Metel | (SHINGU et al., 1989) |
|  | quercetin 3-O-α-l-rhamnopyranosyl-(1 → 6)-O-[α-l-rhamnopyranosyl-(1 → 2)]-O-β-d-galactopyranoside |  | | -9.59349155 | Myristica Fragrans | (Morikawa et al., 2016a) |
|  | grossamide K |  | | -9.49544907 | Myristica Fragrans | (B. Yang, Xia, Wang, Dou, Fitoterapia, et al., 2010) |
|  | Tribulusamide A |  | | -9.47209072 | Datura Metel | (B. Yang et al., 2020) |
|  | Cannabisin F |  | -9.4435215 | | Datura Metel | (B.Y. Yang, Y.M. Luo, Y. Liu, X. Yin, Y.Y. Zhou, 2018) |
|  | Quercetin 3-*O*-2-(*E*-caffeoyl)- *α*-L-arabinopyranosyl-(1 → 2)- *β*-D-glucopyranoside-7-*O*-*β*-D-glucoside |  | -9.1920929 | | Datura Metel | (Alam et al., 2020) |
|  | Meteloside D |  | -9.14012432 | | Datura Metel | (Mai, Cuc, Anh, Nhiem, Steroids, et al., 2017) |
|  | Meteloside G |  | -9.09961224 | | Datura Metel | (Barclay, 1995) |
|  | Alangisesquin A |  | -9.08079243 | | Datura Metel | (B.Y. Yang, H.B. Jiang, Y. Liu, Z.P. Xu, 2018) |
|  | *erythro*-Buddlenol B |  | -9.07159042 | | Datura Metel | (B.Y. Yang, H.B. Jiang, Y. Liu, Z.P. Xu, 2018) |
|  | Grossamide |  | -9.05531216 | | Datura Metel | (B. Yang, Xia, Wang, Dou, Fitoterapia, et al., 2010) |
|  | Thoreliamide C |  | -9.02619457 | | Datura Metel | (B.Y. Yang, H.B. Jiang, Y. Liu, Z.P. Xu, 2018) |
|  | (*E*, *E*)-*N*, *N*-dityramin-4,4′ - dihydroxy-3,5′ -dimethoxy-*β*,3′ - bicinnamamide |  | -8.97947311 | | Datura Metel | (B.Y. Yang, H.B. Jiang, Y. Liu, Z.P. Xu, 2018) |
|  | *cis*-Cannabisin E |  | -8.94891071 | | Datura Metel | (B.Y. Yang, H.B. Jiang, Y. Liu, Z.P. Xu, 2018) |
|  | 7-Hydroxy-1-(4-hydroxy-3- methoxyphenyl)-*N2, N3*-bis(4- hydroxyphen-ethyl)-6-methoxy- 1,2-dihydronaphthalene-2,3- dicarboxamide |  | -8.89111233 | | Datura Metel | (B.Y. Yang, H.B. Jiang, Y. Liu, Z.P. Xu, 2018) |
|  | Meteloside C |  | -8.88976097 | | Datura Metel | (Mai, Cuc, Anh, Nhiem, Steroids, et al., 2017) |
|  | Melongenamide B |  | -8.88903809 | | Datura Metel | (B. Yang et al., 2020) |
|  | *N*1, *N*5, *N*10-tri-*p*-coumaroylspermidine |  | -8.69872665 | | Datura Metel | (B.Y. Yang, H.B. Jiang, Y. Liu, Z.P. Xu, 2018) |
|  | Cannabisin E |  | -8.68862724 | | Datura Metel | (B.Y. Yang, H.B. Jiang, Y. Liu, Z.P. Xu, 2018) |
|  | Kaempferol-3-*O*- *β*-glucopyranosyl (1 → 2)- *β*-glucopyranose-7-*O*- *α*-rhamnopyranoside |  | -8.67050266 | | Datura Metel | (Mai, Cuc, Anh, Nhiem, …, et al., 2017) |
|  | Cannabisin G |  | -8.64972591 | | Datura Metel | (B. Yang et al., 2020) |
|  | Kaurane acid glycoside A |  | -8.60994911 | | Datura Metel | (B. you Yang et al., 2018) |
|  | (+) (7*R*,7′ *R*,7′′ *R*,7′′′ *R*,8*S*,8′ *S*,8′′ *S*,8′′′ *S*)- 4′′ ,4′′′ -Dihydroxy-3,3′ ,3′′ ,3′′′ ,5,5′ - hexamethoxy-7,9′ ; 7′ ,9-diepoxy- 4,8′ ′ ; 4′ ,8′′′ -bisoxy-8,8′ - dineolignan-7′′ ,7′′′ ,9′′ ,9′′′ -tetraol |  | -8.51389217 | | Datura Metel | (B.Y. Yang, H.B. Jiang, Y. Liu, Z.P. Xu, 2018) |
|  | Ginsenoside Rg1 |  | -8.46891975 | | Datura Metel | (Liu et al., 2022a) |
|  | Daturafoliside J |  | -8.43344307 | | Datura Metel | (B. Yang, Guo, Li, Wu, et al., 2014) |
|  | (-S)-Secoisolariciresinol-4-*O*-*β*-D-glucopyranoside |  | -8.41367722 | | Datura Metel | (B.Y. Yang, H.B. Jiang, Y. Liu, Z.P. Xu, 2018) |
|  | Kaempferol-3-*O*-*β*-D-glucopyranose(l→2)-*β*-D-glucopyranoside-7-*O*-*β*-D-glucopyranoside |  | -8.40497875 | | Datura Metel | (Z.Y. Li, H.X. Kuang, Y.G. Xia, 2010) |
|  | Canabisine H |  | -8.38635254 | | Datura Metel | (X.-Y. Wang et al., 2019) |
|  | (7*S*,8*R*)-Dehydrodiconiferyl alcohol 9′ -*O*-*β*-glucopyranoside |  | -8.38023186 | | Datura Metel | (B.Y. Yang, H.B. Jiang, Y. Liu, Z.P. Xu, 2018) |
|  | Olean-12-ene-28-carboxy-3-*β*-hexadecanoate |  | -8.32419586 | | Swertia Chirayita | (Woo et al., 2019) |
|  | Daturafoliside D |  | -8.3123045 | | Datura Metel | (B. Yang, Guo, Li, Wu, et al., 2014) |
|  | Daturmetelide P |  | -8.30775547 | | Datura Metel | (J. Tan, Liu, Cheng, Sun, Pan, Guan, et al., 2020) |
|  | Octacosane |  | -8.29368305 | | Myristica Fragrans | (Ha et al., 2020) |
|  | Cannabisin D |  | -8.27310181 | | Datura Metel | (B.Y. Yang, H.B. Jiang, Y. Liu, Z.P. Xu, 2018) |
|  | Hyuganoside IIIb |  | -8.26860523 | | Datura Metel | (B.Y. Yang, C.L. Yang, Y. Liu, 2017) |
|  | Sargentodoside D |  | -8.26598835 | | Datura Metel | (B.Y. Yang, C.L. Yang, Y. Liu, 2017) |
|  | Glycopentoside F |  | -8.24516296 | | Datura Metel | (B.Y. Yang, H.B. Jiang, Y. Liu, Z.P. Xu, 2018) |
|  | Cannabisin H |  | -8.23823643 | | Datura Metel | (B. Yang et al., 2020) |
|  | Daucosterol |  | -8.2318573 | | Myristica Fragrans | (Hou et al., 2012) |
|  | Daturafoliside U |  | -8.187006 | | Datura Metel | (B. Yang, Guo, Li, Wu, et al., 2014) |
|  | Daturafoliside H |  | -8.17997074 | | Datura Metel | (B. Yang, Guo, Li, Wu, et al., 2014) |
|  | Swertiachiridiol A |  | -8.17686367 | | Swertia Chirayita | (Zhou et al., 2015) |
|  | Conicaoside |  | -8.16494274 | | Datura Metel | (B. Yang et al., 2007) |
|  | *cis*-Grossamide K |  | -8.16489506 | | Datura Metel | (B.Y. Yang, H.B. Jiang, Y. Liu, Z.P. Xu, 2018) |
|  | Dioscoroside D |  | -8.16355801 | | Datura Metel | (Mai, Cuc, Anh, Nhiem, Steroids, et al., 2017) |
|  | Daturmetelide N |  | -8.11526108 | | Datura Metel | (J. Tan, Liu, Cheng, Sun, Pan, Guan, et al., 2020) |
|  | (+)-Pinoresinol-*O*-*β*-D-diglucopyranoside |  | -8.09297371 | | Datura Metel | (B. Yang, Luo, et al., 2018) |
|  | Daturafoliside R |  | -8.08987427 | | Datura Metel | (Vermillion et al., 2011) |
|  | Notoginsenosides R1 |  | -8.08814621 | | Datura Metel | B. Yang, Yang, et al., 2018) |
|  | Daturafoliside Y |  | -8.05879116 | | Datura Metel | (B. Y. Yang et al., 2014) |
|  | 3-*β*-Hydroxy-11-oxo-olean-12-enyl-3-palmitate |  | -8.05272579 | | Swertia Chirayita | (Woo et al., 2019) |
|  | (22*R*)-27-Hydroxy-7*α*- methoxy-1-oxowitha-3,5,24- trienolide-24-*O*-*β*-*D*-glucoside |  | -8.0506115 | | Datura Metel | (B. Y. Yang et al., 2014) |
|  | Hepatacosane |  | -8.04752636 | | Myristica Fragrans | (Ha et al., 2020) |
|  | Meteloside B |  | -8.04464722 | | Datura Metel | (Mai, Cuc, Anh, Nhiem, Steroids, et al., 2017) |
|  | Meteloside A |  | -8.03649521 | | Datura Metel | (Mai, Cuc, Anh, Nhiem, Steroids, et al., 2017) |
|  | Daturafoliside E |  | -8.01592445 | | Datura Metel | (B. Yang, Guo, Li, Wu, et al., 2014) |
|  | Quercetin 3-*O*-rutinoside-7-*O*-glucoside |  | -8.00828457 | | Datura Metel | (Alam et al., 2020) |
|  | Daturataturin B |  | -7.99459076 | | Datura Metel | (B. Yang, Guo, Li, Wu, et al., 2014) |
|  | Dmetelin C (terpenoid) |  | -7.9910326 | | Datura Metel | (J. Tan, Liu, Cheng, Sun, Pan, Fitoterapia, et al., 2020) |
|  | Kaempferol-3-*O*-*α*-L-rhamnopyranose(l→6)-*β*-D-glucopyranoside-7-*O*-*β*-D-glucopyranoside |  | -7.98050833 | | Datura Metel | (Z.Y. Li, H.X. Kuang, Y.G. Xia, 2010) |
|  | kaurane daturoside A |  | -7.97321987 | | Datura Metel | (Liu et al., 2022b) |
|  | Methyl-(*Z*)-9-octadecenoate |  | -7.95185995 | | Datura Metel | (Mahendran et al., 2022) |
|  | Cherry glycoside |  | -7.94389486 | | Datura Metel | (Alam et al., 2020) |
|  | Stigmasterol-3-*O*-*β*-D-glucoside |  | -7.93716002 | | Datura Metel | (Alam et al., 2020) |
|  | Meteloside E |  | -7.92742443 | | Datura Metel | (Mai, Cuc, Anh, Nhiem, Steroids, et al., 2017) |
|  | Hexacosane |  | | -7.91988039 | Myristica Fragrans | (Ha et al., 2020) |
|  | Kaempferol-3,7-*O*-diglucoside |  | -7.89832354 | | Datura Metel | (Mai, Cuc, Anh, Nhiem, …, et al., 2017) |
|  | Daturafoliside O |  | -7.89528465 | | Datura Metel | (B. Yang, Guo, Li, Wu, et al., 2014) |
|  | Kaempferol-3-*O*-*β*-D-glucosyl (l→2)-*β*-D-galactoside-7-*O*-*β*-D-glucoside |  | -7.86399889 | | Datura Metel | (Mai, Cuc, Anh, Nhiem, …, et al., 2017) |
|  | Daturafoliside P |  | -7.86059141 | | Datura Metel | (B. Yang, Guo, Li, Wu, et al., 2014) |
|  | Giganteone A |  | -7.85717201 | | Myristica Fragrans | (Pandey et al., 2016) |
|  | Astragaloside II |  | -7.84905148 | | Datura Metel | (Liu et al., 2022a) |
|  | *N*-*cis*-feruloyltyramine |  | -7.83695698 | | Datura Metel | (B. Yang, Xia, Wang, Dou, Fitoterapia, et al., 2010) |
|  | cannabisin A |  | -7.8355341 | | Datura Metel | (Seong Choi et al., 2016) |
|  | Herpetol C |  | -7.81381178 | | Datura Metel | (B.Y. Yang, H.B. Jiang, Y. Liu, Z.P. Xu, 2018) |
|  | (7*S*,8*R*,7′ *S*,8′ *S*)-4,9,4′ ,7′ - Tetrahydroxy-3,3′ -dimethoxy-7,9′ - epoxylignan-4-*O*-*β*-D-glucopyranoside |  | -7.81139278 | | Datura Metel | (Mai, Cuc, Anh, Nhiem, …, et al., 2017) |
|  | Iariciresinol-4’-O-β-D-glucopyranoside |  | -7.8061986 | | Datura Metel | (B.Y. Yang, C.L. Yang, Y. Liu, 2017) |
|  | Chenoalbicin |  | | -7.80338192 | Datura Metel | (B. Yang, Xia, Wang, Dou, Fitoterapia, et al., 2010) |
|  | Erythrodiol-3-*O*-palmitate |  | -7.79557133 | | Swertia Chirayita | (Woo et al., 2019) |
|  | (−)-1-(2,6-Dihydroxyphenyl)-9-[4-hydroxy-3-(p-menth-1-en-8- oxy)-phenyl]-1-nonanone |  | -7.7888217 | | Myristica Fragrans | (Duan et al., 2009a) |
|  | Cannabisin L |  | -7.78314018 | | Datura Metel | (B.Y. Yang, H.B. Jiang, Y. Liu, Z.P. Xu, 2018) |
|  | Quercetin-3-*O*-rutinoside |  | -7.77988768 | | Datura Metel | (Alam et al., 2020) |
|  | Grossamide K |  | -7.74904966 | | Datura Metel | (B.Y. Yang, H.B. Jiang, Y. Liu, Z.P. Xu, 2018) |
|  | Baimantuoluoside B |  | -7.74809504 | | Datura Metel | (H. Kuang et al., 2009) |
|  | Baimantuoluoside H |  | -7.74706268 | | Datura Metel | (B. Y. Yang et al., 2014) |
|  | (1*S,*2*R*)-2-(4-Allyl-2,6-dimethoxyphenoxy)-1-(3,4-dimethoxyphenyl) propan-1-ol acetate |  | -7.72481012 | | Myristica Fragrans | (Kang et al., 2013) |
|  | Amaroswerin |  | | -7.71740627 | Swertia Chirayita | (Suryawanshi et al., 2006) |
|  | Officinalioside |  | -7.71199656 | | Datura Metel | (B.Y. Yang, H.B. Jiang, Y. Liu, Z.P. Xu, 2018) |
|  | Daturafoliside X |  | -7.70500851 | | Datura Metel | (B. Y. Yang et al., 2014) |
|  | Daturamalakoside A |  | -7.70026398 | | Datura Metel | (Bellila et al., 2011) |
|  | Daturametelin I |  | -7.68355751 | | Datura Metel | (B.Y. Yang, H.B. Jiang, Y. Liu, Z.P. Xu, 2018) |
|  | 6’-*O*-*β*-D-glucopyranosyl sweroside |  | -7.65501022 | | Swertia Chirayita | (Zhou et al., 2015) |
|  | Leptolepisol D |  | -7.64016151 | | Datura Metel | (B.Y. Yang, C.L. Yang, Y. Liu, 2017) |
|  | Baimantuoluoline G |  | -7.63387537 | | Datura Metel | (H. Kuang et al., 2011) |
|  | Kaempferol 3-*O*-rutinoside-7-*O*-glucoside |  | -7.61603451 | | Datura Metel | (Mai, Cuc, Anh, Nhiem, …, et al., 2017) |
|  | Icariside E5 |  | -7.59516621 | | Datura Metel | (B.Y. Yang, C.L. Yang, Y. Liu, 2017) |
|  | Daturmetelide R |  | -7.59461689 | | Datura Metel | (J. Tan, Liu, Cheng, Sun, Pan, Guan, et al., 2020) |
|  | Dehydrodiconiferyl alcohol 4-*O*- *β*-D-glucopyranoside |  | -7.59122658 | | Datura Metel | (B.Y. Yang, H.B. Jiang, Y. Liu, Z.P. Xu, 2018) |
|  | Maceneolignan H [(−)*-erythro*-(1*R,*2*S*)-2-(4-allyl-2,6-  dimethoxyphenoxy)-1-(3,4-dimethoxyphenyl) propan-  1-ol acetate] |  | -7.58749104 | | Myristica Fragrans | (Morikawa et al., 2016b) |
|  | Astrojanoside A |  | -7.58610344 | | Datura Metel | (Liu et al., 2022a) |
|  | Lariciresinol-4′ -*O*-*β*-D-glacoside |  | -7.58293581 | | Datura Metel | (B.Y. Yang, H.B. Jiang, Y. Liu, Z.P. Xu, 2018) |
|  | Meteloside F |  | -7.578053 | | Datura Metel | (Barclay, 1995) |
|  | 7*R*,8*R*-*threo*-4,7,9-Trihydroxy-3,3′ - dimethoxy-8-*O*-4′ -neolignan-9′ -*O*- *β*-*D*-glucopyranoside |  | -7.57598257 | | Datura Metel | (B.Y. Yang, C.L. Yang, Y. Liu, 2017) |
|  | Kaempferol-3-*O*-*β*-D-glucopyranose(l→2)-*β*-D-glucopyranoside-7-*O*-*α*-L-rhamnopyranoside |  | -7.57418251 | | Datura Metel | (Z.Y. Li, H.X. Kuang, Y.G. Xia, 2010) |
|  | Daturmetelide Q |  | -7.56227541 | | Datura Metel | (J. Tan, Liu, Cheng, Sun, Pan, Guan, et al., 2020) |
|  | *Erythro-2-(4-allyl-2,6-dimethoxyphenoxy)-1-(3,4,5-trimethoxyphenyl) propan-1,3-diol* |  | -7.55743551 | | Myristica Fragrans | (HATTORI et al., 1986) |
|  | 3-*O*-Demethyl swertipunicoside |  | -7.55730057 | | Swertia Chirayita | (He et al., 2017) |
|  | (+)-Pinoresinol 4′′ -*O*-*β*-D-glucopyranoside |  | -7.53118849 | | Datura Metel | (Mai, Cuc, Anh, Nhiem, …, et al., 2017) |
|  | Daturafoliside F |  | -7.52357578 | | Datura Metel | (B. Yang, Guo, Li, Wu, et al., 2014) |
|  | Myrifralignan C |  | -7.51959276 | | Myristica Fragrans | (G. Cao et al., 2015) |
|  | 8-*O*-[*β*-D-xylopyranosyl-(1→6)-*β*-D-glucopyranosyl]-1-hydroxyl- 3,7-dimethoxy xanthone |  | -7.51948261 | | Swertia Chirayita | (Zhou et al., 2015) |
|  | (+)-Cycloolivil-4’-*O*-*β*-D-glucopyranoside |  | -7.51825857 | | Swertia Chirayita | (Zhou et al., 2015) |
|  | Kaempferol-3-*O*-*β*-D-glucopyranose (1 → 2)-*β*-D-glucopyranoside |  | -7.50578356 | | Datura Metel | (Andrade et al., 2011) |
|  | Iariciresinol-9-*O*-*β*-D-glucopyranoside |  | -7.49956799 | | Datura Metel | (B. Yang, Luo, et al., 2018) |
|  | stigmasteryl-3-O-β-glucoside |  | | -7.49479008 | Myristica Fragrans | (Zhanga et al., 2015) |
|  | Daturafoliside G |  | -7.48054504 | | Datura Metel | (B. Yang, Guo, Li, Wu, et al., 2014) |
|  | Baimantuoluoamide B |  | -7.46476507 | | Datura Metel | (B. Yang, Xia, Wang, Dou, Fitoterapia, et al., 2010) |
|  | Daturafoliside K |  | -7.46297932 | | Datura Metel | (B. Yang, Guo, Li, Wu, et al., 2014) |
|  | Daturametelin B |  | -7.45199871 | | Datura Metel | (Bellila et al., 2011) |
|  | Ilekudinoside C |  | -7.44934607 | | Datura Metel | (Mai, Cuc, Anh, Nhiem, Steroids, et al., 2017) |
|  | (9*E*)-8,11,12- trihydroxyoctadecenoic acid methyl ester |  | -7.44701242 | | Datura Metel | (H. Gan, J. He, X.Y. Wang, Y.W. Wang, H.M. Gao, H. Jiang, W.K. Zhang, 2021) |
|  | Isovitexin |  | -7.43432426 | | Swertia Chirayita | (He et al., 2017) |
|  | Lyciumamide K |  | | -7.43179512 | Datura Metel | (S. Wang et al., 2021) |
|  | Nicglycoside C |  | -7.42072487 | | Datura Metel | Tan et al. (2022) |
|  | Dmetelisporoside A |  | -7.40602779 | | Datura Metel | (Bajpai et al., 1991) |
|  | Dinoxin B |  | -7.39947176 | | Datura Metel | (Vermillion et al., 2011) |
|  | Daturameteloside I |  | -7.38581228 | | Datura Metel | (B. Y. Yang, 2005) |
|  | Isoorientin |  | -7.37983942 | | Swertia Chirayita | (Zhou et al., 2015) |
|  | (7*R*,8*S*,7′ *S*,8′*R*)-4,9,4′ ,7′ - Tetrahydroxy-3,3′ -dimethoxy-7,9′ - epoxylignan-4-*O*-*β*-D-glucopyranoside |  | -7.37940502 | | Datura Metel | (Mai, Cuc, Anh, Nhiem, …, et al., 2017) |
|  | 1-(2,6-Dihydroxyphenyl)-9-  [4-hydroxy-3-(p-menth-1-en-8-yloxy)phenyl]-1-nonanone |  | -7.36975431 | | Myristica Fragrans | (Duan et al., 2009b) |
|  | Isomangostin |  | -7.35562611 | | Swertia Chirayita | (Reyes-Escogido et al., 2011) |
|  | Maceneolignan C |  | -7.34016418 | | Myristica Fragrans | (Morikawa et al., 2016a) |
|  | Daturafoliside T |  | -7.33332729 | | Datura Metel | (J. Tan, Liu, Cheng, Sun, Pan, Guan, et al., 2020) |
|  | Daturmetesides C |  | -7.32676315 | | Datura Metel | (J. Tan, Liu, Cheng, Sun, Liu, et al., 2020) |
|  | (+)-Licarin A |  | -7.32672405 | | Myristica Fragrans | (Morikawa et al., 2016a) |
|  | Chiratanin |  | -7.32253695 | | Swertia Chirayita | (Mandal & Chatterjee, 1987) |
|  | Lariciresinol-4′ -*O*-*β*-D-glucopyranoside |  | -7.32072783 | | Datura Metel | (B.Y. Yang, H.B. Jiang, Y. Liu, Z.P. Xu, 2018) |
|  | Daturafoliside I |  | -7.31416893 | | Datura Metel | (B. Y. Yang et al., 2014) |
|  | Erythro-(7S,8R)-D80-7-acetoxy-  3,4,3050-tetramethoxy-8-O-40-neolignan |  | -7.31258202 | | Myristica Fragrans | (Ha et al., 2020) |
|  | (6*Z*)-4-(3-Hydroxybutylidene)- 3,5,5-trimethyl-2-cyclohexene- 1-one-*O-β*-D-glucopyranoside |  | -7.30991077 | | Datura Metel | (Pan, Wang, & Hu, 2007) |
|  | Daturmetelide U |  | -7.30612087 | | Datura Metel | (J. Tan, Liu, Cheng, Sun, Pan, Guan, et al., 2020) |
|  | raphidecursinol B |  | -7.28888273 | | Myristica Fragrans |  |
|  | (*Z*)-3-Hexenyl-*O*-*α*-arabino-pyranosyl-(1 → 6)-*O*-*β*-D-glucopyranoside |  | | -7.28161669 | Datura Metel | Tan et al. (2022) |
|  | (*7S,8S,7*′*R,8*′*R*)-3,3′,4,4′,5,5′-hexamethoxy-7,7′,8,8′-  lignan |  | -7.2699976 | | Myristica Fragrans |  |
|  | Myristicanol A |  | -7.25451851 | | Myristica Fragrans | (G.-Y. Cao et al., 2015) |
|  | Cyclosieversioside F |  | -7.25415611 | | Datura Metel | (Liu et al., 2022a) |
|  | Methyl 3,4-dihydroxyphenylacetate-4-O-[2-O-β-D-apisoyl-6-O-(2-hydroxybenzoyl)]-β-D-glucopyranoside |  | | -7.25347853 | Datura Metel | (B. Yang et al., 2020) |
|  | **\**Daturafoliside L |  | -7.24861479 | | Datura Metel | (B. Yang, Guo, Li, Wu, et al., 2014) |
|  | (7S,8R)-2-(4-allyl-2,6-dimethoxyphenoxy)-1-(4- hydroxy-3,5-dimethoxyphenyl)-propan-1-ol |  | -7.23891973 | | Myristica Fragrans | (Ha et al., 2020) |
|  | Daturameteloside H |  | -7.23491812 | | Datura Metel | (B. Yang, Xia, Wang, Dou, research, et al., 2010) |
|  | 5′ -Methoxy lariciresinol |  | -7.23264074 | | Datura Metel | (B.Y. Yang, H.B. Jiang, Y. Liu, Z.P. Xu, 2018) |
|  | Docosane |  | -7.23258018 | | Myristica Fragrans | (Ha et al., 2020) |
|  | Methyl Oleate |  | -7.22721434 | | Myristica Fragrans | (Ha et al., 2020) |
|  | Ethyl Palmitate |  | -7.22153282 | | Myristica Fragrans | (Ha et al., 2020) |
|  | Linoleic Acid |  | -7.22108507 | | Myristica Fragrans | (Ha et al., 2020) |
|  | 1-Hydroxy-2,3,4,5-tetramethoxy xanthone |  | -7.21818352 | | Swertia Chirayita | (Shi et al., 2005) |
|  | Malabaricone A |  | -7.2177434 | | Myristica Fragrans | (Pandey et al., 2016) |
|  | Trans-beta-Farnesne |  | -7.20312738 | | Myristica Fragrans | (Ha et al., 2020) |
|  | (−)-(8R)-∆8′ - 3,4,5,3′,5′-pentamethoxy-8-O-4′-neolignan |  | -7.19772291 | | Myristica Fragrans | (Isogai et al., 2014) |
|  | 7-*O*-[*β*-D-xylopyranosyl-(1→2)-*β*-D-xylopyranosyl]-1,8-dihydroxy- 3-methoxy xanthone |  | -7.19114065 | | Swertia Chirayita | (Zhou et al., 2015) |
|  | Daturglycoside C |  | -7.19033718 | | Datura Metel | Tan et al. (2022) |
|  | Swertianolin |  | -7.17831755 | | Swertia Chirayita | (L. Cai, S. Wang, T. Li, 2006) |
|  | Stearic Acid |  | -7.17466974 | | Myristica Fragrans | (Ha et al., 2020) |
|  | Daturmetelide V |  | -7.16055346 | | Datura Metel | c |
|  | 1,5,6-Trihydroxy-3-methoxy xanthone |  | -7.15939188 | | Swertia Chirayita | (Mandal & Chatterjee, 1987) |
|  | 1-Hydroxy-2,3,4,6-tetramethoxy xanthone |  | -7.1533494 | | Swertia Chirayita | (Shi et al., 2013) |
|  | (7S,8R)-2-(4-allyl2,6-dimethoxy-phenoxy)-1-(3,4,5-trimethoxyphenyl)-propan-1-ol |  | | -7.14485216 | Myristica Fragrans | (Ha et al., 2020) |
|  | 2-(4-Allyl-2,6-dimethoxyphenoxy)-1-(3,4-dimethoxyphenyl) propane |  | -7.13557529 | | Myristica Fragrans | (G.-Y. Cao et al., 2015)ccccc |
|  | Petroselinic Acid |  | -7.13323402 | | Myristica Fragrans | (Ha et al., 2020) |
|  | (+)-Δ8′-7-acetoxy-3,4,3′,5′-tetramethoxy-8-O-4′-neolignan |  | -7.13162947 | | Myristica Fragrans | (Ha et al., 2020) |
|  | (1R,2S)-1-(3,4,5-Trimethoxyphenyl)-2-(2-methoxy-4-(1(E)-propenyl) phenoxy) propan-1-ol |  | -7.1311326 | | Myristica Fragrans | (G.-Y. Cao et al., 2015) |
|  | Daturametelin F |  | | -7.13056898 | Datura Metel | (SHINGU et al., 1989) |
|  | (6*E*)-4-(3-Hydroxybutylidene)- 3,5,5-trimethyl-2-cyclohexene- 1-one-*O-β*-D-glucopyranoside |  | -7.12776184 | | Datura Metel | (Pan, Wang, & Hu, 2007) |
|  | Daturafoliside C |  | -7.12072515 | | Datura Metel | (B. Yang, Guo, Li, Wu, et al., 2014) |
|  | (1S,2R)-2-(4-Allyl-2,6-dimethoxyphenoxy)-1-(4-hydroxy-3-methoxyphenyl) propan-1-ol acetate |  | -7.12034321 | | Myristica Fragrans | (Kang et al., 2013) |
|  | Daturmetelide O |  | -7.11410189 | | Datura Metel | (J. Tan, Liu, Cheng, Sun, Pan, Guan, et al., 2020) |
|  | Scutebarbatine A |  | -7.10630703 | | Datura Metel | (X.-Y. Wang et al., 2019) |
|  | (*2S,3R*)-7-methoxy-3-methyl-5-((*E*)-prop-  1-enyl)-2-(5-methoxy,3,4-methylenedioxy phenyl)-2,3-dihydrobenzofuran |  | -7.09823227 | | Myristica Fragrans |  |
|  | Malabaricone B |  | -7.08450222 | | Myristica Fragrans | (Pandey et al., 2016) |
|  | Aurantiamide acetate |  | -7.06292868 | | Datura Metel | (X.-Y. Wang et al., 2019) |
|  | Daturmetelide F |  | -7.05973148 | | Datura Metel | (J. Tan, Liu, Cheng, Sun, Pan, Guan, et al., 2020) |
|  | 2-(4-Allyl-2,6-dimethoxyphenoxy)-1-(4-hydroxy-  3-methoxyphenyl) propane |  | -7.05892801 | | Myristica Fragrans | (HATTORI et al., 1987) |
|  | Maceneolignan A |  | -7.05391216 | | Myristica Fragrans | (Morikawa et al., 2016a) |
|  | Maceneolignan K |  | -7.0424552 | | Myristica Fragrans | (Morikawa et al., 2018) |
|  | *Erythro-2-(4-allyl-2,6-dimethoxyphenoxy)-1-(3-hydroxy-4,5-dimethoxyphenyl) propan-1-ol* |  | -7.02680635 | | Myristica Fragrans | (HATTORI et al., 1987) |
|  | Licarin A |  | -7.01448536 | | Myristica Fragrans | (S. Francis et al., 2019) |
|  | Dmetelin C |  | -7.01367235 | | Datura Metel | (B. Yang, Guo, Li, Liu, et al., 2014) |
|  | Amaronitidin |  | -7.01195192 | | Swertia Chirayita | (Kumar et al., 2015) |
|  | Myrifralignan B |  | -7.00299406 | | Myristica Fragrans | (G. Cao et al., 2015) |
|  | *S*)-1-(3,4,5-Trimethoxyphenyl)-2-(3-methoxy-5-(prop-  1-yl) phenyl)-propan-1-ol |  | -7.00154066 | | Myristica Fragrans | (Muñoz Acuña et al., 2016) |
|  | Maceneolignan F |  | -7.0006566 | | Myristica Fragrans | (Morikawa et al., 2016b) |
|  | Kaempferol-3-*O*-*β*-D-galactoside |  | | -6.99407291 | Datura Metel | (Andrade et al., 2011) |
|  | Octadecane |  | -6.98743439 | | Myristica Fragrans | (Ha et al., 2020) |
|  | Norswertianolin |  | -6.98568678 | | Swertia Chirayita | (L. Cai, S. Wang, T. Li, 2006) |
|  | Myrifralignan D |  | -6.9855423 | | Myristica Fragrans | (G. Cao et al., 2015) |
|  | Methyl-9,12,13- trihydroxyoctadeca-10,15- dienoate |  | | -6.97576809 | Datura Metel | (J. Y. Tan et al., 2021) |
|  | (*Z*)-3-Hexenyl-*O*-*β*-D-glucopyranosyl-(1"→2′ )-*β*-D-glucopyranoside |  | -6.97090435 | | Datura Metel | Tan et al. (2022) |
|  | (−)*-Erythro*-(1*R,*2*S*)-2-(4-allyl-2,6-dimethoxyphenoxy)-  1-(3,4-dimethoxyphenyl) propan-1-ol |  | | -6.97007847 | Myristica Fragrans | (Morikawa et al., 2016b) |
|  | Daturametelin D |  | -6.95632029 | | Datura Metel | (SHINGU et al., 1989) |
|  | 3-(4’-allyl-2’,6’-Dimethoxy-Phenoxy)-2-methyl-6-2,3-dihydro-benzofuran |  | -6.94017839 | | Myristica Fragrans | (Ha et al., 2020) |
|  | Stroside A |  | | -6.93642282 | Datura Metel | (B.Y. Yang, C.L. Yang, Y. Liu, 2017) |
|  | Daturmetelide T |  | | -6.93608046 | Datura Metel | (J. Tan, Liu, Cheng, Sun, Pan, Guan, et al., 2020) |
|  | Alkesterol B |  | -6.92799807 | | Datura Metel | (Z.Y. Li, H.X. Kuang, Y.G. Xia, 2010) |
|  | Daturmetesides A |  | -6.92630816 | | Datura Metel | (J. Tan, Liu, Cheng, Sun, Liu, et al., 2020) |
|  | Myrislignan |  | -6.92571115 | | Myristica Fragrans | (Chiu et al., 2016b) |
|  | Withametelins P |  | -6.92545843 | | Datura Metel | (Pan, Wang, products, et al., 2007) |
|  | Palmitic Acid |  | -6.92411852 | | Myristica Fragrans | (Ha et al., 2020) |
|  | Daturametelin E |  | -6.91771603 | | Datura Metel | (B. Yang, Guo, Li, Wu, et al., 2014) |
|  | Phenyl alcohol-*O*-*β*-D-glucopyranosyl (2 → 1)-*O*-*β*-D-glucopyranoside |  | -6.91341162 | | Datura Metel | (B. Y. Yang, 2005) |
|  | 1-Hydroxy3,5,7,8-tetramethoxy xanthone |  | -6.91326952 | | Swertia Chirayita | (You et al., 2017) |
|  | Daturafoliside B |  | -6.91210175S | | Datura Metel | (B. Yang, Guo, Li, Wu, et al., 2014) |
|  | (−)*-*(2*R*)-2-(4-allyl-2,6-dimethoxyphenoxy)-1-(3,4,5-  trimethoxyphenyl) propane |  | -6.9103508 | | Myristica Fragrans | (Morikawa et al., 2016b) |
|  | Dmetelin B |  | -6.90952206 | | Datura Metel | (J. Tan, Liu, Cheng, Sun, Pan, Fitoterapia, et al., 2020) |
|  | Malabaricone C |  | -6.90611315 | | Myristica Fragrans | (Rastegari et al., 2022) |
|  | 8’-*α*-hydroxyllariciresinol-4’-*O*-*β*-D-glucopyranoside |  | -6.88961124 | | Swertia Chirayita | (Zhou et al., 2015) |
|  | *Erythro-2-(4-allyl-2,6-dimethoxyphenoxy)-1-(3,4-methylenedioxyphenyl) propan-1-ol acetate* |  | -6.87998247 | | Myristica Fragrans | (S. K. Francis et al., 2019) |
|  | *Erythro*-2-(4-allyl-2,6-dimethoxyphenoxy)-1-(3,4-  dimethoxyphenyl) propan-1-ol acetate |  | -6.87917423 | | Myristica Fragrans | (Morikawa et al., 2016b) |
|  | Swerchirin or Methylbellidifolin |  | -6.87740326 | | Swertia Chirayita | (Ghosal et al., 1973) |
|  | Fragransin C1 |  | -6.87624454 | | Myristica Fragrans | (Hattori et al., 1987) |
|  | Nectandrin A |  | -6.87444782 | | Myristica Fragrans | (Nguyen et al., 2010) |
|  | Fragransin C2 |  | -6.87159348 | | Myristica Fragrans | (Hattori et al., 1987) |
|  | Decussatin |  | -6.86495209 | | Swertia Chirayita | (Ghosal et al., 1973) |
|  | 16a,17-dihydroxy-ent-kauran-19-diglycoside |  | -6.8604S7077 | | Datura Metel | (Liu et al., 2022a) |
|  | Fragransin D3 |  | -6.85784817 | | Myristica Fragrans | (Hada, Hattori, Tezuka, Kikuchi, Phytochemistry, et al., 1988) |
|  | Herpetol B |  | -6.85491085 | | Datura Metel | (B.Y. Yang, H.B. Jiang, Y. Liu, Z.P. Xu, 2018) |
|  | *Meso*-monomethyldihydroguaiaretic acid |  | -6.84500217 | | Myristica Fragrans | (Min et al., 2011) |
|  | Daturmetelide G |  | -6.84448671 | | Datura Metel | (J. Tan, Liu, Cheng, Sun, Pan, Guan, et al., 2020) |
|  | Amarogentin |  | -6.84427834 | | Swertia Chirayita | (Cai et al, 2006) |
|  | Dehydrodieugenol |  | -6.8402195 | | Myristica Fragrans | (Isogai et al., 1973) |
|  | Fragransin D2 |  | | -6.83769464 | Myristica Fragrans | (Hada, Hattori, Tezuka, Kikuchi, Phytochemistry, et al., 1988) |
|  | Fragransol C |  | -6.82912111 | | Myristica Fragrans | (Hattori et al., 1993) |
|  | (E)-methyl 4-(3-(4hydroxyphenyl) -N-methylacrylamido) butanoate |  | -6.82806587 | | Datura Metel | (B. Yang, Xia, Wang, Dou, Fitoterapia, et al., 2010) |
|  | 3-(4-Hydroxy-3-methoxyphenyl)- *N*-[2-(4-hydroxyphenyl)-2- methoxyethyl] acrylamide |  | -6.81954145 | | Datura Metel | (B. Yang, Xia, Wang, Dou, Fitoterapia, et al., 2010) |
|  | Daturmetelide I |  | -6.81939554 | | Datura Metel | (J. Tan, Liu, Cheng, Sun, Pan, Guan, et al., 2020) |
|  | Kaempferol-3-*O*-*β*-D-glucopyranoside |  | -6.81762218 | | Datura Metel | (Andrade et al., 2011) |
|  | Myristic Acid |  | -6.81746483 | | Myristica Fragrans | (Ha et al., 2020) |
|  | (7S,8'R,7'R)-4,4'-dihydroxy-3,3'- dimethoxy-7',9-epoxylignan |  | -6.81299973 | | Myristica Fragrans | (Min et al., 2011) |
|  | Kaempferol-7-*O*-glucoside |  | -6.81234217 | | Datura Metel | (Andrade et al., 2011) |
|  | (8*R*,8′*S*)-7′-(3′,4′-Methylenedioxyphenyl)-8,8′-  dimethyl-7-(3,4-dihydroxyphenyl)-butane |  | -6.80274391 | | Myristica Fragrans | (Ha et al., 2020) |
|  | 6,7-dimethyl-1-D-ribityl-quinoxaline-2,3(1H,4H) - dione-5′ -O-β- D glucopyranoside |  | -6.80199242 | | Datura Metel | (B. Yang, Xia, Wang, Dou, Fitoterapia, et al., 2010) |
|  | (6 R,7 E,9 R）-9-hydroxy-4,7-megastigmadien-3-one 9-O-[α-L-arabin-opyranosyl-（ l→6）-β-D-glucopyranoside] |  | -6.79880428 | | Datura Metel | B. Yang, Yang, et al., 2018) |
|  | Withametelin P |  | -6.79618883 | | Datura Metel | (Pan, Wang, & Hu, 2007) |
|  | 2-dodecycyclobutanone |  | -6.7849431 | | Myristica Fragrans | (Ha et al., 2020) |
|  | Daturmeteside B |  | -6.78187418 | | Datura Metel | (J. Tan, Liu, Cheng, Sun, Liu, et al., 2020) |
|  | Baimantuoluoline M |  | -6.76837492 | | Datura Metel | (Liu et al., 2020) |
|  | 1-Hydroxy-2,3,4,7-tetramethoxy xanthone |  | | -6.76338196 | Swertia Chirayita | (Shi et al., 2004) |
|  | Daturataturin A |  | -6.76226187 | | Datura Metel | (Ma et al., 2006) |
|  | Deacetylcentapicrin |  | -6.75616074 | | Swertia Chirayita | (L. Cai, S. Wang, T. Li, 2006) |
|  | *Erythro-2-(4-allyl-2,6-dimethoxyphenoxy)-1-(4-hydroxy-3,5-dimethoxyphenyl) propan-1-ol* |  | -6.75151777 | | Myristica Fragrans | (HATTORI et al., 1987) |
|  | Stigmasterol |  | -6.75140238 | | Datura Metel | (Alam et al., 2020) |
|  | Raphidecursinol |  | -6.74671412 | | Myristica Fragrans | (Morikawa et al., 2016a) |
|  | Baimantuoluoside G |  | -6.74239111 | | Datura Metel | (B. Yang, Xia, Wang, Dou, research, et al., 2010) |
|  | N-cis-feruloyl  tyramine |  | -6.74041891 | | Datura Metel | (S. Wang et al., 2021) |
|  | Benzyl primeveroside |  | | -6.74011612 | Datura Metel | Tan et al. (2022) |
|  | Baimantuoluoside E |  | -6.73567057 | | Datura Metel | (B.Y. Yang, H.B. Jiang, Y. Liu, Z.P. Xu, 2018) |
|  | *Erythro*-2-(4-allyl-2,6-dimethoxyphenoxy)-1-(3,4-methylenedioxyphenyl)  propan-1-ol |  | -6.73472834 | | Myristica Fragrans | (S. K. Francis et al., 2019) |
|  | Verrucosin |  | -6.73265648 | | Myristica Fragrans | (Duan et al., 2009b) |
|  | Epi-syringaresinol-4’’-*O*-*β*-D-glucopyranoside |  | | -6.72440672 | Swertia Chirayita | (Zhou et al., 2015) |
|  | Myrifralignan E |  | -6.71548986 | | Myristica Fragrans | (G. Cao et al., 2015) |
|  | Machilin D |  | -6.71486568 | | Myristica Fragrans | (Hada, Hattori, Tezuka, Kikuchi, & Namba, 1988) |
|  | Kaempferol-3-*O*-*β*-D-glucopyranoside-7-*O*-*α*-L-rhamnopyranoside |  | -6.71479177 | | Dastura Metel | (Andrade et al., 2011) |
|  | 7*α*,27-Dihydroxy-1-oxowitha- 2,5,24-trienolide |  | -6.70789528 | | Datura Metel | (Ma et al., 2006) |
|  | (+)*-Erythro*-(1*S,*2*R*)-2-(4-allyl-2,6-dimethoxyphenoxy)-  1-(4-hydroxy-3-methoxyphenyl) propan-1-ol |  | -6.70660353 | | Myristica Fragrans | (Morikawa et al., 2016b) |
|  | (*Z*)-3-Hexenyl-*β*-D-glucopyranoside |  | | -6.70250845 | Datura Metel | Tan et al. (2022) |
|  | Quercetin-7-*O*-glucoside |  | -6.70060062 | | Datura Metel | (Mai, Cuc, Anh, Nhiem, …, et al., 2017) |
|  | *Erythro*-2-(4-allyl-2,6-dimethoxyphenoxy)-1-(4-  hydroxy-3-methoxyphenyl) propan-1-ol |  | -6.69825745 | | Myristica Fragrans | (S. K. Francis et al., 2019) |
|  | Swertinin |  | -6.69671202 | | Swertia Chirayita | (Tabassum et al., 2012) |
|  | Baimantuoluoline D |  | -6.69399118 | | Datura Metel | (B. Yang et al., 2008) |
|  | (-)-De-4′ ,4′′ -*O*-dimethylepimagnolin A |  | -6.69297171 | | Datura Metel | (B. Yang, Luo, et al., 2018) |
|  | Swertiachoside B (3-nortetrahydroswertianolin) |  | -6.68315506 | | Swertia Chirayita | (Zhou et al., 2015) |
|  | 8-*O*-[*β*-D-xylopyranosyl-(1→6)-*β*-D-glucopyranosyl]-1,7- dihydroxyl-3-methoxy xanthone |  | -6.6728673 | | Swertia Chirayita | (Zhou et al., 2015) |
|  | 1-*O*-*β*-D-glucopyranosyl-3,5,8-trihydroxy xanthone |  | -6.6691823 | | Swertia Chirayita | (Zhou et al., 2015) |
|  | (−)-(2R)-2-(4-Allyl-2,6-dimethoxyphenoxy)-1-(4-hydroxy-3-methoxyphenyl) propane |  | -6.66828489 | | Myristica Fragrans | (Morikawa et al., 2016b) |
|  | Cleomiscosin A |  | -6.66351175 | | Datura Metel | (B.Y. Yang, H.B. Jiang, Y. Liu, Z.P. Xu, 2018) |
|  | Saucernetindiol |  | -6.65211773 | | Myristica Fragrans | (Nguyen et al., 2010) |
|  | Daturmetesides E |  | -6.64801502 | | Datura Metel | (J. Tan, Liu, Cheng, Sun, Liu, et al., 2020) |
|  | Ethyl Myristate |  | -6.64276934 | | Myristica Fragrans | (Ha et al., 2020) |
|  | 8’-*α*-hydroxyllariciresinol-4-*O*-*β*-D-glucopyranoside |  | -6.63771915 | | Swertia Chirayita | (Zhou et al., 2015) |
|  | Daturmetelide M |  | -6.63261366 | | Datura Metel | (J. Tan, Liu, Cheng, Sun, Pan, Guan, et al., 2020) |
|  | 3,3’,5-trihydroxybiphenyl |  | -6.63242626 | | Swertia Chirayita | (Zhou et al., 2015) |
|  | Fragransin E1  or machilin F |  | -6.62899733 | | Myristica Fragrans | (Hada, Hattori, Tezuka, Kikuchi, Phytochemistry, et al., 1988) |
|  | Fragransin B1 |  | | -6.62882853 | Myristica Fragrans | (Hattori et al., 1987) |
|  | Benzyl-*O*-*α*-L-rhamnopyranosyl-(1 → 6)-*β*-D-glucopyranoside |  | -6.62393856 | | Datura Metel | Tan et al. (2022) |
|  | Surinamensin |  | | -6.62269783 | Myristica Fragrans | (S. Francis et al., 2019) |
|  | *erythro*-  (*7S,8R*)-Δ8′-4,7-dihydroxy-3,5,3′-trimethoxy-8-*O*-4′-neolignan |  | -6.62246704 | | Myristica Fragrans |  |
|  | (+)-erythro-(7S,8R)-∆8′-7-hydroxy-3,4-methylenedioxy3′,5′-dimethoxy-8-O-4′-neolignan |  | -6.61728287 | | Myristica Fragrans | (Isogai et al., 2014) |
|  | Citroside A |  | -6.61203718 | | Datura Metel | (R. Guo, Y. Liu, J. Pan, W. Guan, B.Y. Yang, n.d.) |
|  | Fragransin C1 |  | -6.61201334 | | Myristica Fragrans | (Hattori et al., 1987) |
|  | Maceneolignan G |  | -6.61076164 | | Myristica Fragrans | (G.-Y. Cao et al., 2015) |
|  | Daturmeteside C |  | -6.61061573 | | Datura Metel | (J. Tan, Liu, Cheng, Sun, Liu, et al., 2020) |
|  | *Threo*-2-(4-allyl-2,6-dimethoxyphenoxy)-1-(3,4-dimethoxyphenyl)  propan-1-ol |  | -6.6103673 | | Myristica Fragrans | (HATTORI et al., 1987) |
|  | Daturmeteside A |  | -6.60952568 | | Datura Metel | (J. Tan, Liu, Cheng, Sun, Liu, et al., 2020) |
|  | (7R,8S)-2-(4-propenyl-2-methoxyphenoxy)-1-(3,4,5-trimethoxyphenyl)-propan-1-ol |  | -6.60739231 | | Myristica Fragrans | (Ha et al., 2020) |
|  | 1-Hydroxy-3,5-dimethoxy xanthone |  | -6.60388899 | | Swertia Chirayita | (Shi et al., 2004) |
|  | Beta-Bisabolene |  | -6.60155582 | | Myristica Fragrans | (Trifan et al., 2023) |
|  | (2*S*, *E*)-*N*-[2-hydroxy-2-(4- hydroxyphenyl)-ethyl] ferulamide |  | -6.59721327 | | Datura Metel | (B. Yang, Xia, Wang, Dou, Fitoterapia, et al., 2010) |
|  | 3-methoxyliacrin B |  | -6.59289265 | | Myristica Fragrans | (S. Francis et al., 2019) |
|  | Maceneolignan I |  | -6.5903492 | | Myristica Fragrans | (Morikawa et al., 2018) |
|  | *Erythro-2-(4-allyl-2,6-dimethoxyphenoxy)-1-(5-acetoxy-3,4-dimethoxyphenyl) propan-1-ol acetate* |  | -6.58670425 | | Myristica Fragrans | (Morikawa et al., 2016b) |
|  | Daturafoliside A |  | -6.58668327 | | Datura Metel | (B. Yang, Guo, Li, Wu, et al., 2014) |
|  | Methylswertianin or Swertiaperennin |  | -6.58574963 | | Swertia Chirayita | (You et al., 2017) |
|  | *N*-*trans*-*p*-coumaroyltyramine |  | -6.58250332 | | Datura Metel | (B. Yang, Xia, Wang, Dou, Fitoterapia, et al., 2010) |
|  | *N*-*trans*-feruloyltyramine |  | | -6.57310009 | Datura Metel | (B. Yang, Xia, Wang, Dou, Fitoterapia, et al., 2010) |
|  | Daturmeteside D |  | -6.57072878 | | Datura Metel | (J. Tan, Liu, Cheng, Sun, Liu, et al., 2020) |
|  | Daturmeteside E |  | -6.57072878 | | Datura Metel | (J. Tan, Liu, Cheng, Sun, Liu, et al., 2020) |
|  | Fragransin D1 |  | | -6.560884 | Myristica Fragrans | (Hada, Hattori, Tezuka, Kikuchi, Phytochemistry, et al., 1988) |
|  | Macelignan |  | | -6.56001711 | Myristica Fragrans | (Thuong et al., 2014) |
|  | Oleic Acid |  | | -6.55959034 | Myristica Fragrans | (Ha et al., 2020) |
|  | *Threo*-2-(4-allyl-2,6-dimethoxyphenoxy)-1-(4-hydroxy-  3-methoxyphenyl) propan-1-ol |  | -6.55900812 | | Myristica Fragrans | (Zhang et al., 2015) |
|  | Syringaresinol-4’’-*O*-*β*-D-glucopyranoside |  | -6.55862999 | | Swertia Chirayita | (Zhou et al., 2015) |
|  | Kaempferol-7-*O*-*α*-L-rhamnopyranoside |  | -6.55531883 | | Datura Metel | (Andrade et al., 2011) |
|  | (2S,3S)-2,3-Dihydro-2-(3,4-methylenedioxyphenyl)-7-methoxy-3-methyl-5-(E-propenyl) benzofuran |  | -6.55055428 | | Myristica Fragrans | (Kimura et al., 2010) |
|  | Loganic acid |  | -6.54775333 | | Swertia Chirayita | (Kumar et al., 2015) |
|  | N-trans-ferulyl  tryptamine |  | -6.54705429 | | Datura Metel | (Hwang et al., 2016) |
|  | *α*-Mangostin |  | -6.54702759 | | Swertia Chirayita | (Reyes-Escogido et al., 2011) |
|  | Daturglycoside A |  | -6.53999805 | | Datura Metel | Tan et al. (2022) |
|  | (7S,8′R,7′R)-4,4′-Dihydroxy-3,3′-dimethoxy  7′,9-epoxylignan |  | -6.53270912 | | Myristica Fragrans | (Hattori et al., 1987) |
|  | Maceneolignan J |  | -6.53232956 | | Myristica Fragrans | (Morikawa et al., 2018) |
|  | *Threo-1-(4-hydroxy-3,5-dimethoxyphenyl)-2-(2-methoxy-4-(1(E)-propenyl) phenoxy) propan-1-ol* |  | -6.53228045 | | Myristica Fragrans | (Hada, Hattori, Tezuka, Kikuchi, & Namba, 1988) |
|  | Daturametelin A |  | -6.52938128 | | Datura Metel | (Bellila et al., 2011) |
|  | Maceneolignan E |  | -6.52617216 | | Myristica Fragrans | (Morikawa et al., 2016a) |
|  | Myristicanol B |  | -6.52554321 | | Myristica Fragrans | (Hada, Hattori, Tezuka, Kikuchi, & Namba, 1988) |
|  | Acuminatin |  | -6.52541828 | | Myristica Fragrans | (K. Francis et al., 2014) |
|  | *Threo-2-(4-allyl-2,6-dimethoxyphenoxy)-1-(4-hydroxy-3-methoxyphenyl) propan-1-ol methyl ether* |  | -6.52150345 | | Myristica Fragrans | (HATTORI et al., 1987) |
|  | (+)-erythro-(7S,8R)-∆8′ - 7-hydroxy-3,4,5,3′,5′-pentamethoxy-8-O-4′-neolignan |  | -6.51476145 | | Myristica Fragrans | (Isogai et al., 2014) |
|  | Fragransin C3b |  | -6.50658703 | | Myristica Fragrans | (Hattori et al., 1987) |
|  | Daturametelin J |  | -6.50007153 | | Datura Metel | (B.Y. Yang, H.B. Jiang, Y. Liu, Z.P. Xu, 2018) |
|  | Baimantuoluoside F |  | -6.49907255 | | Datura Metel | (B. Yang, Xia, Wang, Dou, research, et al., 2010) |
|  | Fragransin C3a |  | -6.49418736 | | Myristica Fragrans | (Hattori et al., 1987) |
|  | *Threo-1-(4-hydroxy-3-methoxyphenyl)-1-methoxy 2-(2-methoxy-4-(1(E)-propenyl) phenoxy) propane* |  | | -6.49399614 | Myristica Fragrans | (Hada, Hattori, Tezuka, Kikuchi, & Namba, 1988) |
|  | Licarin A |  | -6.49115992 | | Myristica Fragrans | (S. Francis et al., 2019) |
|  | Daturameteline F |  | -6.48713255 | | Datura Metel | (B. Y. Yang, 2005) |
|  | Isolicarin A |  | -6.48602867 | | Myristica Fragrans | (Li et al., 2007) |
|  | (+)-Erythro-(1S,2R)-2-(4-allyl-2,6-dimethoxyphenoxy)-1-(3,4-methylenedioxyphenyl) propan-1-ol |  | -6.484272 | | Myristica Fragrans | (Morikawa et al., 2016b) |
|  | Baimantuoluoline R |  | -6.48196936 | | Datura Metel | (Liu et al., 2020) |
|  | Kaempferol-7-*O*-*β*-D-glucopyranoside |  | -6.48182631 | | Datura Metel | (Z.Y. Li, H.X. Kuang, Y.G. Xia, 2010) |
|  | Daturameteloside J |  | | -6.47990036 | Datura Metel | (B. Y. Yang, 2005) |
|  | Daturmetelide E |  | -6.47935867 | | Datura Metel | (J. Tan, Liu, Cheng, Sun, Pan, Guan, et al., 2020) |
|  | *β*-Sitosterol-*β*-D-glucoside |  | -6.47542715 | | Swertia Chirayita | (Pant et al., 2002) |
|  | 1,10-seco-withametelin B |  | -6.47260952 | | Datura Metel | (B. Yang, Xia, Wang, Dou, Fitoterapia, et al., 2010) |
|  | Daturmetelide L |  | -6.46980047 | | Datura Metel | (J. Tan, Liu, Cheng, Sun, Pan, Guan, et al., 2020) |
|  | Isomangiferin |  | -6.46492434 | | Swertia Chirayita | (He et al., 2017) |
|  | (7S,8′R,7′R)-4,4′-Dihydroxy-3,3′-dimethoxy-  7′,9-epoxylignan |  | -6.4640789 | | Myristica Fragrans | (Min et al., 2011) |
|  | (7R, 8R)-7,8-Dihydro-7-(3,4-dihydrophenyl)-3’-methoxy-8-methyl-1’-(E-propenyl) Benzofuran |  | -6.46075249 | | Myristica Fragrans | (Ha et al., 2020) |
|  | Phenowithanolide |  | -6.46053648 | | Datura Metel | (Bellila et al., 2011) |
|  | Dmetelisproside A |  | -6.4601965 | | Datura Metel | (Aberham et al., 2010) |
|  | *N-trans*-feruloyl-3′ ,4′ - dihydroxyphenylethylamine |  | -6.45371771 | | Datura Metel | (B. Yang, Xia, Wang, Dou, Fitoterapia, et al., 2010) |
|  | Phenethanol-*β*-vicianoside |  | -6.45151949 | | Datura Metel | Tan et al. (2022) |
|  | Fragransol D |  | -6.44559908 | | Myristica Fragrans | (HATTORI et al., 1988) |
|  | Daturameteline G |  | -6.44333076 | | Datura Metel | (B. Y. Yang, 2005) |
|  | 1-Hydroxy-3,7-dimethoxy xanthone |  | | -6.44199753 | Swertia Chirayita | (You et al., 2017) |
|  | 12β -hydroxy-1,10-seco-withametelin B |  | -6.43587589 | | Datura Metel | (B. Yang, Xia, Wang, Dou, Fitoterapia, et al., 2010) |
|  | Coniferin |  | -6.43420696 | | Datura Metel | (B. Yang, Yang, et al., 2018) |
|  | Withametelin F |  | -6.4338665 | | Datura Metel | (Manickam et al., 1993) |
|  | 7-(4-Hydroxy-3-methoxyphenyl)-7-(3,4-  methylenedioxyphenyl)-8,8-lignan-7-methyl  ether |  | -6.43036509 | | Myristica Fragrans | (Ha et al., 2020) |
|  | Daturglycoside F |  | -6.42676401 | | Datura Metel | (Flannery, B.~P.Teukolsky et al., 2004) |
|  | Myrifralignan A |  | -6.42390966 | | Myristica Fragrans | (G. Cao et al., 2015) |
|  | Daturmetesides D |  | -6.4210391 | | Datura Metel | (J. Tan, Liu, Cheng, Sun, Liu, et al., 2020) |
|  | Myrislignanometin E |  | -6.419168 | | Myristica Fragrans | (Hada, Hattori, Tezuka, Kikuchi, & Namba, 1988) |
|  | Daturilin |  | -6.41475105 | | Datura Metel | (Siddiqui et al., n.d.) |
|  | Fragransin B3 |  | -6.41218233 | | Myristica Fragrans | (Hattori et al., 1987) |
|  | Aurantiamide |  | -6.40755844 | | Datura Metel | (B.Y. Yang, Y.M. Luo, Y. Liu, X. Yin, Y.Y. Zhou, 2018) |
|  | Odoratisol A [3′-methoxy-licarin A] |  | -6.40648031 | | Myristica Fragrans | (S. Francis et al., 2019) |
|  | cycloartenol |  | -6.4043045 | | Myristica Fragrans | (Hou et al., 2012) |
|  | Monomethyl Dihydroguaiaretic acids |  | -6.40071726 | | Myristica Fragrans |  |
|  | 3-(4-Allyl-2,6-dimethoxy-phenyloxy)-2-methyl-5-methoxy-  2,3-dihydrobenzofuran |  | -6.39844036 | | Myristica Fragrans | (Chiu et al., 2016a) |
|  | (2*S*,3*S*)-2,3-Dihydro-2-(5-methoxy-  3,4-methylenedioxyphenyl)-7-methoxy-3-methyl-5-(*E*propenyl)  benzofuran |  | -6.39570141 | | Myristica Fragrans | (Kimura et al., 2010) |
|  | Daturaterpenoid C |  | -6.38672543 | | Datura Metel | (Liu et al., 2019) |
|  | Liacrin B |  | -6.38629866 | | Myristica Fragrans | (S. Francis et al., 2019) |
|  | (+)-(7*S*,8*S*)-4-hydroxy-3,3′ ,5′ - Trimethoxy-8′ ,9′ -dinor-8,4′ - oxyneolignan-7,9-diol-7′ -oic acid |  | -6.38448095 | | Datura Metel | (B.Y. Yang, C.L. Yang, Y. Liu, 2017) |
|  | Licarin B |  | -6.383811 | | Myristica Fragrans | (S. Francis et al., 2019) |
|  | (2*S*,3*S*)-2,3-Dihydro-2-(4-hydroxy-3,5-  dimethoxyphenyl)-7-methoxy-3-methyl-5-(*E*-propenyl)  benzofuran |  | -6.37888384 | | Myristica Fragrans | (Kimura et al., 2010) |
|  | Withametelin O |  | -6.37609673 | | Datura Metel | (Pan, Wang, & Hu, 2007) |
|  | (8*R*,8′*S*)-7-(3,4-Methylenedioxyphenyl)-  8-methyl-8′-hydroxymethyl-7′-(3′,4′-  methylenedioxyphenyl)-butanol |  | -6.37326527 | | Myristica Fragrans | (Ha et al., 2020) |
|  | *N*-*cis*-feruloyloctopamine |  | -6.37273026 | | Datura Metel | (B.Y. Yang, Y.M. Luo, Y. Liu, X. Yin, Y.Y. Zhou, 2018) |
|  | Daturamalakin B |  | -6.369102 | | Datura Metel | (Bellila et al., 2011) |
|  | Baimantuoluoline I |  | -6.36018991 | | Datura Metel | (B.-Y. Yang et al., 2013) |
|  | Fragransin B2 |  | | -6.35195255 | Myristica Fragrans | (Hattori et al., 1987) |
|  | 1-Hydroxy-3,5,8-trimethoxy xanthone |  | -6.35142326 | | Swertia Chirayita | (Ghosal et al., 1973) |
|  | odoratisol A |  | -6.35065842 | | Myristica Fragrans | (Duan et al., 2009b) |
|  | (−)-(8R)-∆8′-4-hydroxy-3,3′,5′-trimethoxy-8-O-4′-neolignan |  | -6.34934092 | | Myristica Fragrans | (Isogai et al., 2014) |
|  | *Erythro-1-(4-hydroxy-3-methoxyphenyl)-1-methoxy-2-(2-methoxy-4-(1(E)-propenyl) phenoxy) propane* |  | -6.34074211 | | Myristica Fragrans | (Hada, Hattori, Tezuka, Kikuchi, & Namba, 1988) |
|  | Otobaphenol |  | -6.3350749 | | Myristica Fragrans | (S. Yang et al., 2006) |
|  | (-)-Syringaresinol |  | -6.33390093 | | Datura Metel | (B. Yang et al., 2007) |
|  | *Erythro*-2-(4-allyl-2,6-dimethoxyphenoxy)-1-(3,4-  dimethoxyphenyl) propan-1-ol |  | -6.32100296 | | Myristica Fragrans | (Adjene & Igbigbi, 2010) |
|  | Baimantuoluoline N |  | -6.31605864 | | Datura Metel | (Liu et al., 2020) |
|  | Dmetelin D |  | | -6.30954742 | Datura Metel | (B. Yang, Guo, Li, Liu, et al., 2014) |
|  | Mangiferin |  | -6.30662823 | | Swertia Chirayita | (Ghosal et al., 1973) |
|  | 1,3,8-Trihydroxy-5-methoxy xanthone |  | -6.30662823 | | Swertia Chirayita | (Ghosal et al., 1973) |
|  | Daturglycoside B |  | -6.28520203 | | Datura Metel | Tan et al. (2022) |
|  | Celerioside E |  | -6.28412676 | | Datura Metel | (Liu et al., 2022a) |
|  | Grandisin |  | -6.28348398 | | Myristica Fragrans | (S. Francis et al., 2019) |
|  | Licarin E |  | -6.28228951 | | Myristica Fragrans | (Kapoor et al., 2013) |
|  | 3′-Methoxy-licarin B |  | -6.28209019 | | Myristica Fragrans | (Morikawa et al., 2016a) |
|  | Daturanolide A |  | -6.28105736 | | Datura Metel | (X.-Y. Wang et al., 2019) |
|  | 3”- hydroxydemethyldactyloidin. |  | -6.27982378 | | Myristica Fragrans |  |
|  | (−)-Miliusfragranol B |  | -6.27251196 | | Myristica Fragrans | (Morikawa et al., 2018) |
|  | Baimantuoluoline X |  | -6.27124548 | | Datura Metel | (Liu et al., 2020) |
|  | 4-(4-Hydroxyphenyl)-2- butanone 4′ -*O*-*β*-D-glucopyranoside |  | -6.26980305 | | Datura Metel | Tan et al. (2022) |
|  | *Erythro-2-(4-allyl-2,6-dimethoxyphenoxy)-1-(4-hydroxy-3-methoxyphenyl) propan-1-ol methyl ether* |  | -6.2657876 | | Myristica Fragrans | (HATTORI et al., 1987) |
|  | 2-(3,4-dimethyl-2,5-dihydro-1H-pyrrol-2-yl) -1- methylethyl pentanoate |  | -6.26273394 | | Datura Metel | (J. Tan, Liu, Cheng, Sun, Liu, et al., 2020) |
|  | Withametelins N |  | -6.25849533 | | Datura Metel | (Pan, Wang, products, et al., 2007) |
|  | Daturmetelide J |  | -6.25720024 | | Datura Metel | (J. Tan, Liu, Cheng, Sun, Pan, Guan, et al., 2020) |
|  | Citronellyl Acetate |  | -6.24696159 | | Myristica Fragrans | (Ha et al., 2020) |
|  | Myticaganal C |  | -6.24446535 | | Myristica Fragrans | (Chumkaew & Srisawat, 2019) |
|  | Chiratol |  | -6.24387741 | | Swertia Chirayita | (Tabassum et al., 2012) |
|  | (6*R*,9*R*)-3-Oxo-*α*-ionol-9-*O*-*β*-D-glucopyranoside |  | -6.23709202 | | Datura Metel | (Liu et al., 2019) |
|  | Withametelin L |  | -6.23462629 | | Datura Metel | (Pan, Wang, & Hu, 2007) |
|  | Baimantuoluoline S |  | -6.23008966 | | Datura Metel | (Liu et al., 2020) |
|  | Myrisisolignan |  | -6.22096491 | | Myristica Fragrans | (HATTORI et al., 1986) |
|  | 5-(6,7-Dimethoxy-3-methyl-  5-propenyl-2,3-dihydrobenzofuran-  2-yl)-3-  methoxy-benzene-1,2-diol |  | -6.21404076 | | Myristica Fragrans | (Ha et al., 2020) |
|  | 2-Phenylethyl-*β*-D-glucopyranoside |  | -6.21040344 | | Datura Metel | Tan et al. (2022) |
|  | Isodihydrocarinatidin |  | -6.20844412 | | Myristica Fragrans | (G.-Y. Cao et al., 2013) |
|  | (2S,3S)-2-(4-Hydroxy-3-methoxyphenyl)-5-formyl-7-methoxy-3-methyldihydrobenzofuran |  | -6.20739365 | | Myristica Fragrans | (Morikawa et al., 2016a) |
|  | Withametelins I |  | | -6.20405245 | Datura Metel | (Pan, Wang, products, et al., 2007) |
|  | Meso-dihydroguaiaretic acid |  | -6.20300627 | | Myristica Fragrans | (Lee et al., 2009) |
|  | Maceneolignan B |  | -6.20125008 | | Myristica Fragrans | (Morikawa et al., 2016a) |
|  | (6*R*,9*R*)-3-One-*α*-Ionol-9-*O*- *β*-D-Glucopyranoside |  | -6.19962215 | | Datura Metel | (H. X. Kuang et al., 2008) |
|  | Iriflophenone |  | -6.19339371 | | Swertia Chirayita | (Kumar et al., 2015) |
|  | Withametelins M |  | -6.18452883 | | Datura Metel | (Pan, Wang, products, et al., 2007) |
|  | Baimantuoluoamide A |  | -6.18389463 | | Datura Metel | (B. Yang, Xia, Wang, Dou, Fitoterapia, et al., 2010) |
|  | Baimantuoluoline W |  | -6.17800999 | | Datura Metel | (Liu et al., 2020) |
|  | *Erythro-1-(4-hydroxy-3-methoxyphenyl)-2-(2-methoxy-4-(1(E)-propenyl) phenoxy) propan-1-ol* |  | -6.17525387 | | Myristica Fragrans | (Hada, Hattori, Tezuka, Kikuchi, & Namba, 1988) |
|  | Swertianin |  | | -6.17212725 | Swertia Chirayita | (Mandal & Chatterjee, 1987) |
|  | 2,6-Bis(1-phenylethyl) phenol |  | | -6.16636372 | Datura Metel | (Mohiuddin et al., 2018) |
|  | Daturglycoside D |  | -6.16393328 | | Datura Metel | Tan et al. (2022) |
|  | Daturameteline A |  | -6.163836 | | Datura Metel | (B. Y. Yang, 2005) |
|  | 5-((2R,3S)-4-(4-hydroxy-3-methoxyphenyl)-3-  methylbutan-2-yl)-3-methoxybenzene-1,2-diol |  | -6.16251135 | | Myristica Fragrans | (Ha et al., 2020) |
|  | Daturmetelide K |  | -6.15932846 | | Datura Metel | (J. Tan, Liu, Cheng, Sun, Pan, Guan, et al., 2020) |
|  | (7S)-9-(4′-Hydroxy-3′-methoxyphenyl)-7-hydroxypropyl)benzene-2,4-diol |  | -6.1578536 | | Myristica Fragrans | (Ha et al., 2020) |
|  | Skimmianine |  | -6.15764856 | | Datura Metel | (B. Y. Yang, 2005) |
|  | Kaurane acid glycoside B |  | -6.1562295 | | Datura Metel | (B. you Yang et al., 2018) |
|  | (+)-Guaiacin |  | -6.15608311 | | Myristica Fragrans | (Min et al., 2011) |
|  | Licarin C |  | -6.15552568 | | Myristica Fragrans | (Chiu et al., 2016b) |
|  | 1,8-Dihydroxy-3,5,7-trimethoxy xanthone |  | -6.15535927 | | Swertia Chirayita | (You et al., 2017) |
|  | Baimantuoluoline U |  | -6.15323591 | | Datura Metel | (Liu et al., 2020) |
|  | Geranyl Acetate |  | -6.15147734 | | Myristica Fragrans | (Trifan et al., 2023) |
|  | 2-decyccyclobutanone |  | -6.14950037 | | Myristica Fragrans | (Ha et al., 2020) |
|  | Syringaresinol |  | -6.14132118 | | Swertia Chirayita | (Chakravarty et al., 1994) |
|  | (7R,8R)-7,8-Dihydro-7-(3,4-dihydroxyphenyl)-3′-methoxy-8- methyl-1′-(E-propenyl)benzofuran |  | -6.14130592S | | Myristica Fragrans | (Duan et al., 2009a) |
|  | Fragransol A |  | -6.13850546 | | Myristica Fragrans | (H. Gan, J. He, X.Y. Wang, Y.W. Wang, H.M. Gao, H. Jiang, W.K. Zhang, 2021) |
|  | *N*-[2-(3,4-dihydroxyphenyl-2- hydroxyethyl)]-3-(4- methoxyphenyl)-prop-2-enamide |  | | -6.13571453 | Datura Metel | (B. Yang, Xia, Wang, Dou, Fitoterapia, et al., 2010) |
|  | Withametelin K |  | -6.13199997 | | Datura Metel | (Pan, Wang, & Hu, 2007) |
|  | Congmuyaglyeoside I |  | -6.12725592 | | Datura Metel | (Alam et al., 2020) |
|  | (2*R*,3*R*)-2,3-Dihydro-2-(3,4-dihydroxyphenyl)-7-methoxy-  3-methyl-5-(*E*-propenyl) benzofuran |  | -6.12554598 | | Myristica Fragrans | (Duan et al., 2009b) |
|  | Baimantuoluoline Q |  | -6.12201357 | | Datura Metel | (Liu et al., 2020) |
|  | Maceneolignan D |  | -6.11917639 | | Myristica Fragrans | (Morikawa et al., 2016a) |
|  | Galbacin |  | -6.11778355 | | Myristica Fragrans | (Nguyen et al., 2010) |
|  | Tetrahydrofuroguaiacin B |  | -6.11369467 | | Myristica Fragrans | (Nguyen et al., 2010) |
|  | Baimantuoluoside D |  | -6.09957027 | | Datura Metel | (B. Yang, Xia, Wang, Dou, research, et al., 2010) |
|  | Daturmetelide W |  | -6.09629679 | | Datura Metel | (J. Tan, Liu, Cheng, Sun, Pan, Guan, et al., 2020) |
|  | Foliachinenoside I |  | -6.08864641 | | Datura Metel | (B. Y. Yang et al., 2014) |
|  | Maclurin |  | -6.08415556 | | Swertia Chirayita | (Kumar et al., 2015) |
|  | Baimantuoluoside A |  | -6.08379316 | | Datura Metel | (H. Kuang et al., 2009) |
|  | Anisodamine |  | -6.08358669 | | Datura Metel | (Z.Y. Li, H.X. Kuang, Y.G. Xia, 2010) |
|  | Daturmeteside B |  | -6.07913637 | | Datura Metel | (J. Tan, Liu, Cheng, Sun, Liu, et al., 2020) |
|  | Dmetelin A (terpenoid) |  | -6.07652426 | | Datura Metel | (J. Tan, Liu, Cheng, Sun, Pan, Fitoterapia, et al., 2020) |
|  | Withametelin M |  | -6.07516384 | | Datura Metel | (Pan, Wang, & Hu, 2007) |
|  | Machilin F |  | -6.06307411 | | Myristica Fragrans | (Lee et al., 2009) |
|  | *n*-Butyl-*O*-*α*-D-fructofuranosidase |  | | -6.0554738 | Datura Metel | (Alam et al., 2020) |
|  | Fragransin A2 |  | -6.0469079 | | Myristica Fragrans | (Thuong et al., 2014) |
|  | Austrobailignan-7 |  | -6.04583645 | | Myristica Fragrans | (Hada, Hattori, Tezuka, Kikuchi, Phytochemistry, et al., 1988) |
|  | 1,3,7-Trihydroxy-8-methoxy xanthone |  | -6.0412178 | | Swertia Chirayita | (Shi et al., 2013) |
|  | Salidroside |  | -6.04082108 | | Datura Metel | B. Yang, Yang, et al., 2018) |
|  | Virolane |  | -6.02774477 | | Myristica Fragrans | (Ha et al., 2020) |
|  | Neolancerin |  | -6.02382803 | | Swertia Chirayita | (Zhou et al., 2015) |
|  | Isobellidifolin |  | -6.01535988 | | Swertia Chirayita | (Singh et al., 2012) |
|  | 1,2,8-Trihydroxy-6-methoxy xanthone |  | -6.01217842 | | Swertia Chirayita | (Reyes-Escogido et al., 2011) |
|  | Myrisfrageal B |  | -6.01148319 | | Myristica Fragrans | (G.-Y. Cao et al., 2013) |
|  | Machilin A |  | -6.01032305 | | Myristica Fragrans | (Lee et al., 2009) |
|  | Daturamalakoside B |  | | -6.00396729 | Datura Metel | (Bellila et al., 2011) |
|  | 5,7-diacetyl chrysin |  | -6.0031271 | | Myristica Fragrans | (Ha et al., 2020) |
|  | Hydroxypropyl |  | -6.00285435 | | Myristica Fragrans | (Ha et al., 2020) |
|  | Juglanoside B |  | -6.00220871 | | Datura Metel | (J. Y. Tan et al., 2021) |
|  | Fragransol B |  | -6.00124264 | | Myristica Fragrans | (H. Gan, J. He, X.Y. Wang, Y.W. Wang, H.M. Gao, H. Jiang, W.K. Zhang, 2021) |
|  | 1,8-Dihydroxy-3,7-dimethoxy xanthone |  | -5.99884462 | | Swertia Chirayita | (Ghosal et al., 1973) |
|  | *Threo*-2-(4-allyl-2-methoxyphenoxy)-1-(4-hydroxy-3-  methoxyphenyl) propan-1-ol |  | -5.99863148 | | Myristica Fragrans | (Hada, Hattori, Tezuka, Kikuchi, & Namba, 1988) |
|  | (2*S*,3*S*)-2,3-Dihydro-2-(4-hydroy-3,5-dimethoxyphenyl)-  7-methoxy-3-methyl-5-(*E*-propenyl) benzofuran |  | -5.99764299 | | Myristica Fragrans | (Kimura et al., 2010) |
|  | Withametelins L |  | -5.98890209 | | Datura Metel | (Pan, Wang, products, et al., 2007) |
|  | Daturglycoside E |  | -5.98888206 | | Datura Metel | Tan et al. (2022) |
|  | Vomifoliol |  | -5.98577309 | | Datura Metel | (H. X. Kuang et al., 2008) |
|  | Cuminyl-glucopyranoside |  | -5.98351479 | | Datura Metel | (J. Tan, Liu, Cheng, Sun, Pan, Fitoterapia, et al., 2020) |
|  | 3,4’-dihydroxy-3’-methoxypropiophenone-3-*O*-*β*-D-glucopyranoside |  | -5.98324728 | | Swertia Chirayita | (Zhou et al., 2015) |
|  | Daturmetelide C |  | -5.98271942 | | Datura Metel | (J. Tan, Liu, Cheng, Sun, Pan, Guan, et al., 2020) |
|  | Myticaganal A |  | -5.97856045 | | Myristica Fragrans | (G.-Y. Cao et al., 2013) |
|  | Baimantuoluoline V |  | -5.97479343 | | Datura Metel | (Liu et al., 2020) |
|  | Adenosine |  | -5.97368002 | | Datura Metel | (Mai, Cuc, Anh, Nhiem, Steroids, et al., 2017) |
|  | Myticaganal B |  | -5.97262716 | | Myristica Fragrans | (Chumkaew & Srisawat, 2019) |
|  | Daturamalakin A |  | -5.96331978 | | Datura Metel | (Bellila et al., 2011) |
|  | *trans*-*p-*Hydroxyphenyleth-anol-*p*- *β*-coumarate |  | -5.95915747 | | Datura Metel | (B.Y. Yang, C.L. Yang, Y. Liu, 2017) |
|  | Daturmetelide B |  | -5.95177364 | | Datura Metel | (J. Tan, Liu, Cheng, Sun, Pan, Guan, et al., 2020) |
|  | Withametelin I |  | -5.94164324 | | Datura Metel | (Pan, Wang, & Hu, 2007) |
|  | (2R)-3-(3,4,5-Trimethoxyphenyl)-1,2-propanediol |  | -5.93399954 | | Myristica Fragrans | (Duan et al., 2009b) |
|  | elemicin |  | -5.91596508 | | Myristica Fragrans |  |
|  | 16*β*,17-Dihydroxy-ent-kauran- 19-oic acid |  | -5.91304016 | | Datura Metel | (B. you Yang et al., 2018) |
|  | Nectandrin B |  | -5.91295433 | | Myristica Fragrans | (Nguyen et al., 2010) |
|  | Dmetelin I |  | -5.8970437 | | Datura Metel | (J. Tan, Liu, Cheng, Sun, Pan, Fitoterapia, et al., 2020) |
|  | Myrisfrageal A |  | -5.89695454 | | Myristica Fragrans | (Chumkaew & Srisawat, 2019) |
|  | Withanoside III |  | | -5.89604473 | Datura Metel | (Liu et al., 2020) |
|  | 2,3-Dimethyl-1,4-bis-(3,4-methylenedioxyphenyl)  butan-1-ol |  | -5.88861465 | | Myristica Fragrans | (Ha et al., 2020) |
|  | Withametelins O |  | -5.88641024 | | Datura Metel | (Pan, Wang, products, et al., 2007) |
|  | (6*S*,9*R*)-Roseoside |  | | -5.88506603 | Datura Metel | (Flannery, B.~P.Teukolsky et al., 2004) |
|  | 4-*O*-Methylhonokiol |  | | -5.88037968 | Datura Metel | (B.Y. Yang, C.L. Yang, Y. Liu, 2017) |
|  | Methyl (3*β*)-3-hydroxyolean-12-en-28-oate |  | | -5.87950611 | Swertia Chirayita | (Shi et al., 2013) |
|  | Isolariciresinol |  | -5.87930298 | | Datura Metel | (B.Y. Yang, C.L. Yang, Y. Liu, 2017) |
|  | **20** Daturameteline L |  | -5.87490273 | | Datura Metel | (Tang, 2006) |
|  | *N*-*cis*-*p*-coumaroyloctopamine |  | -5.86951923 | | Datura Metel | (B.Y. Yang, Y.M. Luo, Y. Liu, X. Yin, Y.Y. Zhou, 2018) |
|  | Methyl (3*β*)-3-hydroxyolean-12,15-dien-28-oate |  | -5.86305952 | | Swertia Chirayita | (Shi et al., 2013) |
|  | Baimantuoluoside J |  | -5.86242962 | | Datura Metel | (B.-Y. Yang et al., 2013) |
|  | Withametelin B |  | -5.85606813 | | Datura Metel | (Gupta, Bagchi, & Ray, 1991) |
|  | (S)-4-(1-hydroxy-3-(4-hydroxy-3-methoxyphenyl)  propyl)benzene-1,3-diol |  | -5.85339308 | | Myristica Fragrans | (Ha et al., 2020) |
|  | Kaempferol |  | | -5.84899187 | Datura Metel | (Andrade et al., 2011) |
|  | (8R,9S)-7-(4-Hydroxy-3-methoxyphenyl)-8′-methylbutan-8-yl)-3′-methoxybenzene-4′,5′-diol |  | -5.84639215 | | Myristica Fragrans | (Ha et al., 2020) |
|  | 1-Deoxycarinatone |  | -5.8432312 | | Myristica Fragrans | (Li & Yang, 2007) |
|  | Deoxyloganic acid |  | -5.84083271 | | Swertia Chirayita | (Kumar et al., 2015) |
|  | Daturaterpenoid B |  | -5.83681679 | | Datura Metel | (Liu et al., 2019) |
|  | Withametelins K |  | -5.83345318 | | Datura Metel | (Pan, Wang, products, et al., 2007) |
|  | Dmetelin E |  | -5.82259274 | | Datura Metel | (J. Tan, Liu, Cheng, Sun, Pan, Fitoterapia, et al., 2020) |
|  | 1,10-Seco-withametelin B |  | -5.82096672 | | Datura Metel | (Pan, Wang, & Hu, 2007) |
|  | Aristopyridinone A |  | -5.82009935 | | Datura Metel | (X.-Y. Wang et al., 2019) |
|  | (7S,8S,7′R,8′S)-4,5′-dihydroxy3,3′-dimethoxy-7,7′-epoxylignan |  | -5.80311632 | | Myristica Fragrans | (Ha et al., 2020) |
|  | Staphylionoside D |  | -5.79740334 | | Datura Metel | (R. Guo, Y. Liu, J. Pan, W. Guan, B.Y. Yang, n.d.) |
|  | Withastramonolide |  | -5.79063606 | | Datura Metel | (H. Kuang et al., 2009) |
|  | Alpha-humulene |  | -5.78526735 | | Myristica Fragrans | (Trifan et al., 2023) |
|  | (S)-4-(3-(benzo[d][1,3]  dioxol-5-yl)-1-hydroxypropyl)benzene-1,3-diol |  | -5.78287601 | | Myristica Fragrans | (Ha et al., 2020) |
|  | *N*-benzoyl-L-phenylalaninol |  | -5.78257704 | | Datura Metel | (B.Y. Yang, Y.M. Luo, Y. Liu, X. Yin, Y.Y. Zhou, 2018) |
|  | Daturanolide B |  | -5.78139496 | | Datura Metel | (X.-Y. Wang et al., 2019) |
|  | Daturametelindole C |  | -5.77693081 | | Datura Metel | (Liu et al., n.d.) |
|  | Withametelins J |  | -5.77447701 | | Datura Metel | (Pan, Wang, products, et al., 2007) |
|  | Withametelin H |  | -5.77159452 | | Datura Metel | (M. Manickam, S. Kumar, A. Sinha-Bagchi, S. SinhaI, 1995) |
|  | Cyclo (Phe-Tyr) |  | -5.76551867 | | Datura Metel | (B. Yang, Xia, Wang, Dou, Fitoterapia, et al., 2010) |
|  | Homatropine |  | -5.76229715 | | Datura Metel | (Temerdashev et al., 2012) |
|  | Desmethylbellidifolin |  | -5.76044083 | | Swertia Chirayita | (Ghosal et al., 1973) |
|  | Withametelin N |  | -5.75886536 | | Datura Metel | (Pan, Wang, & Hu, 2007) |
|  | (2R)-3-(3,4,5-trimethoxyphenyl)-1,2-propanediol |  | -5.75766754 | | Myristica Fragrans | (Duan et al., 2009a) |
|  | Alpha-Amorphene |  | -5.7502985 | | Myristica Fragrans | (Ha et al., 2020) |
|  | *p*-Hydroxybenzoic acid-*p*-hydroxyphenylethanol ester |  | -5.74922895 | | Datura Metel | (B. Y. Yang, 2005) |
|  | 6’-*O*-*β*-D-glucopyranosyl spicroside |  | -5.74508429 | | Swertia Chirayita | (Zhou et al., 2015) |
|  | Trans-a-Bergamotene |  | -5.74426174 | | Myristica Fragrans | (Trifan et al., 2023) |
|  | *N*-*trans*-*p*-coumaroyloctopamine |  | | -5.74309778 | Datura Metel | (B.Y. Yang, Y.M. Luo, Y. Liu, X. Yin, Y.Y. Zhou, 2018) |
|  | Balanophonin B |  | -5.74156618 | | Datura Metel | (B. Yang, Luo, et al., 2018) |
|  | 1,3,7,8-Tetrahydroxy xanthone |  | -5.73524427 | | Swertia Chirayita | (Ghosal et al., 1973) |
|  | Bellidifolin |  | -5.73252344 | | Swertia Chirayita | (Ghosal et al., 1973) |
|  | Androsin |  | | -5.70693207 | Datura Metel | (B.Y. Yang, C.L. Yang, Y. Liu, 2017) |
|  | Dinoxin B aglycone |  | -5.70656395 | | Datura Metel | (Vermillion et al., 2011) |
|  | Swertiamarin |  | -5.70240498 | | Swertia Chirayita | (Bhandari et al., 2006) |
|  | *Threo*-austrobailignan-5 |  | -5.69213772 | | Myristica Fragrans | (Kwon et al., 2008) |
| 1. s | swerchirin |  | -5.69141054 | | Swertia Chirayta | (Bajpai et al., 1991) |
|  | Gentiopicroside |  | -5.69077444 | | Swertia Chirayita | (L. Cai, S. Wang, T. Li, 2006) |
|  | Norswertianin |  | -5.68312979 | | Swertia Chirayita | (Reyes-Escogido et al., 2011) |
|  | 3*β*-Tigloyloxytropane |  | -5.6765995 | | Datura Metel | (Doncheva et al., 2006) |
|  | Anoectochine |  | -5.6724329 | | Datura Metel | (B.Y. Yang, H.B. Jiang, Y. Liu, Z.P. Xu, 2018) |
|  | *N*-*cis*-*p*-coumaroyltyramine |  | -5.67119598 | | Datura Metel | (B.Y. Yang, Y.M. Luo, Y. Liu, X. Yin, Y.Y. Zhou, 2018) |
|  | Delta-Elemene |  | -5.66717672 | | Myristica Fragrans | (Trifan et al., 2023) |
|  | *erythro*-2,3-Bis-(4-hydroxy-3- methoxypheyl)-3-methoxy-propanol |  | -5.66490078 | | Datura Metel | (B.Y. Yang, C.L. Yang, Y. Liu, 2017) |
| 1. s | Blumenol A |  | -5.66473818 | | Datura Metel | (H. X. Kuang et al., 2008) |
|  | *Erythro-2-(4-allyl-2-methoxyphenoxy)-1-(4-hydroxy-3-methoxyphenyl) propan-1-ol* |  | -5.66228104 | | Myristica Fragrans | (Hada, Hattori, Tezuka, Kikuchi, & Namba, 1988) |
|  | Isoelemicin |  | -5.64744425 | | Myristica Fragrans | (Al-Qahtani et al., 2022) |
|  | Daturmetelide D |  | -5.62809801 | | Datura Metel | (J. Tan, Liu, Cheng, Sun, Pan, Guan, et al., 2020) |
|  | N-butyl-O-β-D-fructofuranoside |  | -5.62604046 | | Datura Metel | B. Yang, Yang, et al., 2018) |
|  | 4-epi-hederagenin |  | -5.62578297 | | Swertia Chirayita | (Chen, 2017) |
|  | Daturametelin H |  | -5.62296104 | | Datura Metel | (Ma et al., 2006) |
|  | Geraniol |  | -5.62260532 | | Myristica Fragrans | (Ha et al., 2020) |
|  | 1,2,5,6-Tetrahydroxy xanthone |  | -5.62253237 | | Swertia Chirayita | (Mahendran et al., 2022) |
|  | Cyclo (Phe-Leu) |  | -5.61971378 | | Datura Metel | (Singh et al., 2012) |
|  | Catechin |  | -5.61599874 | | Myristica Fragrans | (Ousji & Sleno, 2022) |
|  | *cis*-*p-*Hydroxyphenyleth-anol-*p*- *β*-coumarate |  | -5.61354065 | | Datura Metel | (B. Y. Yang, 2005) |
|  | Withametelin J |  | -5.61216831 | | Datura Metel | (Pan, Wang, & Hu, 2007) |
|  | 1-Ribityl-2,3-diketo-1,2,3,4- tetrahydro-6,7-dimethyl-quinoxaline |  | | -5.61034966 | Datura Metel | (B. Yang, Xia, Wang, Dou, Fitoterapia, et al., 2010) |
|  | Pterodontriol B |  | -5.60075951 | | Datura Metel | (Aberham et al., 2010) |
|  | (6*R*,7*E*,9*R*)-9-Hydroxy-4,7- megastigmandien-3-one |  | -5.59540653 | | Datura Metel | (B. Yang et al., 2007) |
|  | Swerimilegenin I |  | -5.59201336 | | Swertia Chirayita | (Chen, 2017) |
|  | Baimantuoluoline L |  | -5.58584785 | | Datura Metel | (B. Y. Yang, 2005) |
|  | Dihydrocarinatidin |  | -5.5838275 | | Myristica Fragrans | (G.-Y. Cao et al., 2013) |
|  | Withametelin G |  | -5.5681448 | | Datura Metel | (Manickam et al., 1993) |
|  | Dmetelin A |  | -5.56680298 | | Datura Metel | (B. Yang, Guo, Li, Liu, et al., 2014) |
|  | *threo*-2,3-Bis-(4-hydroxy-3- methoxypheyl)-3-methoxy-propanol |  | -5.55611229 | | Datura Metel | (B.Y. Yang, C.L. Yang, Y. Liu, 2017) |
|  | Alpha-Muurolene |  | | -5.54681396 | Myristica Fragrans | (Ha et al., 2020) |
|  | Apiol |  | | -5.54520607 | Myristica Fragrans | (Ha et al., 2020) |
|  | Dmetelin D (terpenoid) |  | -5.53953695 | | Datura Metel | (J. Tan, Liu, Cheng, Sun, Pan, Fitoterapia, et al., 2020) |
|  | Anthriscinol |  | -5.53671265 | | Myristica Fragrans | (Morikawa et al., 2016a) |
|  | (6*R*,7*E*,9*R*)-9-hydroxymegastigma-4,7-dien-3-one-9-*O*-*β*-D-glucopyranoside |  | | -5.532022 | Swertia Chirayita | (Zhou et al., 2015) |
|  | Isofraxidin |  | -5.52950716 | | Datura Metel | (B.Y. Yang, H.B. Jiang, Y. Liu, Z.P. Xu, 2018) |
|  | Hyoscyamine |  | -5.5276432 | | Datura Metel | (Doncheva et al., 2006) |
|  | Dmetelin F |  | | -5.52333879 | Datura Metel | (J. Tan, Liu, Cheng, Sun, Pan, Fitoterapia, et al., 2020) |
|  | Baimantuoluoline K |  | | -5.51848888 | Datura Metel | (B. Yang, Xia, et al., 2014) |
|  | Baimantuoluoline E |  | -5.51687002 | | Datura Metel | (B. Yang et al., 2008) |
|  | ω-Hydroxypropioguaiacone |  | -5.51586294 | | Datura Metel | (B.Y. Yang, C.L. Yang, Y. Liu, 2017) |
|  | Daturafoliside S |  | -5.49213505 | | Datura Metel | (Gupta, Bagchi, & Ray, 1991) |
|  | Baimantuoluoline B |  | -5.48808384 | | Datura Metel | (B. Yang et al., 2007) |
|  | 12-Deoxywithastramonolide |  | -5.48686028 | | Datura Metel | (Gupta, Bagchi, & Ray, 1991) |
|  | Withatatulin D |  | -5.48523188 | | Datura Metel | (B. Yang et al., 2008) |
|  | 3-methyl-5-pentyl-furylarylic acid |  | -5.48244286 | | Myristica Fragrans | (Ha et al., 2020) |
|  | Citronellol |  | -5.47748327 | | Myristica Fragrans | (Ha et al., 2020) |
|  | Caffeic acid ethyl ester |  | -5.47747564 | | Datura Metel | (NĂNESCU et al., 2023) |
|  | Daturaolone |  | -5.45369005 | | Datura Metel | (Bawazeer et al., 2020) |
|  | 1,3-Dihydroxy xanthone |  | | -5.45183182 | Swertia Chirayita | (He et al., 2017) |
|  | *P*-hydroxybenzoyl *p*-coumaric acid anhydride |  | -5.45007086 | | Datura Metel | (J. Tan, Liu, Cheng, Sun, Pan, Fitoterapia, et al., 2020) |
|  | Daturmetelide A |  | -5.44583893 | | Datura Metel | (J. Tan, Liu, Cheng, Sun, Pan, Guan, et al., 2020) |
|  | Apigenin |  | | -5.44551802 | Myristica Fragrans | (Fabre et al., 2001) |
|  | Swertiachoside A |  | -5.44383478 | | Swertia Chirayita | (Zhou et al., 2015) |
|  | Withametelin E |  | -5.44244862 | | Datura Metel | (Gupta, Bagchi, Ray, et al., 1991) |
|  | Swertiachiralactone A |  | -5.44020939 | | Swertia Chirayita | (Zhou et al., 2015) |
|  | D-Germacrene |  | -5.43924093 | | Myristica Fragrans | (Trifan et al., 2023) |
|  | 1*β*,5*α*,7*β*-guaiane-4*β*,10*α*,11- triol |  | -5.42564392 | | Datura Metel | (Mai, Cuc, Anh, Nhiem, …, et al., 2017) |
|  | Daturglycoside G |  | -5.41220808 | | Datura Metel | (B.Y. Yang, C.L. Yang, Y. Liu, 2017) |
|  | Beta-caryophyllene |  | -5.4092288 | | Myristica Fragrans | (Trifan et al., 2023) |
|  | 1α,5α,7α-11-guaiene- 2α,3β,4α,10α,13-pentaol |  | -5.39712334 | | Datura Metel | (Mai, Cuc, Anh, Nhiem, …, et al., 2017) |
|  | (2*R*)-3-(3,4-Methylenedioxyphenyl)-1,2-propanediol |  | -5.38931847 | | Myristica Fragrans | (Duan et al., 2009b) |
|  | Methoxyeugenol |  | -5.38798475 | | Myristica Fragrans | (Trifan et al., 2023) |
|  | Daturametelindole D |  | -5.38409662 | | Datura Metel | (Liu et al., n.d.) |
|  | Linalool |  | -5.38106585 | | Myristica Fragrans | (Trifan et al., 2023) |
|  | methylisoeugenol |  | -5.37671757 | | Myristica Fragrans | (Trifan et al., 2023) |
|  | Isootobaphenol |  | -5.37509441 | | Myristica Fragrans | (Kwon et al., 2014) |
|  | Bridelionoside B |  | -5.37475204 | | Swertia Chirayita | (Zhou et al., 2015) |
|  | Daturaterpenoid D |  | -5.36240482 | | Datura Metel | (Aberham et al., 2010) |
|  | Ophiobolin A |  | -5.3584094 | | Datura Metel | (B.Y. Yang, C.L. Yang, Y. Liu, 2017) |
|  | 1*α*,5*α*,7*α*-11-Guaiene- 2*α*,3*β*,4*α*,10*α*,13-pentaol |  | -5.35553026 | | Datura Metel | (Mai, Cuc, Anh, Nhiem, …, et al., 2017) |
|  | Grasshopper ketone |  | -5.33768797 | | Datura Metel | (H. X. Kuang et al., 2008) |
|  | Withafastuosin E |  | -5.33653069 | | Datura Metel | (Manickam et al., 1998) |
|  | Daturmetelide H |  | -5.33423662 | | Datura Metel | (J. Tan, Liu, Cheng, Sun, Pan, Guan, et al., 2020) |
|  | Withametelin D |  | -5.33385754 | | Datura Metel | (B. Yang et al., 2007) |
|  | Myristicin |  | -5.33331156 | | Myristica Fragrans | (Trifan et al., 2023) |
|  | N-trans-cinnamoyl  tyramine |  | -5.32999325 | | Datura Metel | (B.Y. Yang, C.L. Yang, Y. Liu, 2017) |
|  | methyleugenol |  | -5.32677174 | | Myristica Fragrans | (Trifan et al., 2023) |
|  | Lycium substance B |  | -5.32341194 | | Datura Metel | (Gupta, Bagchi, Ray, et al., 1991) |
|  | Butyl-*β*-D-glucopyranoside |  | -5.31165218 | | Datura Metel | Tan et al. (2022) |
|  | Yangjinhualine A |  | -5.30990362 | | Datura Metel | (H. X. Kuang et al., 2008) |
|  | 3′,4′,7-trihydroxyflavone |  | -5.30500507 | | Myristica Fragrans | (Dzotam et al., 2018) |
|  | 9-Hydroxycanthin-6-one |  | -5.30426788 | | Datura Metel | (B.Y. Yang, Y.M. Luo, Y. Liu, X. Yin, Y.Y. Zhou, 2018) |
|  | Merobatzelladine A |  | -5.30229664 | | Datura Metel | (Mai, Cuc, Anh, Nhiem, Steroids, et al., 2017) |
|  | 2,6-Dimethyl 2,6-Octadiene |  | -5.30164671 | | Myristica Fragrans | (Ha et al., 2020) |
|  | Stigmasterol |  | | -5.29857302 | Swertia Chirayita | (Pant et al., 2002) |
|  | Withametelin C 1 |  | -5.29246378 | | Datura Metel | (Manickam et al., 1996) |
|  | 2,3-Dimethoxy-5-(1-propenyl)-phenol |  | -5.2761755 | | Myristica Fragrans | (Kimura et al., 2010) |
|  | Scopoletin |  | -5.26255846 | | Swertia Chirayita | (You et al., 2017) |
|  | Dmetelin H |  | | -5.25645685 | Datura Metel | (J. Tan, Liu, Cheng, Sun, Pan, Fitoterapia, et al., 2020) |
|  | Chiratenol |  | -5.25449038 | | Swertia Chirayita | (Chakravarty et al., 1990) |
|  | Sinapaldehyde |  | -5.25081301 | | Swertia Chirayita | (You et al., 2017) |
|  | Daturametelindole A |  | -5.22786093 | | Datura Metel | (Liu et al., n.d.) |
|  | Gentiopicrin |  | -5.22270775 | | Swertia Chirayta | (Patel et al., 2021) |
|  | Daturafoliside M |  | -5.21978188 | | Datura Metel | (B. Yang, Guo, Li, Wu, et al., 2014) |
| 1. s | Corchoionoside C |  | -5.21420145 | | Datura Metel | (H. X. Kuang et al., 2008) |
|  | Elcosane |  | -5.21363544 | | Myristica Fragrans | (Ha et al., 2020) |
|  | 5-Hydroxyeugenol |  | -5.21351671 | | Myristica Fragrans | (Kapoor et al., 2013) |
|  | Trans-beta-ocimene |  | -5.20889568 | | Myristica Fragrans | (Ha et al., 2020) |
|  | Guaiol |  | -5.20519495 | | Myristica Fragrans | (Trifan et al., 2023) |
|  | (8*R*)-2′ -Acetoxyl-7-phenyl-9- propanol |  | -5.20276499 | | Datura Metel | (B. Y. Yang, 2005) |
|  | Baimantuoluoside C |  | -5.18657398 | | Datura Metel | (H. Kuang et al., 2009) |
|  | eugenol |  | -5.18248892 | | Myristica Fragrans | (Trifan et al., 2023) |
|  | phenylpropanoids myristicin |  | -5.18071318 | | Myristica Fragrans | (Trifan et al., 2023) |
|  | Alpha-Cubebene |  | -5.16374207 | | Myristica Fragrans | (Trifan et al., 2023) |
|  | Diphenylbutane 1 |  | -5.16186714 | | Myristica Fragrans | (Trifan et al., 2023) |
|  | Beta-Terpinyl Acetate |  | -5.15255594 | | Myristica Fragrans | (Trifan et al., 2023) |
|  | Dmetelin B |  | -5.14507675 | | Datura Metel | (B. Yang, Guo, Li, Liu, et al., 2014) |
|  | Cis-p-menth-2-en-1-ol |  | -5.14182043 | | Myristica Fragrans | (Trifan et al., 2023) |
|  | (+)-Dehydrovomifoliol |  | -5.12699509 | | Datura Metel | (B. Yang et al., 2007) |
|  | 1β,5α,7β-guaiane- 4β,10α,11-triol |  | -5.12380171 | | Datura Metel | (Mai, Cuc, Anh, Nhiem, …, et al., 2017) |
|  | Tropic acid |  | -5.11889601 | | Datura Metel | (Z.Y. Li, H.X. Kuang, Y.G. Xia, 2010) |
|  | Disciferitriol |  | -5.11357021 | | Datura Metel | (Mai, Cuc, Anh, Nhiem, …, et al., 2017) |
|  | sabinene |  | -5.11284065 | | Myristica Fragrans | (Trifan et al., 2023) |
|  | Syringic acid |  | -5.09556675 | | Swertia Chirayita | (Chen, 2017) |
|  | Myrecene |  | -5.08210611 | | Myristica Fragrans | (Trifan et al., 2023) |
|  | Naringenin |  | -5.06599426 | | Myristica Fragrans | (Fabre et al., 2001) |
|  | Methyl (3*β*)-3-hydroxyurs-12-en-28-oate |  | -5.06558275 | | Swertia Chirayita | (Shi et al., 2013) |
|  | Vanilic acid |  | -5.06404495 | | Datura Metel | (Gutierrez-Lugo et al., 2005) |
|  | Datumetine |  | -5.0615983 | | Datura Metel | (Gutierrez-Lugo et al., 2005) |
|  | 3-Hydroxy-6- propionyloxytropane |  | -5.06064653 | | Datura Metel | (Doncheva et al., 2006) |
|  | Trans-piperitol |  | -5.05306578 | | Myristica Fragrans | (Jaiswal et al., 2009) |
|  | (2*R*)-3-(5-Methoxy-3,4-methylenedioxyphenyl)-1,2-propanedio |  | -5.0398283 | | Myristica Fragrans | (Duan et al., 2009b) |
|  | a-pinene |  | -5.03281116 | | Myristica Fragrans | (Trifan et al., 2023) |
|  | Tetramethylhexestrol |  | -5.01744938 | | Myristica Fragrans | (Ha et al., 2020) |
|  | Hyoscyamilactol |  | -5.01495981 | | Datura Metel | (B.Y. Yang, H.B. Jiang, Y. Liu, Z.P. Xu, 2018) |
|  | 2,5-dihydroxy terepthalic acid |  | | -5.01061678 | Swertia Chirayita | (Hatjimanoli et al., 1988) |
|  | p-Cymen-8-ol |  | -5.00626802 | | Myristica Fragrans | (Trifan et al., 2023) |
|  | 2-epi-corosolic acid |  | -5.0032239 | | Swertia Chirayita | (Chen, 2017) |
|  | Thymidine |  | -4.99769449 | | Datura Metel | (Mai, Cuc, Anh, Nhiem, Steroids, et al., 2017) |
|  | Daturaterpenoid A |  | -4.98885059 | | Datura Metel | (Liu et al., 2019) |
|  | Daturanolide C |  | -4.98553181 | | Datura Metel | (X.-Y. Wang et al., 2019) |
|  | Trans-p-Menth-2-en-1-ol |  | -4.96163177 | | Myristica Fragrans | (Trifan et al., 2023) |
|  | Syringaldehyde |  | -4.95767355 | | Swertia Chirayita | (You et al., 2017) |
|  | Alpha-thujene |  | -4.95542812 | | Myristica Fragrans | (Trifan et al., 2023) |
|  | 12-Hydroxyoleanolic lactone |  | -4.9530654 | | Swertia Chirayita | (Woo et al., 2019) |
|  | *β*-Sitosterol |  | -4.94857311 | | Swertia Chirayita | (L. Cai, S. Wang, T. Li, 2006) |
|  | Dmetelin G |  | -4.93532133 | | Datura Metel | (J. Tan, Liu, Cheng, Sun, Pan, Fitoterapia, et al., 2020) |
|  | Sweroside |  | -4.92444944 | | Swertia Chirayita | (L. Cai, S. Wang, T. Li, 2006) |
|  | Stigmast-4-en-3-one |  | -4.90764332 | | Swertia Chirayita | (Chakravarty"· et al., 2001) |
|  | Daturametelindole B |  | -4.90608072 | | Datura Metel | (Liu et al., n.d.) |
|  | beta-pinene |  | -4.89130259 | | Myristica Fragrans | (Trifan et al., 2023) |
|  | (1*R*,2*R*)-1-(4-Hydroxy-3-methoxyphenyl)-1,2-propanediol |  | -4.88746071 | | Myristica Fragrans | (Duan et al., 2009b) |
|  | Gentianine |  | -4.88216782 | | Swertia Chirayita | (Sharma, 1982) |
|  | Isololiolide |  | -4.88103914 | | Datura Metel | (X.-Y. Wang et al., 2019) |
|  | (1R,2R)-1-(4-hydroxy-  3-methoxyphenyl)-1,2-propanediol |  | -4.87465191 | | Myristica Fragrans | (Pham et al., 2005) |
|  | Erythrodiol |  | -4.87201452 | | Swertia Chirayita | (A.K. Chakravarty, S. Mukhopadhyay, K. Masuda, 1992) |
|  | Daturameteline K 19 |  | -4.85969353 | | Datura Metel | (Tang, 2006) |
|  | Vanillic acid |  | -4.85796547 | | Swertia Chirayita | (L. Cai, S. Wang, T. Li, 2006) |
|  | Naphthisoxazol A |  | -4.8577528 | | Datura Metel | (Zanousi et al., 2016) |
|  | 4-allyl-2,6-dimethoxyphenol |  | -4.84121227 | | Myristica Fragrans | (Forrest et al., 1974) |
|  | Alpha-copaene |  | -4.82386541 | | Myristica Fragrans | (Trifan et al., 2023) |
|  | p-hydroxy-ben-zenepropanoic acid |  | -4.82240868 | | Datura Metel | (Romero-Cortes et al., 2019) |
|  | *p*-Hydroxyphenylethanol |  | | -4.79311752 | Datura Metel | (B. Y. Yang, 2005) |
|  | Fraxetin |  | -4.78837538 | | Datura Metel | (B.Y. Yang, H.B. Jiang, Y. Liu, Z.P. Xu, 2018) |
|  | *Ψ*-Taraxasterol or heterolupeol |  | -4.78675079 | | Swertia Chirayita | (A.K. Chakravarty, S. Mukhopadhyay, K. Masuda, 1992) |
|  | Djalonenol |  | -4.78009319 | | Swertia Chirayita | (Woo et al., 2019) |
|  | Caffeic acid |  | -4.77604294 | | Myristica Fragrans | (Shan et al., 2005) |
|  | Carvacrol |  | -4.76305962 | | Myristica Fragrans | (Jaiswal et al., 2009) |
|  | Lupeol |  | -4.75923538 | | Swertia Chirayita | (A.K. Chakravarty, S. Mukhopadhyay, K. Masuda, 1992) |
|  | Beta-Phellandrene |  | -4.74305391 | | Myristica Fragrans | (Jaiswal et al., 2009) |
|  | 2-hydroxy terepthalic acid |  | -4.73999214 | | Swertia Chirayita | (Hatjimanoli et al., 1988) |
|  | Safrole |  | -4.73983908 | | Myristica Fragrans | (Trifan et al., 2023) |
|  | 1,8-Cineole |  | -4.73888731 | | Myristica Fragrans | (Jaiswal et al., 2009) |
|  | Beta-Fenchyl Alcohol |  | | -4.73441076 | Myristica Fragrans | (Ha et al., 2020) |
|  | Erythrocentaurin |  | -4.73289442 | | Swertia Chirayita | (He et al., 2017) |
|  | Terpinolene |  | -4.71702242 | | Myristica Fragrans | (Trifan et al., 2023) |
|  | Enicoflavine |  | -4.70904255 | | Swertia Chirayita | (Sharma, 1982) |
| 1. ss | Daturametelin C |  | -4.69601059 | | Datura Metel | (SHINGU et al., 1989) |
|  | Kairatenol |  | -4.6706562 | | Swertia Chirayita | (Chakravarty et al., 1992) |
|  | Erythrocentauric acid |  | -4.6663394 | | Swertia Chirayita | (He et al., 2017) |
|  | 4-terpineol |  | | -4.66067743 | Myristica Fragrans | (Trifan et al., 2023) |
|  | 3-carene |  | -4.65643263 | | Myristica Fragrans | (Trifan et al., 2023) |
|  | Methyl benzoate |  | -4.65105677 | | Datura Metel | (Z.Y. Li, H.X. Kuang, Y.G. Xia, 2010) |
|  | camphene |  | | -4.65019655 | Myristica Fragrans | (Trifan et al., 2023) |
|  | Cis-Sabinene Hydrate |  | -4.63727236 | | Myristica Fragrans | (Jaiswal et al., 2009) |
|  | gamma-terpinene |  | -4.63417959 | | Myristica Fragrans | (Trifan et al., 2023) |
|  | 2,3-dihydroxy-5-(hydroxymethyl)  benzaldehyde |  | -4.6340766 | | Myristica Fragrans | (Ha et al., 2020) |
|  | vanillin |  | -4.624547 | | Myristica Fragrans | (Ha et al., 2020) |
|  | *β*-amyrin |  | -4.61440325 | | Swertia Chirayita | (Sharma, 1983) |
|  | 4-Thujanol |  | -4.6132884 | | Myristica Fragrans | (Jaiswal et al., 2009) |
|  | limonene |  | **-4.60795164** | | Myristica Fragrans | (Trifan et al., 2023) |
|  | p-coumaric acid |  | | -4.5993557 | Datura Metel | (Romero-Cortes et al., 2019) |
|  | Alpha-Terpinene |  | -4.5902853 | | Myristica Fragrans | (Trifan et al., 2023) |
|  | Methyl *p*-hydroxybenzoate |  | -4.58693027 | | Datura Metel | (B. Y. Yang, 2005) |
|  | Swertanone |  | -4.5512042 | | Swertia Chirayita | (Chakravarty et al., 1989) |
|  | p-hydroxyacetophenone |  | -4.54687691 | | Datura Metel | (Romero-Cortes et al., 2019) |
|  | 4-Hydroxyacetophenone |  | -4.53789425 | | Datura Metel | (Z.Y. Li, H.X. Kuang, Y.G. Xia, 2010) |
|  | Alpha-phellandrene |  | -4.50590086 | | Myristica Fragrans | (Trifan et al., 2023) |
|  | Methyl-3-hydroxy-2-phenyl propionic acid |  | -4.5007081 | | Datura Metel | (Z.Y. Li, H.X. Kuang, Y.G. Xia, 2010) |
|  | *trans*-Isoeugenol |  | -4.47794247 | | Myristica Fragrans | (Kapoor et al., 2013) |
|  | Gentiocrucine |  | | -4.42875862 | Swertia Chirayita | (Sharma, 1982) |
|  | Esculetin |  | | -4.42432928 | Datura Metel | (Alam et al., 2020) |
|  | 2,5-dihydroxyterephthalic |  | -4.42035532 | | Myristica Fragrans | (Isogai et al., 2014) |
|  | Cholest-4-en 3-one |  | -4.39250612 | | Swertia Chirayita | (Zhou et al., 2015) |
|  | *m*-hydroxybenzoic acid |  | | -4.37509298 | Swertia Chirayita | (L. Cai, S. Wang, T. Li, 2006) |
|  | 3,4-Dihydroxytoluene |  | -4.3718853 | | Datura Metel | (Z.Y. Li, H.X. Kuang, Y.G. Xia, 2010) |
|  | Protocatechuic acid |  | -4.36950731 | | Datura Metel | (NĂNESCU et al., 2023) |
|  | Norharman |  | -4.36945486 | | Datura Metel | (Chakravarty"· et al., 2001) |
|  | alpha-terpineol |  | -4.35955 | | Myristica Fragrans | (Trifan et al., 2023) |
|  | (3*β*,13*α*,14*β*,20*α*)-3-hydroxy-13-methyl-26-norolean-8-en-29-oic acid |  | -4.35318708 | | Swertia Chirayita | (Shi et al., 2013) |
|  | p-cymene |  | -4.350811 | | Myristica Fragrans | (Trifan et al., 2023) |
|  | Taraxerol |  | -4.31812 | | Swertia Chirayita | (Chakravarty et al., 1991) |
|  | Moretenol |  | -4.30757093 | | Swertia Chirayita | (Chakravarty et al., 1991) |
|  | Olean-12-ene-18*α*H-3-one-19*β*-ol |  | -4.25214911 | | Swertia Chirayita | (Bajaj et al., 2017) |
|  | Protocatechuic aldehyde |  | | -4.20647049 | Datura Metel | (NĂNESCU et al., 2023) |
|  | Olean-12-en-18*α*H-3-one |  | -4.19588566 | | Swertia Chirayita | (Bajaj et al., 2017) |
|  | Saychellene |  | -4.17844248 | | Myristica Fragrans | (Trifan et al., 2023) |
|  | Methyl (3*β*,13*α*,14*β*,20*α*)-3-hydroxy-13-methyl-26-norolean-8-en- 29-oate |  | -4.08488941 | | Swertia Chirayita | (Shi et al., 2013) |
|  | Pyrocatechol |  | -4.08429813 | | Swertia Chirayita | (Chen, 2017) |
|  | Olean-12-ene-18*α*H-3-one-9*α*-ol |  | -4.00022554 | | Swertia Chirayita | (Bajaj et al., 2017) |
|  | Cis-piperitol |  | -3.60624242 | | Myristica Fragrans | (Jaiswal et al., 2009) |
|  | Friedelin |  | -3.18901706 | | Swertia Chirayita | (Sabita et al, 2019) |
|  | Daturametelindole B |  | -4. 44701242 | | Datura Metel | (Liu et al., n.d.) |
|  | Daturametelindole D |  | -4. 72291543 | | Datura Metel | (Liu et al., n.d.) |
|  | Daturametelindole C |  | | -3. 81242127 | Datura Metel | (Liu et al., n.d.) |
|  | Gammacer-16-en-3-*β*-ol |  | Inactive | | Swertia Chirayita | (Chakravarty et al., 1991) |
|  | Swertenol |  | Inactive | | Swertia Chirayita | (Chakravarty et al., 1991) |
|  | Episwertenol |  | Inactive | | Swertia Chirayita | (Chakravarty et al., 1991) |
|  | Swerta-7,9(11)-dien-3-*β*-ol |  | Inactive | | Swertia Chirayita | (A.K. Chakravarty, S. Mukhopadhyay, K. Masuda, 1992) |
|  | Pichierenol |  | Inactive | | Swertia Chirayita | (A.K. Chakravarty, S. Mukhopadhyay, K. Masuda, 1992) |
|  | Chirat-16-en-3-*β*-24-diol |  | | Inactive | Swertia Chirayita | (Chakravarty"· et al., 2001) |
|  | Oleanolic acid |  | Inactive | | Swertia Chirayita | (L. Cai, S. Wang, T. Li, 2006) |
|  | Betulinic acid |  | Inactive | | Swertia Chirayita | (Kaur et al., 2019) |
|  | Ursolic acid |  | Inactive | | Swertia Chirayita | (Kaur et al., 2019) |
|  | Baimantuoluoline F 2 |  | Inactive | | Datura Metel | (B. Yang et al., 2008) |
|  | Baimantuoluoline C |  | Inactive | | Datura Metel | (B. Yang et al., 2007) |
|  | Daturameteline E |  | Inactive | | Datura Metel | (B. Yang et al., 2007) |
|  | Withafastuosin F |  | Inactive | | Datura Metel | (B. Yang et al., 2008) |
|  | Baimantuoluoline P |  | Inactive | | Datura Metel | (Liu et al., 2020) |
|  | Baimantuoluoline A |  | Inactive | | Datura Metel | (Gupta, Bagchi, Ray, et al., 1991) |
|  | Daturameteloside A |  | Inactive | | Datura Metel | (H. Kuang et al., 2009) |
|  | Baimantuoluoline O |  | Inactive | | Datura Metel | (Liu et al., 2020) |
|  | Daturafoliside Q |  | Inactive | | Datura Metel | (B. Y. Yang et al., 2014) |
|  | Daturameteline H |  | Inactive | | Datura Metel | (B. Y. Yang, 2005) |
|  | Daturmetelide S |  | Inactive | | Datura Metel | (J. Tan, Liu, Cheng, Sun, Pan, Guan, et al., 2020) |
|  | Baimantuoluoline T |  | | Inactive | Datura Metel | (Liu et al., 2020) |
|  | Withametelin |  | Inactive | | Datura Metel | (Gupta, Bagchi, & Ray, 1991) |
|  | 12*β*-Hydroxy-1,10-seco-withametelin B |  | | Inactive | Datura Metel | (Pan, Wang, & Hu, 2007) |
|  | Dihydrophaseic acid |  | | Inactive | Datura Metel | (Pan, Wang, & Hu, 2007) |
|  | 4′ -Dihydrophaseic acid *β*-glucopyranose ester |  | | Inactive | Datura Metel | (R. Guo, Y. Liu, J. Pan, W. Guan, B.Y. Yang, n.d.) |
|  | Oleracone B |  | Inactive | | Datura Metel | (X.-Y. Wang et al., 2019) |
|  | Atractylenother |  | | Inactive | Datura Metel | (J. Tan, Liu, Cheng, Sun, Pan, Fitoterapia, et al., 2020) |
|  | Kaurane acid glycoside C |  | Inactive | | Datura Metel | (B. you Yang et al., 2018) |
|  | Paniculoside |  | | Inactive | Datura Metel | (B. you Yang et al., 2018) |
|  | Meteloside F |  | Inactive | | Datura Metel | (B. Y. Yang et al., 2020) |
|  | Meteloside G |  | | Inactive | Datura Metel | (B. Y. Yang et al., 2020) |
|  | Paeoniflorin |  | Inactive | | Datura Metel | B. Yang, Yang, et al., 2018) |
|  | Ginsenoside Re |  | | Inactive | Datura Metel | B. Yang, Yang, et al., 2018) |
|  | (7S, 8R, 80S, 70S) 7,70-bis(3-hydroxy-5-methoxyphenyl)-8,80-dimethylbutane- 7,70-diol |  | Inactive | | Myristica Fragrans |  |
|  | Bornylacetate |  | Inactive | | Myristica Fragrans | (Trifan et al., 2023) |
|  | sitosterol 3-*O*-[*β*-d-glucopyranos-6′-yl  tetradecanoate |  | Inactive | | Myristica Fragrans | (Hou et al., 2012) |

**References**

A.K. Chakravarty, S. Mukhopadhyay, K. Masuda, H. A. (1992). *Chakravarty: Swertane triterpenoids from Swertia chirata - Google Scholar*.

Aberham, A., Cicek, S. S., Schneider, P., & Stuppner, H. (2010). Analysis of sesquiterpene lactones, lignans, and flavonoids in wormwood (Artemisia absinthium L.) using high-performance liquid chromatography (HPLC)-mass spectrometry, reversed phase HPLC, and HPLC-Solid phase extraction-nuclear magnetic resonance. *Journal of Agricultural and Food Chemistry*, *58*(20), 10817–10823. https://doi.org/10.1021/jf1022059

Adjene, J. O., & Igbigbi, P. S. (2010). Effect of Chronic Consumption of Nutmeg on the Stomach of Adult Wistar Rats. *Fooyin Journal of Health Sciences*, *2*(2), 62–65. https://doi.org/10.1016/S1877-8607(10)60017-3

Al-Qahtani, W., Dinakarkumar, Y., … S. A.-… J. of B., & 2022, U. (2022). Phyto-chemical and biological activity of Myristica fragrans, an ayurvedic medicinal plant in Southern India and its ingredient analysis. *Elsevier*.

Alam, W., Khan, H., Khan, S. A., Nazir, S., & Akkol, E. K. (2020). Datura metel: A Review on Chemical Constituents, Traditional Uses and Pharmacological Activities. *Current Pharmaceutical Design*, *27*(22), 2545–2557. https://doi.org/10.2174/1381612826666200519113752

Andrade et al. (2011). Scholar (6). In *Instituto Universitario de Educación Física y Deporte* (Vol. 9, Issue 2, pp. 43–56).

B.Y. Yang, C.L. Yang, Y. Liu, H. X. K. (2017). Scholar 下午9. In *Mass Communication and Society* (Vol. 10, Issue 10, pp. 349–383).

B.Y. Yang, H.B. Jiang, Y. Liu, Z.P. Xu, H. X. K. (2018). Chemical constituents from seeds of Datura metel (I). *Pesquisa.Bvsalud.OrgBY YANGChinese Traditional and Herbal Drugs, 2013•pesquisa.Bvsalud.Org*.

B.Y. Yang, Y.M. Luo, Y. Liu, X. Yin, Y.Y. Zhou, H. X. K. (2018). *scholar (5)*.

Bajaj, S., Singh, V., Life, M. A.-A. S. of, & 2017, U. (2017). New triterpenic compounds from Swertia chirata. *Journals.Lww.Com*.

Bajpai, M. B., Asthana, R. K., Sharma, N. K., Chatterjee, S. K., & Mukherjee, S. K. (1991). Hypoglycemic effect of swerchirin from the hexane fraction of Swertia chirayita. *Planta Medica*, *57*(2), 102–104. https://doi.org/10.1055/S-2006-960041

Barclay, A. (1995). New considerations in an old genus: Datura. *JSTOR*.

Bawazeer, S., Rauf, A., & Bawazeer, S. (2020). Gastrointestinal Motility, Muscle Relaxation, Antipyretic and Acute Toxicity Screening of Amyrin Type Triterpenoid (Daturaolone) Isolated From Datura metel Linnaeus (Angel’s Trumpet) Fruits. *Frontiers in Pharmacology*, *11*. https://doi.org/10.3389/FPHAR.2020.544794/FULL

Bellila, A., Tremblay, C., Pichette, A., Phytochemistry, B. M.-, & 2011, U. (2011). Cytotoxic activity of withanolides isolated from Tunisian Datura metel L. *Elsevier*.

Bhandari, P., Kumar, N., Gupta, A. P., Singh, B., & Kaul, V. K. (2006). Micro-LC determination of swertiamarin in Swertia species and bacoside-A in Bacopa monnieri. *Chromatographia*, *64*(9–10), 599–602. https://doi.org/10.1365/S10337-006-0065-X

Cao, G.-Y., Xu, W., Yang, X.-W., Gonzalez, F. J., & Li, F. (2015). New neolignans from the seeds of Myristica fragrans that inhibit nitric oxide production. *Food Chemistry*, *173*, 231–237. https://doi.org/10.1016/j.foodchem.2014.09.170

Cao, G.-Y., Yang, X.-W., Xu, W., & Li, F. (2013). New inhibitors of nitric oxide production from the seeds of Myristica fragrans. *Food and Chemical Toxicology*, *62*, 167–171. https://doi.org/10.1016/j.fct.2013.08.046

Cao, G., Xu, W., Yang, X., Gonzalez, F., Chemistry, F. L.-F., & 2015, U. (2015). New neolignans from the seeds of Myristica fragrans that inhibit nitric oxide production. *ElsevierGY Cao, W Xu, XW Yang, FJ Gonzalez, F LiFood Chemistry, 2015•Elsevier*.

Chakravarty"·, A. K., Sarkar, T., Das, B., Masudah, K., & Shiojimah, K. (2001). A new chiratane triterpenoid from Swertia chirata. *Indian Journal of Chemi Stry*, *408*, 228–231.

Chakravarty, A., Das, B., … S. P.-J. of the, & 1989, U. (1989). X-Ray crystal structure of swertanone, a triterpene of new skeletal type from Swertia chirata Buch.-Ham. *Pubs.Rsc.OrgAK Chakravarty, B Das, SC Pakrashi, DR McPhail, AT McPhailJournal of the Chemical Society, Chemical Communications, 1989•pubs.Rsc.Org*.

Chakravarty, A., Das, B., Masuda, K., Letters, H. A.-T., & 1990, U. (1990). Chiratenol, a novel rearranged hopane triterpenoid fromswertia chirata. *Elsevier*.

Chakravarty, A., Mukhopadhyay, S., … S. M.-I. J. C., & 1994, U. (1994). Syringareinol, a hepatoprotective agent and other constituents from Swertia chirata. *Hero.Epa.Gov*.

Chakravarty, A., Mukhopadhyay, S., Letters, K. M.-T., & 1992, U. (1992). Kairatenol, yet another novel migrated gammacerane triterpenoid from Swertia chirata. *Elsevier*.

Chakravarty, A., Mukhopadhyay, S., Phytochemistry, B. D.-, & 1991, U. (1991). Swertane triterpenoids from Swertia chirata. *Elsevier*.

Chen, X. Q. (2017). Chemical constituents from Tibetan medicine Swertia chirayita. *Pesquisa.Bvsalud.OrgXQ CHENChinese Traditional and Herbal Drugs, 2017•pesquisa.Bvsalud.Org*.

Chiu, S., Wang, T., Belski, M., & Abourashed, E. A. (2016a). HPLC-Guided Isolation, Purification and Characterization of Phenylpropanoid and Phenolic Constituents of Nutmeg Kernel ( *Myristica fragrans* ). *Natural Product Communications*, *11*(4), 1934578X1601100. https://doi.org/10.1177/1934578X1601100416

Chiu, S., Wang, T., Belski, M., & Abourashed, E. A. (2016b). HPLC-guided isolation, purification and characterization of phenylpropanoid and phenolic constituents of nutmeg kernel (Myristica fragrans). *Natural Product Communications*, *11*(4), 483–488. https://doi.org/10.1177/1934578X1601100416

Chumkaew, P., & Srisawat, T. (2019). New neolignans from the seeds of Myristica fragrans and their cytotoxic activities. *Journal of Natural Medicines*, *73*(1), 273–277. https://doi.org/10.1007/s11418-018-1246-2

Doncheva, T., Berkov, S., Ecology, S. P.-B. S. and, & 2006, U. (2006). Comparative study of the alkaloids in tribe Datureae and their chemosystematic significance. *Elsevier*.

Duan, L., Tao, H., Hao, X., Gu, Q., Medica, W. Z.-P., & 2009, undefined. (2009a). Cytotoxic and antioxidative phenolic compounds from the traditional Chinese medicinal plant, Myristica fragrans. *Thieme-Connect.Com*, *75*(11), 1241–1245. https://doi.org/10.1055/s-0029-1185506

Duan, L., Tao, H., Hao, X., Gu, Q., Medica, W. Z.-P., & 2009, undefined. (2009b). Cytotoxic and antioxidative phenolic compounds from the traditional Chinese medicinal plant, Myristica fragrans. *Thieme-Connect.ComL Duan, HW Tao, X Hao, QQ Gu, WM ZhuPlanta Medica, 2009•thieme-Connect.Com*, *75*(11), 1241–1245. https://doi.org/10.1055/s-0029-1185506

Dzotam, J. K., Konga Simo, I., Bitchagno, G., Celik, I., Sandjo, L. P., Tane, P., & Kuete, V. (2018). In vitro antibacterial and antibiotic modifying activity of crude extract, fractions and 3′,4′,7-trihydroxyflavone from Myristica fragrans Houtt against MDR Gram. *Springer*, *18*(1). https://doi.org/10.1186/s12906-018-2084-1

Fabre, N., Rustan, I., De Hoffmann, E., & Quetin-Leclercq, J. (2001). Determination of flavone, flavonol, and flavanone aglycones by negative ion liquid chromatography electrospray ion trap mass spectrometry. *Journal of the American Society for Mass Spectrometry*, *12*(6), 707–715. https://doi.org/10.1016/S1044-0305(01)00226-4

Flannery, B.~P.Teukolsky, S. ~A., Vetterling, W. ~T., Leckenby, J., Li, H., Bruns, A., Danowski, J., Choi, J., Zill, D GPress, W. ~H., Wright, W. S., Optiz, G., Team, R. C., Payerle, G., Chapra, S. C., Canale, R. P., Analysis, N., Floudas, C. a, Pardalos, P. M., Negus, K., Pickering, M., … Horkheimer, M. (2004). Scholar (8). In *Convergence in the information industries. Telecommunications, broadcasting and data processing 1981-1996* (Vol. 26, Issue 1, pp. 125–150).

Forrest, J., Heacock, R., Society, T. F.-J. of the C., & 1974, U. (1974). Diarylpropanoids from nutmeg and mace (Myristica fragrans Houtt.). *Pubs.Rsc.Org*.

Francis, K., Suresh, E., Research, M. N.-N. P., & 2014, undefined. (2014). Chemical constituents from Myristica fragrans fruit. *Taylor & FrancisKS Francis, E Suresh, MS NairNatural Product Research, 2014•Taylor & Francis*, *28*(20), 1664–1668. https://doi.org/10.1080/14786419.2014.934236

Francis, S., James, B., … S. V.-N. P., & 2019, undefined. (2019). Phytochemical investigation on Myristica fragrans stem bark. *Taylor & FrancisSK Francis, B James, S Varughese, MS NairNatural Product Research, 2019•Taylor & Francis*, *33*(8), 1204–1208. https://doi.org/10.1080/14786419.2018.1457670

Francis, S. K., James, B., Varughese, S., & Nair, M. S. (2019). Phytochemical investigation on *Myristica fragrans* stem bark. *Natural Product Research*, *33*(8), 1204–1208. https://doi.org/10.1080/14786419.2018.1457670

Ghosal, S., Sharma, P. V., Chaudhuri, R. K., & Bhattacharya, S. K. (1973). Chemical constituents of the gentianaceae V: Tetraoxygenated xanthones of swertia chirata buch.‐ham. *Journal of Pharmaceutical Sciences*, *62*(6), 926–930. https://doi.org/10.1002/jps.2600620614

Gupta, M., Bagchi, A., & Ray, A. B. (1991). Additional withanolides of datura metel. *Journal of Natural Products*, *54*(2), 599–602. https://doi.org/10.1021/NP50074A042

Gupta, M., Bagchi, A., Ray, A. B., Hikino, H., Sahai, M., Ray, A., & Chem Soc Chem Commun, J. (1991). Additional withanolides of datura metel. *Journal of Natural Products*, *54*(2), 599–602. https://doi.org/10.1021/NP50074A042

Gutierrez-Lugo, M.-T., Woldemichael, G. M., Singh, M. P., Suarez, P. A., Maiese, W. M., Montenegro, G., Timmermann, B. N., Gutierrez-Lugoy, M.-T., Woldemichaely, G. M., Singhz, M. P., Suarezz, P. A., Maiesez, W. M., Montenegrox, G., & Timmermanny, B. N. (2005). Isolation of three new naturally occurring compounds from the culture of Micromonospora sp. P1068. *Taylor & FrancisMT Gutierrez-Lugo, GM Woldemichael, MP Singh, PA Suarez, WM Maiese, G MontenegroNatural Product Research, 2005•Taylor & Francis*, *19*(7), 645–652. https://doi.org/10.1080/14786410412331272040

H. Gan, J. He, X.Y. Wang, Y.W. Wang, H.M. Gao, H. Jiang, W.K. Zhang, J. K. X. (2021). *Gan: Chemical constituents from the dry flowers of... - Google Scholar*.

Ha, M. T., Khanh Vu, N., Tran, T. H., Jeong, ·, Kim, A., Woo, M. H., Byung, ·, & Min, S. (2020). Phytochemical and pharmacological properties of Myristica fragrans Houtt.: an updated review. *SpringerMT Ha, NK Vu, TH Tran, JA Kim, MH Woo, BS MinArchives of Pharmacal Research, 2020•Springer*, *43*(11), 1067–1092. https://doi.org/10.1007/s12272-020-01285-4

Hada, S., Hattori, M., Tezuka, Y., Kikuchi, T., & Namba, T. (1988). New neolignans and lignans from the aril of Myristica fragrans. *Phytochemistry*, *27*(2), 563–568. https://doi.org/10.1016/0031-9422(88)83142-X

Hada, S., Hattori, M., Tezuka, Y., Kikuchi, T., Phytochemistry, T. N.-, & 1988, U. (1988). New neolignans and lignans from the aril of Myristica fragrans. *Elsevier*.

Hatjimanoli, M., Favre-Bonvin, J., Kaouadji, M., & Mariotte, A. M. (1988). Monohydroxy— and 2,5-dihydroxy terephthalic acids, two unusual phenolics isolated from centaurium erythraea and identified in other gentianaceae members. *Journal of Natural Products*, *51*(5), 977–980. https://doi.org/10.1021/NP50059A030

Hattori, M., Hada, S., Kawata, Y., Tezuka, Y., Kikuchi, T., & Namba, T. (1987). New 2, 5-bis-aryl-3, 4-dimethyltetrahydrofuran lignans from the aril of Myristica fragrans. *Jstage.Jst.Go.JpM Hattori, S Hada, Y Kawata, Y Tezuka, T Kikuchi, T NambaChemical and Pharmaceutical Bulletin, 1987•jstage.Jst.Go.Jp*, *35*(8), 3315–3322.

HATTORI, M., HADA, S., SHU, Y.-Z., KAKIUCHI, N., & NAMBA, T. (1987). New acyclic bis-phenylpropanoids from the aril of Myristica fragrans. *Chemical and Pharmaceutical Bulletin*, *35*(2), 668–674. https://doi.org/10.1248/cpb.35.668

HATTORI, M., HADA, S., WATAHIKI, A., IHARA, H., SHU, Y.-Z., KAKIUCHI, N., MIZUNO, T., & NAMBA, T. (1986). Studies on dental caries prevention by traditional medicines. X Antibacterial action of phenolic components from mace against Streptococcus mutans. *Chemical and Pharmaceutical Bulletin*, *34*(9), 3885–3893. https://doi.org/10.1248/cpb.34.3885

HATTORI, M., YANG, X.-W., SHU, Y.-Z., KAKIUCHI, N., TEZUKA, Y., KIKUCHI, T., & NAMBA, T. (1988). New constituents of the aril of Myristica fragrans. *Chemical and Pharmaceutical Bulletin*, *36*(2), 648–653. https://doi.org/10.1248/cpb.36.648

Hattori, M., Yang, X., Miyashiro, H., & Namba, T. (1993). Inhibitory effects of monomeric and dimeric phenylpropanoids from mace on lipid peroxidation *In Vivo* and *In Vitro*. *Phytotherapy Research*, *7*(6), 395–401. https://doi.org/10.1002/ptr.2650070603

He, D. D., Fan, J. T., You, R. R., Chen, X. Q., Li, Z. L., Ju, J. M., & Zeng, B. L. (2017). Identification of chemical constituents in Swertia chirayita by UPLC-ESI-Q-TOF MS. *Journal of Chinese Mass Spectrometry Society*, *38*(1), 138–145. https://doi.org/10.7538/ZPXB.2017.38.01.0138

Hou, J., Wu, H., … Y. W.-C. J. of F., & 2012, undefined. (2012). Isolation of Some Compounds from Nutmeg and their Antioxidant Activities. *Agriculturejournals.CzJP Hou, H Wu, Y Wang, XC WengCzech Journal of Food Sciences, 2012•agriculturejournals.Cz*, *30*(2), 164–170.

Hwang, J. T., Kim, Y., Jang, H.-J., Oh, H.-M., Lim, C.-H., Lee, S. W., Rho, M.-C., & Schmidt, T. J. (2016). Study of the UV Light Conversion of Feruloyl Amides from Portulaca oleracea and Their Inhibitory Effect on IL-6-Induced STAT3 Activation. *Mdpi.ComJT Hwang, Y Kim, HJ Jang, HM Oh, CH Lim, SW Lee, MC RhoMolecules, 2016•mdpi.Com*. https://doi.org/10.3390/molecules21070865

Isogai, A., Murakoshi, S., Chemistry, A. S.-… and B., & 1973, undefined. (2014). Isolation from nutmeg of growth inhibitory substances to silkworm larvae. *Taylor & FrancisA Isogai, S Murakoshi, A Suzuki, S TamuraAgricultural and Biological Chemistry, 1973•Taylor & Francis*, *37*(4), 889–895. https://doi.org/10.1080/00021369.1973.10860739

Isogai, A., Murakoshi, S., Suzuki, A., & Tamura, S. (1973). Isolation from Nutmeg of Growth Inhibitory Substances to Silkworm Larvae. *Agricultural and Biological Chemistry*, *37*(4), 889–895. https://doi.org/10.1080/00021369.1973.10860739

Jaiswal, P., Kumar, P., … V. S.-A. review of, & 2009, U. (2009). Biological effects of Myristica fragrans. *Arbs.Biblioteca.Unesp.BrP Jaiswal, P Kumar, VK Singh, DK SinghAnnual Review of Biomedical Sciences, 2009•arbs.Biblioteca.Unesp.Br*. https://doi.org/10.5016/1806-8774.2009v11p21

Kang, J. W., Min, B., & Lee, J. (2013). Anti‐platelet Activity of *Erythro* ‐(7 *S* ,8 *R* )‐7‐acetoxy‐3,4,3′,5′‐tetramethoxy‐8‐O‐4′‐neolignan from *Myristica fragrans*. *Phytotherapy Research*, *27*(11), 1694–1699. https://doi.org/10.1002/ptr.4923

Kapoor, I. P. S., Singh, B., Singh, G., De Heluani, C. S., De Lampasona & Cesar, M. P., & Catalan, A. N. (2013). Chemical Composition and Antioxidant Activity of Essential Oil and Oleoresins of Nutmeg (Myristica fragrans Houtt.) Fruits. *Taylor & FrancisIPS Kapoor, B Singh, G Singh, CS De Heluani, MP De Lampasona, CAN CatalanInternational Journal of Food Properties, 2013•Taylor & Francis*, *16*(5), 1059–1070. https://doi.org/10.1080/10942912.2011.576357

Kaur, P., Gupta, R., Dey, A., Products, D. P.-I. crops and, & 2019, U. (2019). Simultaneous quantification of oleanolic acid, ursolic acid, betulinic acid and lupeol in different populations of five Swertia species by using HPTLC. *ElsevierP Kaur, RC Gupta, A Dey, DK PandeyIndustrial Crops and Products, 2019•Elsevier*.

Kimura, Y., Ito, H., Bulletin, T. H.-B. and P., & 2010, U. (2010). Effects of mace and nutmeg on human cytochrome P450 3A4 and 2C9 activity. *Jstage.Jst.Go.JpY Kimura, H Ito, T HatanoBiological and Pharmaceutical Bulletin, 2010•jstage.Jst.Go.Jp*.

Kuang, H. X., Yang, B. Y., Xia, Y. G., & Feng, W. S. (2008). Chemical constituents from the flower of Datura metel L. *Archives of Pharmacal Research*, *31*(9), 1094–1097. https://doi.org/10.1007/s12272-001-1274-6

Kuang, H., Yang, B., Tang, L., Xia, Y., & Dou, D. (2009). Baimantuoluosides A-C, three new withanolide glucosides from the flower of Datura metel L. *Helvetica Chimica Acta*, *92*(7), 1315–1323. https://doi.org/10.1002/HLCA.200800404

Kuang, H., Yang, B., Xia, Y., Molecules, Q. W.-, & 2011, U. (2011). Two new withanolide lactones from flos daturae. *Mdpi.ComHX Kuang, BY Yang, YG Xia, QH WangMolecules, 2011•mdpi.Com*.

Kumar, V., Sood, H., Rajinder, &, Chauhan, S., & Singh Chauhan, R. (2015). Detection of intermediates through high-resolution mass spectrometry for constructing biosynthetic pathways for major chemical constituents in a medicinally important. *Taylor & FrancisV Kumar, H Sood, RS ChauhanNatural Product Research, 2015•Taylor & Francis*, *29*(15), 1449–1455. https://doi.org/10.1080/14786419.2015.1004175

Kwon, H., Cho, S., Ha, T., … A. H.-B. of the K., & 2014, undefined. (2014). Lipoxygenase inhibitory effects of Dibenzylbutane Lignans from the seeds of Myristica fragrans (Nutmeg). *Researchgate.NetHS Kwon, SJ Cho, TJ Ha, A Harikishore, HS Yoon, KH Park, IS Kim, DS JangBulletin of the Korean Chemical Society, 2014•researchgate.Net*. https://doi.org/10.5012/bkcs.2014.35.10.3095

Kwon, H., Kim, M., Jeong, H., Yang, M., … K. P.-B. & medicinal, & 2008, U. (2008). Low-density lipoprotein (LDL)-antioxidant lignans from Myristica fragrans seeds. *ElsevierHS Kwon, MJ Kim, HJ Jeong, MS Yang, KH Park, TS Jeong, WS LeeBioorganic & Medicinal Chemistry Letters, 2008•Elsevier*.

L. Cai, S. Wang, T. Li, Y. J. X. (2006). *Cai: The phytochemical research of Swertia chirayita - Google Scholar*.

Lee, S. U., Ki, S. S., Shi, Y. R., Yong, K. M., & Seong, H. K. (2009). Machilin A isolated from Myristica fragrans stimulates osteoblast differentiation. *Planta Medica*, *75*(2), 152–157. https://doi.org/10.1055/S-0028-1112197

Li, F., Acta, X. Y.-H. C., & 2007, undefined. (2007). Three New Neolignans from the Aril of Myristica fragrans. *Wiley Online LibraryF Li, XW YangHelvetica Chimica Acta, 2007•Wiley Online Library*, *90*(8), 1491–1496. https://doi.org/10.1002/hlca.200790155

Li, F., & Yang, X. (2007). Three New Neolignans from the Aril of *Myristica fragrans*. *Helvetica Chimica Acta*, *90*(8), 1491–1496. https://doi.org/10.1002/hlca.200790155

Liu, Y., Guan, W., Lu, Z., Guo, R., Xia, Y., Lv, S., Fitoterapia, B. Y.-, & 2019, U. (2019). New sesquiterpenoids from the stems of Datura metel L. *Elsevier*.

Liu, Y., Jiang, H., Liu, Y., Algradi, A., Naseem, A., Fitoterapia, Y. Z.-, & 2020, U. (n.d.). New indole alkaloids from the seeds of Datura metel L. *Elsevier*.

Liu, Y., Pan, J., Sun, Y., Wang, X., Liu, Y., Fitoterapia, B. Y.-, & 2020, U. (2020). Immunosuppressive withanolides from the flower of Datura metel L. *Elsevier*.

Liu, Y., Wu, D.-D., Zhou, Y.-Q., Wu, J.-T., Qi, Z.-T., Algradi, A. M., Pan, J., Guan, W., Yang, B.-Y., & Kuang, H.-X. (2022a). A new ent-kaurane diterpenoid from the pericarps of Datura metel. *Taylor & Francis*, *24*(9), 884–890. https://doi.org/10.1080/10286020.2021.1981874

Liu, Y., Wu, D. D., Zhou, Y. Q., Wu, J. T., Qi, Z. T., Algradi, A. M., Pan, J., Guan, W., Yang, B. Y., & Kuang, H. X. (2022b). A new ent-kaurane diterpenoid from the pericarps of Datura metel. *Journal of Asian Natural Products Research*, *24*(9), 884–890. https://doi.org/10.1080/10286020.2021.1981874

M. Manickam, S. Kumar, A. Sinha-Bagchi, S. SinhaI, A. B. R. (1995). *Manickam: Withametelin hand withafastuosin c, two... - Google Scholar*.

Ma, L., Xie, C., Li, J., Lou, F., biodiversity, L. H.-C. &, & 2006, undefined. (2006). Daturametelins H, I, and J: Three New Withanolide Glycosides from Datura metel L. *Wiley Online LibraryL Ma, CM Xie, J Li, FC Lou, LH HuChemistry & Biodiversity, 2006•Wiley Online Library*, *3*(2), 180–186. https://doi.org/10.1002/cbdv.200690021

Mahendran, G., Verma, N., Singh, S., … S. P.-I. C. and, & 2022, U. (2022). Isolation and characterization of a novel xanthone from the hairy root cultures of Swertia chirayita (Roxb.) H. Karst. and its biological activity. *ElsevierG Mahendran, N Verma, S Singh, S Parveen, M Singh, S Luqman, K Shanker, L Ur RahmanIndustrial Crops and Products, 2022•Elsevier*.

Mai, N., Cuc, N., Anh, H., Nhiem, N., … B. T.-P., & 2017, U. (2017). Two new guaiane sesquiterpenes from Datura metel L. with anti-inflammatory activity. *ElsevierNT Mai, NT Cuc, HLT Anh, NX Nhiem, BH Tai, PH Yen, TH Quang, C Van Minh, KW KimPhytochemistry Letters, 2017•Elsevier*.

Mai, N., Cuc, N., Anh, H., Nhiem, N., Steroids, B. T.-, & 2017, U. (2017). Steroidal saponins from Datura metel. *Elsevier*.

Mandal, S., & Chatterjee, A. (1987). *Structure of chiratanin, a novel dimeric xanthone.*

Manickam, M., Awasthi, S., Sinha-Bagchi, A., Phytochemistry, S. S.-, & 1996, U. (1996). Withanolides from Datura tatula. *Elsevier*.

Manickam, M., Sinha-Bagchi, A., Sinha, S., Phytochemistry, M. G.-, & 1993, U. (1993). Withanolides of Datura fastuosa leaves. *Elsevier*.

Manickam, M., Srivastava, A., Phytochemistry, A. R.-, & 1998, U. (1998). Withanolides from the flowers of Datura fastuosa. *Elsevier*.

Min, B., Cuong, T., Hung, T., … B. M.-B. of the K., & 2011, U. (2011). Inhibitory Effect of Lignans from Myristica fragrans on LPS-induced NO Production in RAW264. 7 Cells. *Researchgate.NetBS Min, TD Cuong, TM Hung, BK Min, BS Shin, MH WooBulletin of the Korean Chemical Society, 2011•researchgate.Net*. https://doi.org/10.5012/bkcs.2011.32.11.4059

Mohiuddin, Y. G., Nathar, V. N., Aziz, W. N., & Gaikwad, N. B. (2018). Investigations on important secondary metabolites form aerial parts of Artemisia absinthium L. using GC-MS. *Journal of Pharmaconosy and Phytochemistry*, *7*(1), 820–827.

Morikawa, T., Hachiman, I., Matsuo, K., Nishida, E., Ninomiya, K., Hayakawa, T., Yoshie, O., Muraoka, O., & Nakayama, T. (2016a). Neolignans from the Arils of Myristica fragrans as Potent Antagonists of CC Chemokine Receptor 3. *ACS PublicationsT Morikawa, I Hachiman, K Matsuo, E Nishida, K Ninomiya, T Hayakawa, O YoshieJournal of Natural Products, 2016•ACS Publications*, *79*(8), 2005–2013. https://doi.org/10.1021/acs.jnatprod.6b00262

Morikawa, T., Hachiman, I., Matsuo, K., Nishida, E., Ninomiya, K., Hayakawa, T., Yoshie, O., Muraoka, O., & Nakayama, T. (2016b). Neolignans from the Arils of *Myristica fragrans* as Potent Antagonists of CC Chemokine Receptor 3. *Journal of Natural Products*, *79*(8), 2005–2013. https://doi.org/10.1021/acs.jnatprod.6b00262

Morikawa, T., Hachiman, I., Ninomiya, K., Hata, H., Sugawara, K., Muraoka, O., & Matsuda, H. (2018). Degranulation inhibitors from the arils of Myristica fragrans in antigen-stimulated rat basophilic leukemia cells. *Journal of Natural Medicines*, *72*(2), 464–473. https://doi.org/10.1007/s11418-017-1170-x

Muñoz Acuña, U., Carcache, P. J. B., Matthew, S., & Carcache de Blanco, E. J. (2016). New acyclic bis phenylpropanoid and neolignans, from Myristica fragrans Houtt., exhibiting PARP-1 and NF-κB inhibitory effects. *Food Chemistry*, *202*, 269–275. https://doi.org/10.1016/j.foodchem.2016.01.060

NĂNESCU, V., TĂNASIE, ȘE, BIȚĂ, A., … M. V.-, & 2023, undefined. (2023). POLYPHENOL QUANTIFICATION ON DIPSACUS LACINIATUS AND ARMORACIA RUSTICANA FROM ROMANIAN FLORA. *Farmaciajournal.Com*, *71*, 5. https://doi.org/10.31925/farmacia.2023.5.5

Nguyen, P., Le, T., Kang, H., Chae, J., … S. K.-B. & medicinal, & 2010, U. (2010). AMP-activated protein kinase (AMPK) activators from Myristica fragrans (nutmeg) and their anti-obesity effect. *ElsevierPH Nguyen, TVT Le, HW Kang, J Chae, SK Kim, KI Kwon, DB Seo, SJ Lee, WK OhBioorganic & Medicinal Chemistry Letters, 2010•Elsevier*.

Ousji, O., & Sleno, L. (2022). Structural Elucidation of Novel Stable and Reactive Metabolites of Green Tea Catechins and Alkyl Gallates by LC-MS/MS. *Antioxidants*, *11*(9). https://doi.org/10.3390/ANTIOX11091635

Pan, Y., Wang, X., & Hu, X. (2007). Cytotoxic withanolides from the flowers of Datura metel. *Journal of Natural Products*, *70*(7), 1127–1132. https://doi.org/10.1021/NP070096B

Pan, Y., Wang, X., products, X. H.-J. of natural, & 2007, undefined. (2007). Cytotoxic Withanolides from the Flowers of Datura metel. *ACS Publications*, *70*(7), 1127–1132. https://doi.org/10.1021/np070096b

Pandey, R., Mahar, R., Hasanain, M., Shukla, S. K., Sarkar, J., Rameshkumar, K. B., & Kumar, B. (2016). Rapid screening and quantitative determination of bioactive compounds from fruit extracts of Myristica species and their in vitro antiproliferative activity. *Food Chemistry*, *211*, 483–493. https://doi.org/10.1016/J.FOODCHEM.2016.05.065

Pant, N., Jain, D., & Bhakuni, R. (2002). Some chemical constituents of Swertia chirata†. *Indian Journal of Chemistry*, *41*, 1980–1986.

Patel, K., Compounds, D. P.-C. B., & 2020, U. (2021). Secoiridoid amarogentin from ’gentianaceae’with their health promotion, disease prevention and modern analytical aspects. *Ingentaconnect.Com*.

Pham, V., Jossang, A., Sévenet, T., Tetrahedron, B. B.-, & 2000, U. (2005). Cytotoxic acylphenols from Myristica maingayi. *Elsevier*.

Pichler, C., Filipič, M., Kundi, M., Rainer, B., Knasmueller, S., & Mišík, M. (2014). Assessment of genotoxicity and acute toxic effect of the imatinib mesylate in plant bioassays. *Chemosphere*, *115*(1), 54–58. https://doi.org/10.1016/j.chemosphere.2014.01.010

R. Guo, Y. Liu, J. Pan, W. Guan, B.Y. Yang, H. X. K. (n.d.). *Scholar (7)*.

Rastegari, A., Manayi, A., Rezakazemi, M., Eftekhari, M., Khanavi, M., Akbarzadeh, T., & Saeedi, M. (2022). Phytochemical analysis and anticholinesterase activity of aril of Myristica fragrans Houtt. *SpringerA Rastegari, A Manayi, M Rezakazemi, M Eftekhari, M Khanavi, T Akbarzadeh, M SaeediBMC Chemistry, 2022•Springer*, *16*(1). https://doi.org/10.1186/s13065-022-00897-9

Reyes-Escogido, M., Molecules, E. G.-M.-, & 2011, U. (2011). Chemical and pharmacological aspects of capsaicin. *Mdpi.ComMDL Reyes-Escogido, EG Gonzalez-Mondragon, E Vazquez-TzompantziMolecules, 2011•mdpi.Com*.

Romero-Cortes, T., … V. P. E.-C.-J. of, & 2019, U. (2019). Antifungal activity of vanilla juice and vanillin against Alternaria alternata. *Taylor & Francis*.

S. Sabita, J.M. Lal, S.D. Kumar, B. A. (2019). *Sapkota: Review on Swertia chirayita (roxb. ex flem.)... - Google Scholar*.

Seong Choi, H., Cho, J.-Y., Rim Jin, M., Geon Lee, Y., Kim, S.-J., Ham, K.-S., & Moon, J.-H. (2016). Phenolics, acyl galactopyranosyl glycerol, and lignan amides from Tetragonia tetragonioides (Pall.) Kuntze. *SpringerHS Choi, JY Cho, MR Jin, YG Lee, SJ Kim, KS Ham, JH MoonFood Science and Biotechnology, 2016•Springer*, *25*(5), 1275–1281. https://doi.org/10.1007/s10068-016-0201-9

Shan, B., Cai, Y., Sun, M., food, H. C.-J. of agricultural and, & 2005, undefined. (2005). Antioxidant capacity of 26 spice extracts and characterization of their phenolic constituents. *ACS PublicationsB Shan, YZ Cai, M Sun, H CorkeJournal of Agricultural and Food Chemistry, 2005•ACS Publications*, *53*(20), 7749–7759. https://doi.org/10.1021/jf051513y

Sharma, P. (1982). *Alkaloids of Swertia chirata Buch.--Ham.*

Sharma, P. (1983). *Triterpenoids of Swertia chirata.*

Shi, G., Lu, R., Yang, Y., Li, C., … A. Y.-… S. E. S., & 2004, U. (2004). 1-Hydroxy-2, 3, 4, 7-tetramethoxyxanthone from Swertia Chirayita. *Journals.Iucr.OrgGF Shi, RH Lu, YS Yang, C Li, AM Yang, LX CaiActa Crystallographica Section E: Structure Reports Online, 2004•journals.Iucr.Org*.

Shi, G., Lu, R., Yang, Y., Li, C., … A. Y.-J. of C., & 2005, undefined. (2005). Crystal structure of 1,5,8-trihydroxyl-3-methoxy xanthone from Swertia chirayita. *SpringerGF Shi, RH Lu, YS Yang, CL Li, AM Yang, LX CaiJournal of Chemical Crystallography, 2005•Springer*, *35*(2), 135–139. https://doi.org/10.1007/s10870-005-2802-7

Shi, G., Wang, G., Hungarica, X. C.-A. B., & 2013, undefined. (2013). Screening of radical-scavenging natural neuroprotective antioxidants from Swertia chirayita. *Akjournals.ComGF Shi, GY Wang, XF ChenActa Biologica Hungarica, 2013•akjournals.Com*, *64*(3), 267–278. https://doi.org/10.1556/ABiol.64.2013.3.1

SHINGU, K., FURUSAWA, Y., And, T. N.-C., & 1989, U. (1989). New Withanolides, Daturametelins C, D, E, F and G-Ac from Datura metel L.(Solanaceous Studies. XIV). *Jstage.Jst.Go.JpK SHINGU, Y FURUSAWA, T NOHARAChemical and Pharmaceutical Bulletin, 1989•jstage.Jst.Go.Jp*.

Siddiqui, S., Sultana, N., Ahmad, S., Phytochemistry, S. H.-, & 1987, undefined. (n.d.). A novel withanolide from Datura metel. *Elsevier*.

Singh, P., Ambika, undefined, Research, S. C.-N. P., & 2012, undefined. (2012). Activity-guided isolation of antioxidant xanthones from Swertia chirayita (Roxb.) H. Karsten (Gentianaceae). *Taylor & FrancisPP Singh, Ambika, SMS ChauhanNatural Product Research, 2012•Taylor & Francis*, *26*(18), 1682–1686. https://doi.org/10.1080/14786419.2011.592836

Suryawanshi, S., Mehrotra, N., Asthana, R. K., & Gupta, R. C. (2006). Liquid chromatography/tandem mass spectrometric study and analysis of xanthone and secoiridoid glycoside composition of Swertia chirata, a potent antidiabetic. *Wiley Online LibraryS Suryawanshi, N Mehrotra, RK Asthana, RC GuptaRapid Communications in Mass Spectrometry: An International, 2006•Wiley Online Library*, *20*(24), 3761–3768. https://doi.org/10.1002/rcm.2795

Tabassum, S., Mahmood, S., Hanif, J., Hina, M., & Uzair, B. (2012). An Overview of Medicinal Importance of SWERTIA CHIRAYITA. *International Journal of Applied Science and Technology* , *2*(1), 298–304.

Tan, J., Liu, Y., Cheng, Y., Sun, Y., Liu, Y., Steroids, J. H.-, & 2020, U. (2020). Daturmetesides AE, five new ergostane-type C28 sterols from the leaves of Datura metel L. *Elsevier*.

Tan, J., Liu, Y., Cheng, Y., Sun, Y., Pan, J., Fitoterapia, S. Y.-, & 2020, U. (2020). Anti-inflammatory sesquiterpenoids from the leaves of Datura metel L. *Elsevier*.

Tan, J., Liu, Y., Cheng, Y., Sun, Y., Pan, J., Guan, W., … X. L.-B., & 2020, U. (2020). New withanolides with anti-inflammatory activity from the leaves of Datura metel L. *ElsevierJ Tan, Y Liu, Y Cheng, Y Sun, J Pan, W Guan, X Li, J Huang, P Jiang, S Guo, H KuangBioorganic Chemistry, 2020•Elsevier*.

Tan, J. Y., Liu, Y., Cheng, Y. G., Sun, Y. P., Li, X. M., Guan, W., Pan, J., Yang, B. Y., & Kuang, H. X. (2021). Seven new glycosides from the leaves of Datura metel L. *Natural Product Research*, *36*(1), 295–304. https://doi.org/10.1080/14786419.2020.1779713

Tang, L. (2006). *Tang: Antipsoriatic Properties of the Active Fraction... - Google Scholar*.

Temerdashev, A., Kolychev, I., Analytical, N. K.-J. of, & 2012, undefined. (2012). Chromatographic determination of some tropane alkaloids in Datura metel. *SpringerAZ Temerdashev, IA Kolychev, NV KiselevaJournal of Analytical Chemistry, 2012•Springer*, *67*(12), 960–966. https://doi.org/10.1134/S1061934812120040

Thuong, P. T., Manh, T., Nguyen, H. •, Khoi, M., Hoang, •, My, T., Nguyen, N. •, Chinh, T., Nguyen, •, Quy, T., Tae, •, Jang, S., & Na, M. (2014). Cytotoxic and anti-tumor activities of lignans from the seeds of Vietnamese nutmeg Myristica fragrans. *SpringerPT Thuong, TM Hung, NM Khoi, HTM Nhung, NT Chinh, NT Quy, TS Jang, MK NaArchives of Pharmacal Research, 2014•Springer*, *37*(3), 399–403. https://doi.org/10.1007/s12272-013-0185-4

Trifan, A., Zengin, G., Plants, I. K.-G.-, & 2023, U. (2023). Essential Oils and Sustainability: In Vitro Bioactivity Screening of Myristica fragrans Houtt. Post-Distillation By-Products. *Mdpi.Com*.

Vermillion, K., Holguin, F. O., Berhow, M. A., Richins, R. D., Redhouse, T., O’Connell, M. A., Posakony, J., Mahajan, S. S., Kelly, S. M., & Simon, J. A. (2011). Dinoxin B, a withanolide from Datura inoxia leaves with specific cytotoxic activities. *Journal of Natural Products*, *74*(2), 267–271. https://doi.org/10.1021/NP1004714

Wang, S., Liu, Y., Li, X., Algradi, A., Jiang, H., Molecules, Y. S.-, & 2021, U. (2021). TREM2 by SPR biosensor-UPLC/MS recognition system, and investigating the mechanism of anti-neuroinflammatory activity on the lignin-amides from Datura metel …. *Mdpi.Com*.

Wang, X.-Y., He, J., Bai, H.-J., Gao, P.-Y., Gan, H., Yang, T., Zhang, W.-K., & Xu, J.-K. (2019). Daturanolide A–C, Three New Withanolides from Datura metel L. and Their Cytotoxic Activities. *Wiley Online LibraryXY Wang, J He, HJ Bai, PY Gao, H Gan, T Yang, WK Zhang, JK XuChemistry & Biodiversity, 2019•Wiley Online Library*, *16*(4). https://doi.org/10.1002/cbdv.201900004

Woo, S., Win, N., Oo, W., Ngwe, H., Ito, T., … I. A.-J. of bioscience, & 2019, U. (2019). Viral protein R inhibitors from Swertia chirata of Myanmar. *Elsevier*.

Yang, B.-Y., Xia, Y.-G., Wang, Y.-Y., Wang, Q.-H., & Kuang, H.-X. (2013). Two novel norwithasteroids with unusual six‐and seven‐membered ether rings in side chain from Flos Daturae. *Wiley Online LibraryBY Yang, YG Xia, YY Wang, QH Wang, HX KuangEvidence‐Based Complementary and Alternative Medicine, 2013•Wiley Online Library*, *2013*. https://doi.org/10.1155/2013/352019

Yang, B., Guo, R., Li, T., Liu, Y., Wang, C., Molecules, Z. S.-, & 2014, U. (2014). Five Withanolides from the Leaves of Datura metel L. and Their Inhibitory Effects on Nitric Oxide Production. *Mdpi.ComBY Yang, R Guo, T Li, Y Liu, CF Wang, ZP Shu, ZB Wang, J Zhang, YG Xia, H JiangMolecules, 2014•mdpi.Com*.

Yang, B., Guo, R., Li, T., Wu, J., Zhang, J., Liu, Y., Steroids, Q. W.-, & 2014, U. (2014). New anti-inflammatory withanolides from the leaves of Datura metel L. *Elsevier*.

Yang, B., Jiang, H., Liu, Y., … J. C.-J. of A. N., & 2020, undefined. (2020). Steroids from the seeds of Datura metel. *Taylor & FrancisBY Yang, HB Jiang, Y Liu, J Chen, HX KuangJournal of Asian Natural Products Research, 2020•Taylor & Francis*, *22*(3), 257–263. https://doi.org/10.1080/10286020.2018.1553164

Yang, B., Luo, Y., Liu, Y., Yin, X., Letters, Y. Z.-P., & 2018, U. (2018). New lignans from the roots of Datura metel L. *ElsevierBY Yang, YM Luo, Y Liu, X Yin, YY Zhou, HX KuangPhytochemistry Letters, 2018•Elsevier*.

Yang, B., Wang, Q., Xia, Y., Feng, W., & Kuang, H. (2007). Withanolide Compounds from the Flower of Datura metel L. *Wiley Online LibraryB Yang, Q Wang, Y Xia, W Feng, H KuangHelvetica Chimica Acta, 2007•Wiley Online Library*, *90*(8), 1522–1528. https://doi.org/10.1002/hlca.200790159

Yang, B., Wang, Q., Xia, Y., Feng, W., & Kuang, H. (2008). Baimantuoluolines D – F, Three New Withanolides from the Flower of Datura metel L. *Wiley Online Library*, *6*(1), 21. https://doi.org/10.1002/hlca.200890103

Yang, B., Xia, Y., Liu, Y., Li, L., Jiang, H., … L. Y.-P., & 2014, U. (2014). New antiproliferative and immunosuppressive withanolides from the seeds of Datura metel. *ElsevierBY Yang, YG Xia, Y Liu, L Li, H Jiang, L Yang, QH Wang, HX KuangPhytochemistry Letters, 2014•Elsevier*.

Yang, B., Xia, Y., Wang, Q., Dou, D., Fitoterapia, H. K.-, & 2010, U. (2010). Two new amide alkaloids from the flower of Datura metel L. *Elsevier*.

Yang, B., Xia, Y., Wang, Q., Dou, D., research, H. K.-A. of pharmacal, & 2010, undefined. (2010). Baimantuoluosides DG, four new withanolide glucosides from the flower of Datura metel L. *Springer*, *33*(8), 1143–1148. https://doi.org/10.1007/s12272-010-0802-4

Yang, B. Y. (2005). *Yang: Studies on Chemical Constituents and Pharmacologica... - Google Scholar*.

Yang, B. Y., Guo, R., Li, T., Wu, J. J., Zhang, J., Liu, Y., Wang, Q. H., & Kuang, H. X. (2014). New anti-inflammatory withanolides from the leaves of Datura metel L. *Steroids*, *87*, 26–34. https://doi.org/10.1016/J.STEROIDS.2014.05.003

Yang, B. Y., Jiang, H. B., Liu, Y., Chen, J., & Kuang, H. X. (2020). Steroids from the seeds of Datura metel. *Journal of Asian Natural Products Research*, *22*(3), 257–263. https://doi.org/10.1080/10286020.2018.1553164

Yang, B., Yang, C., Liu, Y., Zhi, H. K.-Z. Z. yao za, & 2018, U. (2018). Chemical constituents from roots of Datura metel. *Europepmc.OrgBY Yang, CL Yang, Y Liu, HX KuangZhongguo Zhong Yao Za Zhi= Zhongguo Zhongyao Zazhi= China Journal of, 2018•europepmc.Org*.

Yang, B. you, Zhou, Y. qiang, Liu, Y., Lu, Z. kun, & Kuang, H. xue. (2018). Ent-kaurane diterpenoids from the pericarps of Datura metel L. acted on the vascular endothelial cells via TRPC6 and NF-κB protein. *Medicinal Chemistry Research*, *27*(1), 115–121. https://doi.org/10.1007/S00044-017-2046-Z

Yang, S., Na, M. K., Jang, J. P., Kim, K. A., Kim, B. Y., Sung, N. J., Oh, W. K., & Ahn, J. S. (2006). Inhibition of protein tyrosine phosphatase 1B by lignans from Myristica fragrans. *Phytotherapy Research*, *20*(8), 680–682. https://doi.org/10.1002/PTR.1935

You, R., Chen, X., He, D., … C. H.-Z. Z. yao, & 2017, U. (2017). Chemical constituents from petroleum ether fraction of Swertia chirayita and their activities in vitro. *Europepmc.OrgRR You, XQ Chen, DD He, CG Huang, Y Jin, SH Qian, JM Ju, JT FanZhongguo Zhong Yao Za Zhi= Zhongguo Zhongyao Zazhi= China Journal of, 2017•europepmc.Org*.

Z.Y. Li, H.X. Kuang, Y.G. Xia, B. Y. Y. (2010). *Li: Study on chemical constituents from the Datura... - Google Scholar*.

Zanousi, M. B. P., Nekoei, M., & Mohammadhosseini, M. (2016). Composition of the Essential Oils and Volatile Fractions of Artemisia absinthium by Three Different Extraction Methods: Hydrodistillation, Solvent-Free Microwave Extraction and Headspace Solid-Phase Microextraction Combined with a Novel QSRR Evaluation. *Journal of Essential Oil-Bearing Plants*, *19*(7), 1561–1581. https://doi.org/10.1080/0972060X.2014.1001139

Zhang, C.-R., Jayashree, E., Kumar, P. S., & Nair, M. G. (2015). Antioxidant and Anti-inflammatory Compounds in Nutmeg ( *Myristica Fragrans* ) Pericarp as Determined by *in vitro* Assays. *Natural Product Communications*, *10*(8), 1934578X1501000. https://doi.org/10.1177/1934578X1501000822

Zhanga, C. R., Jayashree, E., Kumar, P. S., & Nair, M. G. (2015). Antioxidant and antiinflammatory compounds in nutmeg (myristica fragrans) pericarp as determined by in vitro assays. *Natural Product Communications*, *10*(8), 1399–1402. https://doi.org/10.1177/1934578X1501000822

Zhou, N., Geng, C., Huang, X., Ma, Y., Fitoterapia, X. Z.-, & 2015, U. (2015). Anti-hepatitis B virus active constituents from Swertia chirayita. *Elsevier*.
