# Supplementary material for: Computer-assisted discovery of natural inhibitors for platelet-derived growth factor alpha as novel therapeutics for thyroid cancer
Source: Front Pharmacol. 2025 Jan 9;15:1512864. doi: 10.3389/fphar.2024.1512864 (PMC11754405; doi:10.3389/fphar.2024.1512864)
Supplement: Supplementary file 2 [file Table2.docx]

**Table S2.** Druglikeness analysis of top 119 compounds that showed docking score higher than standard imatinib against PDGFRA. Compounds that meet the acceptable druglikeness criteria are highlighted in bold and selected for further analysis.

| **Sr.no.** | **Compound Name** | **Lipinski rule** | **Ghose rule** | **Veber rule** | **Egan rule** | **Muegge rule** | **Bio-availability score** |
| --- | --- | --- | --- | --- | --- | --- | --- |
|  | Imatinib | 0 violation | No; 2 violations: MW>480, MR>130 | Yes | Yes | Yes | 0.55 |
|  | Nonacosanyl hentriacontanoate | No; 2 violations: MW>500, MLogP>4.15 | No; 4 violations: MW>480, WLogP>5.6, MR>130, #atoms>70 | No; 1 violation: Rotors>10 | No; 1 violation: WLogP>5.88 | No; 3 violations: MW>600, XLogP3>5, Rotors>15 | 0.17 |
|  | Trimyristin | No; 2 violations: MW>500, MLogP>4.15 | No; 4 violations: MW>480, WLogP>5.6, MR>130, #atoms>70 | No; 1 violation: Rotors>10 | No; 1 violation: WLogP>5.88 | No; 3 violations: MW>600, XLogP3>5, Rotors>15 | 0.17 |
|  | Glycerine-1,3-dimyristate | No; 2 violations: MW>500, MLogP>4.15 | No; 4 violations: MW>480, WLogP>5.6, MR>130, #atoms>70 | No; 1 violation: Rotors>10 | No; 1 violation: WLogP>5.88 | No; 3 violations: MW>600, XLogP3>5, Rotors>15 | 0.17 |
|  | Melongenamide D | No; 2 violations: MW>500, MLogP>4.15 | No; 4 violations: MW>480, WLogP>5.6, MR>130, #atoms>70 | No; 1 violation: Rotors>10 | No; 1 violation: WLogP>5.88 | No; 3 violations: MW>600, XLogP3>5, Rotors>15 | 0.17 |
|  | Daturametelin G-Ac | No; 2 violations: MW>500, NorO>10 | No; 3 violations: MW>480, MR>130, #atoms>70 | No; 2 violations: Rotors>10, TPSA>140 | No; 1 violation: TPSA>131.6 | No; 3 violations: MW>600, TPSA>150, H-acc>10 | 0.17 |
|  | quercetin 3-O-α-l-rhamnopyranosyl-(1 → 6)-O-[α-l-rhamnopyranosyl-(1 → 2)]-O-β-d-galactopyranoside | No; 3 violations: MW>500, NorO>10, NHorOH>5 | No; 4 violations: MW>480, WLOGP<-0.4, MR>130, #atoms>70 | No; 1 violation: TPSA>140 | No; 1 violation: TPSA>131.6 | No; 5 violations: MW>600, XLOGP3<-2, TPSA>150, H-acc>10, H-don>5 | 0.17 |
|  | grossamide K | No; 2 violations: MW>500, NHorOH>5 | No; 3 violations: MW>480, MR>130, #atoms>70 | No; 2 violations: Rotors>10, TPSA>140 | No; 1 violation: TPSA>131.6 | No; 4 violations: MW>600, TPSA>150, Rotors>15, H-don>5 | 0.17 |
|  | Tribulusamide A | Yes; 1 violation: MW>500 | No; 3 violations: MW>480, MR>130, #atoms>70 | No; 2 violations: Rotors>10, TPSA>140 | No; 1 violation: TPSA>131.6 | No; 1 violation: MW>600 | 0.55 |
|  | Cannabisin F | Yes; 1 violation: MW>500 | No; 3 violations: MW>480, MR>130, #atoms>70 | No; 2 violations: Rotors>10, TPSA>140 | No; 1 violation: TPSA>131.6 | No; 3 violations: MW>600, XLOGP3>5, Rotors>15 | 0.55 |
|  | Quercetin 3-*O*-2-(*E*-caffeoyl)- *α*-L-arabinopyranosyl-(1 → 2)- *β*-D-glucopyranoside-7-*O*-*β*-D-glucoside | No; 3 violations: MW>500, NorO>10, NHorOH>5 | No; 4 violations: MW>480, WLOGP<-0.4, MR>130, #atoms>70 | No; 2 violations: Rotors>10, TPSA>140 | No; 1 violation: TPSA>131.6 | No; 4 violations: MW>600, TPSA>150, H-acc>10, H-don>5 | 0.17 |
|  | Meteloside D | - | - | - | - | - | - |
|  | Meteloside G | - | - | - | - | - | - |
|  | Alangisesquin A | No; 3 violations: MW>500, NorO>10, NHorOH>5 | No; 3 violations: MW>480, MR>130, #atoms>70 | No; 2 violations: Rotors>10, TPSA>140 | No; 1 violation: TPSA>131.6 | No; 5 violations: MW>600, TPSA>150, Rotors>15, H-acc>10, H-don>5 | 0.17 |
|  | *erythro*-Buddlenol B | No; 3 violations: MW>500, NorO>10, NHorOH>5 | No; 3 violations: MW>480, MR>130, #atoms>70 | No; 2 violations: Rotors>10, TPSA>140 | No; 1 violation: TPSA>131.6 | No; 5 violations: MW>600, TPSA>150, Rotors>15, H-acc>10, H-don>5 | 0.17 |
|  | Grossamide | Yes; 1 violation: MW>500 | No; 3 violations: MW>480, MR>130, #atoms>70 | No; 2 violations: Rotors>10, TPSA>140 | No; 1 violation: TPSA>131.6 | No; 1 violation: MW>600 | 0.55 |
|  | Thoreliamide C | No; 3 violations: MW>500, NorO>10, NHorOH>5 | No; 4 violations: MW>480, WLOGP>5.6, MR>130, #atoms>70 | No; 2 violations: Rotors>10, TPSA>140 | No; 2 violations: WLOGP>5.88, TPSA>131.6 | No; 6 violations: MW>600, XLOGP3>5, TPSA>150, Rotors>15, H-acc>10, H-don>5 | 0.17 |
|  | (*E*, *E*)-*N*, *N*-dityramin-4,4′ - dihydroxy-3,5′ -dimethoxy-*β*,3′ - bicin-namamide | No; 3 violations: MW>500, NorO>10, NHorOH>5 | No; 4 violations: MW>480, WLogP>5.6, MR>130, #atoms>70 | No; 2 violations: Rotors>10, TPSA>140 | No; 2 violations: WLogP>5.88, TPSA>131.6 | No; 6 violations: MW>600, XLogP3>5, TPSA>150, Rotors>15, H-acc>10, H-don>5 | 0.17 |
|  | *cis*-Cannabisin E | No; 3 violations: MW>500, NorO>10, NHorOH>5 | No; 3 violations: MW>480, MR>130, #atoms>70 | No; 2 violations: Rotors>10, TPSA>140 | No; 1 violation: TPSA>131.6 | No; 4 violations: MW>600, TPSA>150, Rotors>15, H-don>5 | 0.17 |
|  | 7-Hydroxy-1-(4-hydroxy-3- methoxyphenyl)-*N2, N3*-bis(4- hydroxyphen-ethyl)-6-methoxy- 1,2-dihydronaphthalene-2,3- dicarboxa-mide | No; 2 violations: MW>500, NHorOH>5 | No; 3 violations: MW>480, MR>130, #atoms>70 | No; 2 violations: Rotors>10, TPSA>140 | No; 1 violation: TPSA>131.6 | No; 3 violations: MW>600, TPSA>150, H-don>5 | 0.17 |
|  | METELOSIDE C |  |  |  |  |  |  |
|  | Melongenamide B | Yes; 1 violation: MW>500 | No; 3 violations: MW>480, MR>130, #atoms>70 | No; 2 violations: Rotors>10, TPSA>140 | No; 2 violations: Rotors>10, TPSA>140 | No; 2 violations: MW>600, TPSA>150 | 0.55 |
|  | *N*1, *N*5, *N*10-tri-*p*-coumaroylspermidine | Yes; 1 violation: MW>500 | No; 3 violations: MW>480, MR>130, #atoms>70 | No; 1 violation: Rotors>10 | No; 1 violation: TPSA>131.6 | No; 1 violation: Rotors>15 | 0.55 |
|  | Cannabisin E | No; 3 violations: MW>500, NorO>10, NHorOH>5 | No; 3 violations: MW>480, MR>130, #atoms>70 | No; 2 violations: Rotors>10, TPSA>140 | No; 1 violation: TPSA>131.6 | No; 4 violations: MW>600, TPSA>150, Rotors>15, H-don>5 | 0.17 |
|  | Kaempferol-3-*O*- *β*-glucopyranosyl (1 → 2)- *β*-glucopyranose-7-*O*- *α*-rhamnopyranoside | - | - | - | - | - | - |
|  | Cannabisin G | - | - | - | - | - | - |
|  | Kaurane acid glycoside A | No; 3 violations: MW>500, NorO>10, NHorOH>5 | No; 4 violations: MW>480, WLOGP<-0.4, MR>130, #atoms>70 | No; 1 violation: TPSA>140 | No; 1 violation: TPSA>131.6 | No; 4 violations: MW>600, TPSA>150, H-acc>10, H-don>5 | 0.17 |
|  | (+) (7*R*,7′ *R*,7′′ *R*,7′′′ *R*,8*S*,8′ *S*,8′′ *S*,8′′′ *S*)- 4′′ ,4′′′ -Dihydroxy-3,3′ ,3′′ ,3′′′ ,5,5′ - hexamethoxy-7,9′ ; 7′ ,9-diepoxy- 4,8′ ′ ; 4′ ,8′′′ -bisoxy-8,8′ - dineolignan-7′′ ,7′′′ ,9′′ ,9′′′ -tetraol | No; 3 violations: MW>500, NorO>10, NHorOH>5 | No; 3 violations: MW>480, MR>130, #atoms>70 | No; 2 violations: Rotors>10, TPSA>140 | No; 1 violation: TPSA>131.6 | No; 5 violations: MW>600, TPSA>150, Rotors>15, H-acc>10, H-don>5 | 0.17 |
|  | Ginsenoside Rg1 | No; 3 violations: MW>500, NorO>10, NHorOH>5 | No; 3 violations: MW>480, MR>130, #atoms>70 | No; 1 violation: TPSA>140 | No; 1 violation: TPSA>131.6 | No; 4 violations: MW>600, TPSA>150, H-acc>10, H-don>5 | 0.17 |
|  | Daturafoliside J | No; 3 violations: MW>500, NorO>10, NHorOH>5 | No; 3 violations: MW>480, MR>130, #atoms>70 | No; 1 violation: TPSA>140 | No; 1 violation: TPSA>131.6 | No; 4 violations: MW>600, TPSA>150, H-acc>10, H-don>5 | 0.17 |
|  | (-S)-Secoisolariciresinol-4-*O*-*β*-D-glucopyranoside | No; 3 violations: MW>500, NorO>10, NHorOH>5 | No; 3 violations: MW>480, MR>130, #atoms>70 | No; 2 violations: Rotors>10, TPSA>140 | No; 1 violation: TPSA>131.6 | No; 4 violations: MW>600, TPSA>150, H-acc>10, H-don>5 | 0.17 |
|  | Kaempferol-3-*O*-*β*-D-glucopyranose(l→2)-*β*-D-glucopyranoside-7-*O*-*β*-D-glucopyranoside | No; 3 violations: MW>500, NorO>10, NHorOH>5 | No; 4 violations: MW>480, WLogP<-0.4, MR>130, #atoms>70 | No; 1 violation: TPSA>140 | No; 1 violation: TPSA>131.6 | No; 5 violations: MW>600, XLogP3<-2, TPSA>150, H-acc>10, H-don>5 | 0.17 |
|  | Canabisine H | Yes; 1 violation: MW>500 | No; 2 violations: MW>480, MR>130 | No; 1 violation: Rotors>10 | No; 1 violation: TPSA>131.6 | yes | 0.55 |
|  | (7*S*,8*R*)-Dehydrodiconiferyl alcohol 9′ -*O*-*β*-glucopyranoside | No; 2 violations: NorO>10, NHorOH>5 | No; 1 violation: MW>480 | No; 1 violation: TPSA>140 | No; 1 violation: TPSA>131.6 | No; 3 violations: TPSA>150, H-acc>10, H-don>5 | 0.17 |
|  | Olean-12-ene-28-carboxy-3-*β*-hexadecanoate | No; 2 violations: MW>500, MLogP>4.15 | No; 4 violations: MW>480, WLogP>5.6, MR>130, #atoms>70 | No; 1 violation: Rotors>10 | No; 1 violation: WLogP>5.88 | No; 3 violations: MW>600, XLogP3>5, Rotors>15 | 0.85 |
|  | Daturafoliside D | No; 3 violations: MW>500, NorO>10, NHorOH>5 | No; 4 violations: MW>480, WLOGP<-0.4, MR>130, #atoms>70 | No; 1 violation: TPSA>140 | No; 1 violation: TPSA>131.6 | No; 4 violations: MW>600, TPSA>150, H-acc>10, H-don>5 | 0.17 |
|  | Daturmetelide P | Yes; 1 violation: MW>500 | No; 3 violations: MW>480, MR>130, #atoms>70 | No; 1 violation: TPSA>140 | No; 1 violation: TPSA>131.6 | No; 1 violation: MW>600 | 0.55 |
|  | Octacosane | Yes; 1 violation: MLogP>4.15 | No; 3 violations: WLogP>5.6, MR>130, #atoms>70 | No; 1 violation: Rotors>10 | No; 1 violation: WLogP>5.88 | No; 3 violations: XLogP3>5, Heteroatoms<2, Rotors>15 | 0.55 |
|  | Cannabisin D | No; 2 violations: MW>500, NHorOH>5 | No; 3 violations: MW>480, MR>130, #atoms>70 | No; 2 violations: Rotors>10, TPSA>140 | No; 1 violation: TPSA>131.6 | No; 3 violations: MW>600, TPSA>150, H-don>5 | 0.17 |
|  | Hyuganoside IIIb | No; 3 violations: MW>500, NorO>10, NHorOH>5 | No; 4 violations: MW>480, WLOGP<-0.4, MR>130, #atoms>70 | No; 2 violations: Rotors>10, TPSA>140 | No; 1 violation: TPSA>131.6 | No; 3 violations: TPSA>150, H-acc>10, H-don>5 | 0.17 |
|  | Sargentodoside D | No; 3 violations: MW>500, NorO>10, NHorOH>5 | No; 4 violations: MW>480, WLOGP<-0.4, MR>130, #atoms>70 | No; 2 violations: Rotors>10, TPSA>140 | No; 1 violation: TPSA>131.6 | No; 3 violations: TPSA>150, H-acc>10, H-don>5 | 0.17 |
|  | Glycopentoside F | No; 3 violations: MW>500, NorO>10, NHorOH>5 | No; 3 violations: MW>480, MR>130, #atoms>70 | No; 2 violations: Rotors>10, TPSA>140 | No; 1 violation: TPSA>131.6 | No; 5 violations: MW>600, TPSA>150, Rotors>15, H-acc>10, H-don>5 | 0.17 |
|  | Daucosterol | Yes; 1 violation: MW>500 | No; 4 violations: MW>480, WLogP>5.6, MR>130, #atoms>70 | Yes | Yes | No; 1 violation: XLogP3>5 | 0.55 |
|  | Darurafoliside U | - | - | - | - | - | - |
|  | Daturafoliside H | No; 2 violations: MW>500, NorO>10 | No; 3 violations: MW>480, MR>130, #atoms>70 | No; 1 violation: TPSA>140 | No; 1 violation: TPSA>131.6 | No; 3 violations: MW>600, TPSA>150, H-acc>10 | 0.17 |
|  | Swertiachiridiol A | No; 2 violations: MW>500, MLogP>4.15 | No; 4 violations: MW>480, WLogP>5.6, MR>130, #atoms>70 | No; 1 violation: Rotors>10 | No; 1 violation: WLogP>5.88 | No; 3 violations: MW>600, XLogP3>5, Rotors>15 | 0.17 |
|  | Conicaoside | No; 3 violations: MW>500, NorO>10, NHorOH>5 | No; 3 violations: MW>480, MR>130, #atoms>70 | No; 1 violation: TPSA>140 | No; 1 violation: TPSA>131.6 | No; 3 violations: TPSA>150, H-acc>10, H-don>5 | 0.17 |
|  | ***cis*-Grossamide K** | **Yes; 0 violation** | **No; 2 violations: MW>480, MR>130** | **Yes** | **Yes** | **Yes** | **0.55** |
|  | Discoroside D | - | - | - | - | - | - |
|  | Daturmetelide N | No; 2 violations: MW>500, NHorOH>5 | No; 3 violations: MW>480, MR>130, #atoms>70 | No; 1 violation: TPSA>140 | No; 1 violation: TPSA>131.6 | No; 3 violations: MW>600, TPSA>150, H-don>5 | 0.17 |
|  | (+)-Pinoresinol-*O*-*β*-D-diglucopyranoside | No; 3 violations: MW>500, NorO>10, NHorOH>5 | No; 4 violations: MW>480, WLOGP<-0.4, MR>130, #atoms>70 | No; 1 violation: TPSA>140 | No; 1 violation: TPSA>131.6 | No; 4 violations: MW>600, TPSA>150, H-acc>10, H-don>5 | 0.17 |
|  | Daturafoliside R | Yes; 1 violation: MW>500 | No; 3 violations: MW>480, MR>130, #atoms>70 | No; 1 violation: TPSA>140 | No; 1 violation: TPSA>131.6 | No; 2 violations: MW>600, TPSA>150 | 0.55 |
|  | Notoginsenosides R1 | - | - | - | - | - | - |
|  | Daturafoliside Y | No; 3 violations: MW>500, NorO>10, NHorOH>5 | No; 3 violations: MW>480, MR>130, #atoms>70 | No; 1 violation: TPSA>140 | No; 1 violation: TPSA>131.6 | No; 4 violations: MW>600, TPSA>150, H-acc>10, H-don>5 | 0.17 |
|  | (+)-Lariciresinol-9-*O*-*β*-D-glucopyranoside | No; 2 violations: MW>500, NorO>10 | No; 3 violations: MW>480, MR>130, #atoms>70 | No; 2 violations: Rotors>10, TPSA>140 | No; 1 violation: TPSA>131.6 | No; 4 violations: MW>600, TPSA>150, Rotors>15, H-acc>10 | 0.17 |
|  | 3-*β*-Hydroxy-11-oxo-olean-12-enyl-3-palmitate | No; 2 violations: MW>500, MLogP>4.15 | No; 4 violations: MW>480, WLogP>5.6, MR>130, #atoms>70 | No; 1 violation: Rotors>10 | No; 1 violation: WLogP>5.88 | No; 3 violations: MW>600, XLogP3>5, Rotors>15 | 0.17 |
|  | (22*R*)-27-Hydroxy-7*α*- methoxy-1-oxowitha-3,5,24- trienolide-24-*O*-*β*-*D*-glucoside | Yes; 1 violation: MW>500 | No; 3 violations: MW>480, MR>130, #atoms>70 | No; 1 violation: TPSA>140 | No; 1 violation: TPSA>131.6 | No; 2 violations: MW>600, TPSA>150 | 0.55 |
|  | Hepatacosane | Yes; 1 violation: MLogP>4.15 | No; 3 violations: WLogP>5.6, MR>130, #atoms>70 | No; 1 violation: Rotors>10 | No; 1 violation: WLogP>5.88 | No; 3 violations: XLogP3>5, Heteroatoms<2, Rotors>15 | 0.55 |
|  | Meteloside B | - | - | - | - | - | - |
|  | Meteloside A | No; 3 violations: MW>500, NorO>10, NHorOH>5 | No; 3 violations: MW>480, MR>130, #atoms>70 | No; 1 violation: TPSA>140 | No; 1 violation: TPSA>131.6 | No; 5 violations: MW>600, TPSA>150, #rings>7, H-acc>10, H-don>5 | 0.17 |
|  | Daturafoliside E | No; 3 violations: MW>500, NorO>10, NHorOH>5 | No; 3 violations: MW>480, MR>130, #atoms>70 | No; 1 violation: TPSA>140 | No; 1 violation: TPSA>131.6 | No; 4 violations: MW>600, TPSA>150, H-acc>10, H-don>5 | 0.17 |
|  | Quercetin 3-*O*-rutinoside-7-*O*-glucoside | No; 3 violations: MW>500, NorO>10, NHorOH>5 | No; 4 violations: MW>480, WLogP<-0.4, MR>130, #atoms>70 | No; 1 violation: TPSA>140 | No; 1 violation: TPSA>131.6 | No; 5 violations: MW>600, XLogP3<-2, TPSA>150, H-acc>10, H-don>5 | 0.17 |
|  | Daturataturin B | No; 3 violations: MW>500, NorO>10, NHorOH>5 | No; 3 violations: MW>480, MR>130, #atoms>70 | No; 1 violation: TPSA>140 | No; 1 violation: TPSA>131.6 | No; 4 violations: MW>600, TPSA>150, H-acc>10, H-don>5 | 0.17 |
|  | Dmetelin C (terpenoid) | No; 3 violations: MW>500, NorO>10, NHorOH>5 | No; 3 violations: MW>480, MR>130, #atoms>70 | No; 1 violation: TPSA>140 | No; 1 violation: TPSA>131.6 | No; 4 violations: MW>600, TPSA>150, H-acc>10, H-don>5 | 0.17 |
|  | Kaempferol-3-*O*-*α*-L-rhamnopyranose(l→6)-*β*-D-glucopyranoside-7-*O*-*β*-D-glucopyranoside | No; 3 violations: MW>500, NorO>10, NHorOH>5 | No; 4 violations: MW>480, WLOGP<-0.4, MR>130, #atoms>70 | No; 1 violation: TPSA>140 | No; 1 violation: TPSA>131.6 | No; 5 violations: MW>600, XLOGP3<-2, TPSA>150, H-acc>10, H-don>5 | 0.17 |
|  | kaurane daturoside A | No; 3 violations: MW>500, NorO>10, NHorOH>5 | No; 4 violations: MW>480, WLOGP<-0.4, MR>130, #atoms>70 | No; 1 violation: TPSA>140 | No; 1 violation: TPSA>131.6 | No; 4 violations: MW>600, TPSA>150, H-acc>10, H-don>5 | 0.17 |
|  | Methyl-(*Z*)-9-octadecenoate | No; 3 violations: MW>500, NorO>10, NHorOH>5 | No; 4 violations: MW>480, WLOGP<-0.4, MR>130, #atoms>70 | No; 1 violation: TPSA>140 | No; 1 violation: TPSA>131.6 | No; 4 violations: MW>600, TPSA>150, H-acc>10, H-don>5 | 0.17 |
|  | Cherry glycoside | Yes; 1 violation: NHorOH>5 | Yes | No; 1 violation: TPSA>140 | No; 1 violation: TPSA>131.6 | No; 2 violations: TPSA>150, H-don>5 | 0.55 |
|  | Stigmasterol-3-*O*-*β*-D-glucoside | Yes; 1 violation: MW>500 | No; 3 violations: MW>480, MR>130, #atoms>70 | yes | yes | No; 1 violation: XLOGP3>5 | 0.55 |
|  | Meteloside E | - | - | - | - | - | - |
|  | Hexacosane | Yes; 1 violation: MLogP>4.15 | No; 2 violations: WLogP>5.6, #atoms>70 | No; 1 violation: Rotors>10 | No; 1 violation: WLogP>5.88 | No; 3 violations: XLogP3>5, Heteroatoms<2, Rotors>15 | 0.55 |
|  | Kaempferol-3,7-*O*-diglucoside | No; 3 violations: MW>500, NorO>10, NHorOH>5 | No; 4 violations: MW>480, WLogP<-0.4, MR>130, #atoms>70 | No; 1 violation: TPSA>140 | No; 1 violation: TPSA>131.6 | No; 4 violations: MW>600, TPSA>150, H-acc>10, H-don>5 | 0.17 |
|  | **Daturafoliside O** | **Yes; 1 violation: MW>500** | **No; 3 violations: MW>480, MR>130, #atoms>70** | **Yes** | **Yes** | **Yes** | **0.55** |
|  | Kaempferol-3-*O*-*β*-D-glucosyl (l→2)-*β*-D-galactoside-7-*O*-*β*-D-glucoside | No; 3 violations: MW>500, NorO>10, NHorOH>5 | No; 4 violations: MW>480, WLogP<-0.4, MR>130, #atoms>70 | No; 1 violation: TPSA>140 | No; 1 violation: TPSA>131.6 | No; 5 violations: MW>600, XLogP3<-2, TPSA>150, H-acc>10, H-don>5 | 0.17 |
|  | Daturafoliside P | No; 3 violations: MW>500, NorO>10, NHorOH>5 | No; 3 violations: MW>480, MR>130, #atoms>70 | No; 1 violation: TPSA>140 | No; 1 violation: TPSA>131.6 | No; 4 violations: MW>600, TPSA>150, H-acc>10, H-don>5 | 0.17 |
|  | Giganteone A | No; 2 violations: MW>500, NHorOH>5 | No; 4 violations: MW>480, WLogP>5.6, MR>130, #atoms>70 | No; 2 violations: Rotors>10, TPSA>140 | No; 2 violations: WLogP>5.88, TPSA>131.6 | No; 5 violations: MW>600, XLogP3>5, TPSA>150, Rotors>15, H-don>5 | 0.17 |
|  | Astragaloside II | No; 3 violations: MW>500, NorO>10, NHorOH>5 | No; 3 violations: MW>480, MR>130, #atoms>70 | No; 1 violation: TPSA>140 | No; 1 violation: TPSA>131.6 | No; 5 violations: MW>600, TPSA>150, #rings>7, H-acc>10, H-don>5 | 0.17 |
|  | ***N*-*cis*-feruloyltyramine** | **Yes; 0 violation** | **Yes** | **Yes** | **Yes** | **Yes** | **0.55** |
|  | cannabisin A | No; 2 violations: MW>500, NHorOH>5 | No; 3 violations: MW>480, MR>130, #atoms>70 | No; 2 violations: Rotors>10, TPSA>140 | No; 1 violation: TPSA>131.6 | No; 3 violations: XLogP3>5, TPSA>150, H-don>5 | 0.17 |
|  | Herpetol C | No; 3 violations: MW>500, NorO>10, NHorOH>5 | No; 3 violations: MW>480, MR>130, #atoms>70 | No; 1 violation: TPSA>140 | No; 1 violation: TPSA>131.6 | No; 3 violations: TPSA>150, H-acc>10, H-don>5 | 0.17 |
|  | (7*S*,8*R*,7′ *S*,8′ *S*)-4,9,4′ ,7′ - Tetrahydroxy-3,3′ -dimethoxy-7,9′ - epoxylignan-4-*O*-*β*-D-glucopyranoside | No; 3 violations: MW>500, NorO>10, NHorOH>5 | No; 4 violations: MW>480, WLogP<-0.4, MR>130, #atoms>70 | No; 1 violation: TPSA>140 | No; 1 violation: TPSA>131.6 | No; 3 violations: TPSA>150, H-acc>10, H-don>5 | 0.17 |
|  | Iariciresinol-4’-O-β-D-glucopyranoside | No; 3 violations: MW>500, NorO>10, NHorOH>5 | No; 2 violations: MW>480, #atoms>70 | No; 1 violation: TPSA>140 | No; 1 violation: TPSA>131.6 | No; 3 violations: TPSA>150, H-acc>10, H-don>5 | 0.17 |
|  | Chenoalbicin | Yes; 1 violation: MW>500 | No; 3 violations: MW>480, MR>130, #atoms>70 | yes | yes | No; 2 violations: MW>600, XLogP3>5 | 0.55 |
|  | Erythrodiol-3-*O*-palmitate | No; 2 violations: MW>500, MLogP>4.15 | No; 4 violations: MW>480, WLogP>5.6, MR>130, #atoms>70 | No; 1 violation: Rotors>10 | No; 1 violation: WLogP>5.88 | No; 3 violations: MW>600, XLogP3>5, Rotors>15 | 0.17 |
|  | **(−)-1-(2,6-Dihydroxyphenyl)-9-[4-hydroxy-3-(p-menth-1-en-8- oxy)-phenyl]-1-nonanone** | **Yes; 0 violation** | **No; 4 violations: MW>480, WLogP>5.6, MR>130, #atoms>70** | **No; 1 violation: Rotors>10** | **No; 1 violation: WLogP>5.88** | **No; 1 violation: XLogP3>5** | **0.55** |
|  | Cannabisin L | No; 2 violations: MW>500, NorO>10 | No; 3 violations: MW>480, MR>130, #atoms>70 | No; 2 violations: Rotors>10, TPSA>140 | No; 1 violation: TPSA>131.6 | No; 2 violations: MW>600, TPSA>150 | 0.17 |
|  | Quercetin-3-*O*-rutinoside | No; 3 violations: MW>500, NorO>10, NHorOH>5 | No; 4 violations: MW>480, WLOGP<-0.4, MR>130, #atoms>70 | No; 1 violation: TPSA>140 | No; 1 violation: TPSA>131.6 | No; 4 violations: MW>600, TPSA>150, H-acc>10, H-don>5 | 0.17 |
|  | Grossamide K | No; 2 violations: MW>500, NHorOH>5 | No; 3 violations: MW>480, MR>130, #atoms>70 | No; 2 violations: Rotors>10, TPSA>140 | No; 1 violation: TPSA>131.6 | No; 4 violations: MW>600, TPSA>150, Rotors>15, H-don>5 | 0.17 |
|  | Baimantuoluoside B | No; 2 violations: MW>500, NHorOH>5 | No; 3 violations: MW>480, MR>130, #atoms>70 | No; 2 violations: Rotors>10, TPSA>140 | No; 1 violation: TPSA>131.6 | No; 4 violations: MW>600, TPSA>150, Rotors>15, H-don>5 | 0.17 |
|  | Baimantuoluoside H | Yes; 1 violation: MW>500 | No; 3 violations: MW>480, MR>130, #atoms>70 | No; 1 violation: TPSA>140 | No; 1 violation: TPSA>131.6 | Yes | 0.55 |
|  | **(1*S,*2*R*)-2-(4-Allyl-2,6-dimethoxyphenoxy)-1-(3,4-**  **dimethoxyphenyl) propan-1-ol acetate** | **Yes; 0 violation** | **Yes** | **No; 1 violation: rotors>10** | **Yes** | **Yes** | **0.55** |
|  | Amaroswerin | No; 3 violations: MW>500, NorO>10, NHorOH>5 | No; 3 violations: MW>480, MR>130, #atoms>70 | No; 1 violation: TPSA>140 | No; 1 violation: TPSA>131.6 | No; 3 violations: TPSA>150, H-acc>10, H-don>5 | 0.11 |
|  | Officinalioside | No; 3 violations: MW>500, NorO>10, NHorOH>5 | No; 4 violations: MW>480, WLogP<-0.4, MR>130, #atoms>70 | No; 2 violations: Rotors>10, TPSA>140 | No; 1 violation: TPSA>131.6 | No; 3 violations: TPSA>150, H-acc>10, H-don>5 | 0.17 |
|  | Daturafoliside X | No; 3 violations: MW>500, NorO>10, NHorOH>5 | No; 3 violations: MW>480, MR>130, #atoms>70 | No; 1 violation: TPSA>140 | No; 1 violation: TPSA>131.6 | No; 4 violations: MW>600, TPSA>150, H-acc>10, H-don>5 | 0.17 |
|  | Daturamalakoside A | Yes; 1 violation: MW>500 | No; 3 violations: MW>480, MR>130, #atoms>70 | No; 1 violation: TPSA>140 | No; 1 violation: TPSA>131.6 | No; 2 violations: MW>600, TPSA>150 | 0.55 |
|  | Daturametelin I | Yes; 1 violation: MW>500 | No; 3 violations: MW>480, MR>130, #atoms>70 | No; 1 violation: TPSA>140 | No; 1 violation: TPSA>131.6 | No; 2 violations: MW>600, TPSA>150 | 0.55 |
|  | 6’-*O*-*β*-D-glucopyranosyl sweroside | No; 3 violations: MW>500, NorO>10, NHorOH>5 | No; 2 violations: MW>480, WLOGP<-0.4 | No; 1 violation: TPSA>140 | No; 1 violation: TPSA>131.6 | No; 4 violations: XLogP3<-2, TPSA>150, H-acc>10, H-don>5 | 0.11 |
|  | Leptolepisol D | No; 2 violations: MW>500, NHorOH>5 | No; 2 violations: MW>480, MR>130 | No; 2 violations: Rotors>10, TPSA>140 | No; 1 violation: TPSA>131.6 | No; 2 violations: TPSA>150, H-don>5 | 0.17 |
|  | Baimantuoluoline G | No; 2 violations: MW>500, NorO>10 | No; 3 violations: MW>480, MR>130, #atoms>70 | No; 1 violation: TPSA>140 | No; 1 violation: TPSA>131.6 | No; 3 violations: MW>600, TPSA>150, H-acc>10 | 0.17 |
|  | Kaempferol 3-*O*-rutinoside-7-*O*-glucoside | No; 3 violations: MW>500, NorO>10, NHorOH>5 | No; 4 violations: MW>480, WLOGP<-0.4, MR>130, #atoms>70 | No; 1 violation: TPSA>140 | No; 1 violation: TPSA>131.6 | No; 4 violations: MW>600, TPSA>150, H-acc>10, H-don>5 | 0.17 |
|  | Icariside E5 | No; 3 violations: MW>500, NorO>10, NHorOH>5 | No; 3 violations: MW>480, MR>130, #atoms>70 | No; 2 violations: Rotors>10, TPSA>140 | No; 1 violation: TPSA>131.6 | No; 3 violations: TPSA>150, H-acc>10, H-don>5 | 0.17 |
|  | Daturmetelide R | Yes; 1 violation: MW>500 | No; 3 violations: MW>480, MR>130, #atoms>70 | No; 1 violation: TPSA>140 | No; 1 violation: TPSA>131.6 | No; 1 violation: MW>600 | 0.55 |
|  | Dehydrodiconiferyl alcohol 4-*O*- *β*-D-glucopyranoside | No; 3 violations: MW>500, NorO>10, NHorOH>5 | No; 1 violation: MW>480 | No; 1 violation: TPSA>140 | No; 1 violation: TPSA>131.6 | No; 3 violations: TPSA>150, H-acc>10, H-don>5 | 0.17 |
|  | **Maceneolignan H** | **Yes; 0 violation** | **Yes** | **No; 1 violation: Rotors>10** | **Yes** | **Yes** | **0.55** |
|  | Astrojanoside A | - | - | - | - | - | - |
|  | Lariciresinol-4′ -*O*-*β*-D-glacoside | No; 3 violations: MW>500, NorO>10, NHorOH>5 | No; 2 violations: MW>480, #atoms>70 | No; 1 violation: TPSA>140 | No; 1 violation: TPSA>131.6 | No; 3 violations: TPSA>150, H-acc>10, H-don>5 | 0.17 |
|  | Meteloside F | Yes; 1 violation: MW>500 | No; 3 violations: MW>480, MR>130, #atoms>70 | No; 1 violation: TPSA>140 | No; 1 violation: TPSA>131.6 | No; 1 violation: MW>600 | 0.55 |
|  | 7*R*,8*R*-*threo*-4,7,9-Trihydroxy-3,3′ - dimethoxy-8-*O*-4′ -neolignan-9′ -*O*- *β*-*D*-glucopyranoside | No; 3 violations: MW>500, NorO>10, NHorOH>5 | No; 4 violations: MW>480, WLogP<-0.4, MR>130, #atoms>70 | No; 2 violations: Rotors>10, TPSA>140 | No; 1 violation: TPSA>131.6 | No; 3 violations: TPSA>150, H-acc>10, H-don>5 | 0.17 |
|  | Kaempferol-3-*O*-*β*-D-glucopyranose(l→2)-*β*-D-glucopyranoside-7-*O*-*α*-L-rhamnopyranoside | No; 3 violations: MW>500, NorO>10, NHorOH>5 | No; 4 violations: MW>480, WLogP<-0.4, MR>130, #atoms>70 | No; 1 violation: TPSA>140 | No; 1 violation: TPSA>131.6 | No; 5 violations: MW>600, XLogP3<-2, TPSA>150, H-acc>10, H-don>5 | 0.17 |
|  | Daturmetelide Q | Yes; 1 violation: MW>500 | No; 3 violations: MW>480, MR>130, #atoms>70 | No; 1 violation: TPSA>140 | No; 1 violation: TPSA>131.6 | No; 1 violation: MW>600 | 0.55 |
|  | **Erythro-2-(4-allyl-2,6-dimethoxyphenoxy)-1-(3,4,5-trimethoxyphenyl) propan-1,3-dio*l*** | **Yes; 0 violation** | **Yes** | **No; 1 violation: Rotors>10** | **Yes** | **Yes** | **0.55** |
|  | 3-*O*-Demethyl swertipunicoside | No; 3 violations: MW>500, NorO>10, NHorOH>5 | No; 3 violations: MW>480, MR>130, #atoms>70 | No; 1 violation: TPSA>140 | No; 1 violation: TPSA>131.6 | No; 4 violations: MW>600, TPSA>150, H-acc>10, H-don>5 | 0.17 |
|  | (+)-Pinoresinol 4′′ -*O*-*β*-D-glucopyranoside | No; 2 violations: MW>500, NorO>10 | No; 1 violation: MW>480 | No; 1 violation: TPSA>140 | No; 1 violation: TPSA>131.6 | No; 2 violations: TPSA>150, H-acc>10 | 0.17 |
|  | Daturafoliside F | No; 3 violations: MW>500, NorO>10, NHorOH>5 | No; 3 violations: MW>480, MR>130, #atoms>70 | No; 1 violation: TPSA>140 | No; 1 violation: TPSA>131.6 | No; 4 violations: MW>600, TPSA>150, H-acc>10, H-don>5 | 0.17 |
|  | **Myrifralignan C** | **Yes; 0 violation** | **Yes** | **Yes** | **Yes** | **Yes** | **0.55** |
|  | 8-*O*-[*β*-D-xylopyranosyl-(1→6)-*β*-D-glucopyranosyl]-1-hydroxyl- 3,7-dimethoxy xanthone | No; 3 violations: MW>500, NorO>10, NHorOH>5 | No; 4 violations: MW>480, WLogP<-0.4, MR>130, #atoms>70 | No; 1 violation: TPSA>140 | No; 1 violation: TPSA>131.6 | No; 3 violations: TPSA>150, H-acc>10, H-don>5 | 0.17 |
|  | (+)-Cycloolivil-4’-*O*-*β*-D-glucopyranoside | No; 3 violations: MW>500, NorO>10, NHorOH>5 | No; 4 violations: MW>480, WLogP<-0.4, MR>130, #atoms>70 | No; 1 violation: TPSA>140 | No; 1 violation: TPSA>131.6 | No; 1 violation: TPSA>131.6 | 0.17 |
|  | Kaempferol-3-*O*-*β*-D-glucopyranose (1 → 2)-*β*-D-glucopyranoside | No; 3 violations: MW>500, NorO>10, NHorOH>5 | No; 4 violations: MW>480, WLogP<-0.4, MR>130, #atoms>70 | No; 1 violation: TPSA>140 | No; 1 violation: TPSA>131.6 | No; 4 violations: XLogP3<-2, TPSA>150, H-acc>10, H-don>5 | 0.17 |
|  | Iariciresinol-9-*O*-*β*-D-glucopyranoside | No; 2 violations: MW>500, NorO>10 | No; 1 violation: MW>480 | No; 1 violation: TPSA>140 | No; 1 violation: TPSA>131.6 | No; 2 violations: TPSA>150, H-acc>10 | 0.17 |
|  | **stigmasteryl-3-O-β-glucoside** | **Yes; 1 violation: MW>500** | **No; 4 violations: MW>480, WLogP>5.6, MR>130, #atoms>70** | **Yes** | **Yes** | **No; 1 violation: XLogP3>5** | **0.55** |
