# Supplementary material for: Computer-assisted discovery of natural inhibitors for platelet-derived growth factor alpha as novel therapeutics for thyroid cancer
Source: Front Pharmacol. 2025 Jan 9;15:1512864. doi: 10.3389/fphar.2024.1512864 (PMC11754405; doi:10.3389/fphar.2024.1512864)
Supplement: Supplementary file 3 [file DataSheet1.pdf]

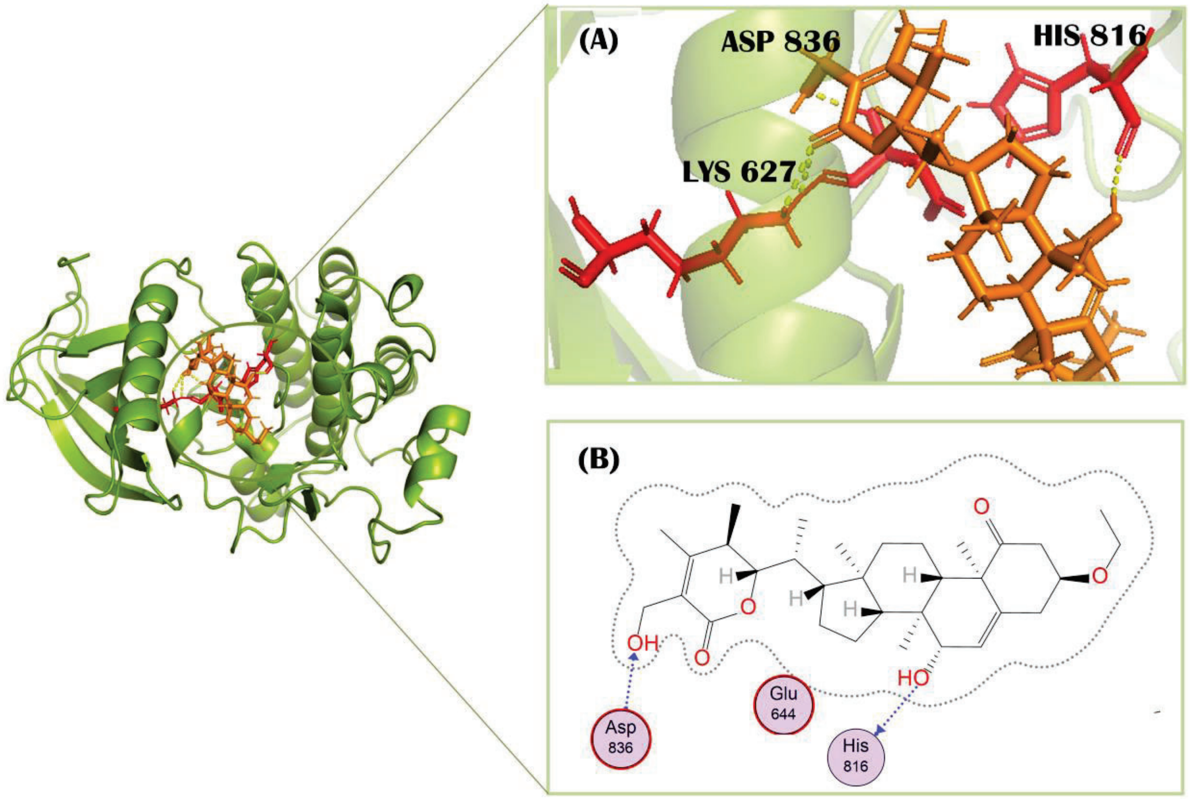

Figure S1. Molecular interactions of Protein PDGFRA (green) with Daturafoliside O (orange) (compound 2). A) 3D image of interacting residues (red) of PDGFRA; (B) 2D interaction profile of Daturafoliside O.

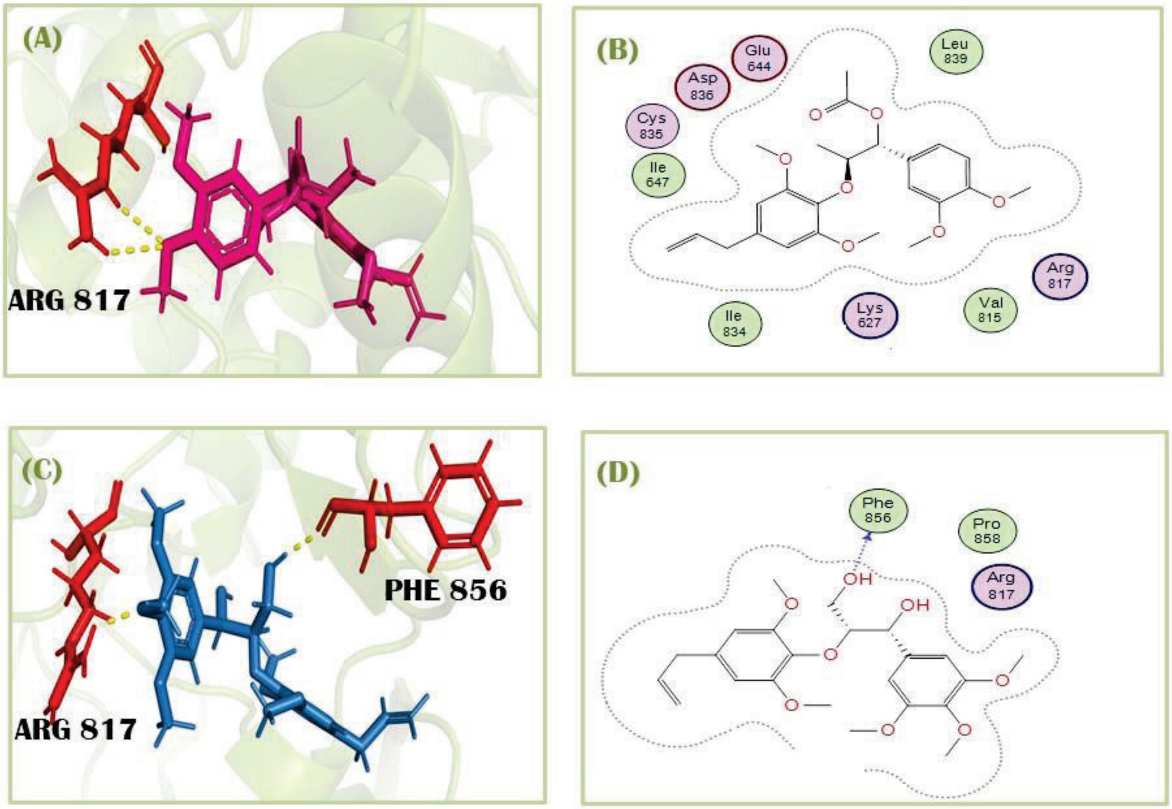

Figure S2. (A) 3D interaction of maceneolignin C (pink) (compound 4) with PDGFRA. (B) 2D interaction profile of Maceneolignin C. (C) 3D interaction of Erythro-2-(4-allyl-2,6-dimethoxyphenoxy)-1-(3,4,5-trimethoxyphenyl) propan-1,3-diol (dark blue) (compound 5) with PDGFRA (D) 2D interaction profile of Erythro-2-(4-allyl-2,6-dimethoxyphenoxy)-1-(3,4,5-trimethoxyphenyl) propan-1,3-diol.

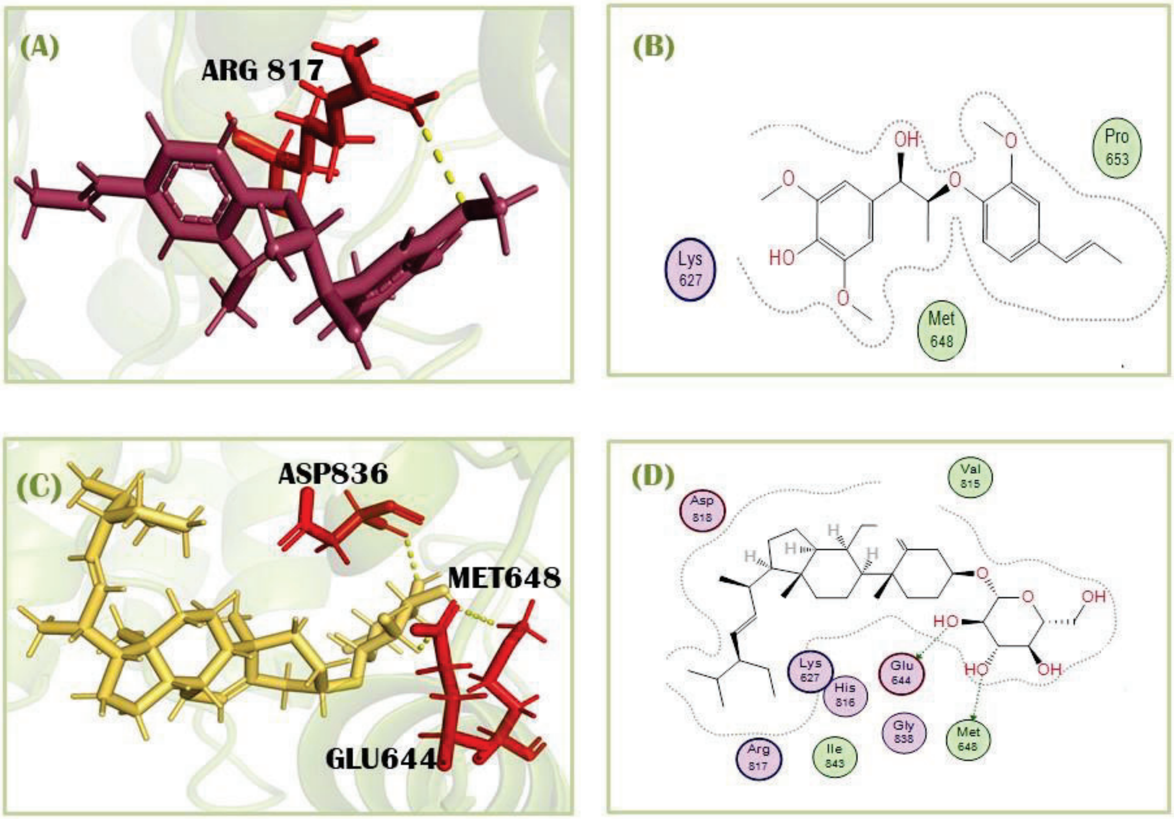

Figure S3. (A) 3D interaction of Myrifralignan C (dark selmon) (compound 6) with PDGFRA. (B) 2D interaction profile of Myrifralignan C. (C) 3D interaction of stigmasteryl-3-O-β-glucoside (yellow orange) (compound 7) with PDGFRA (D) 2D interaction profile of stigmasteryl-3-O-β-glucoside.

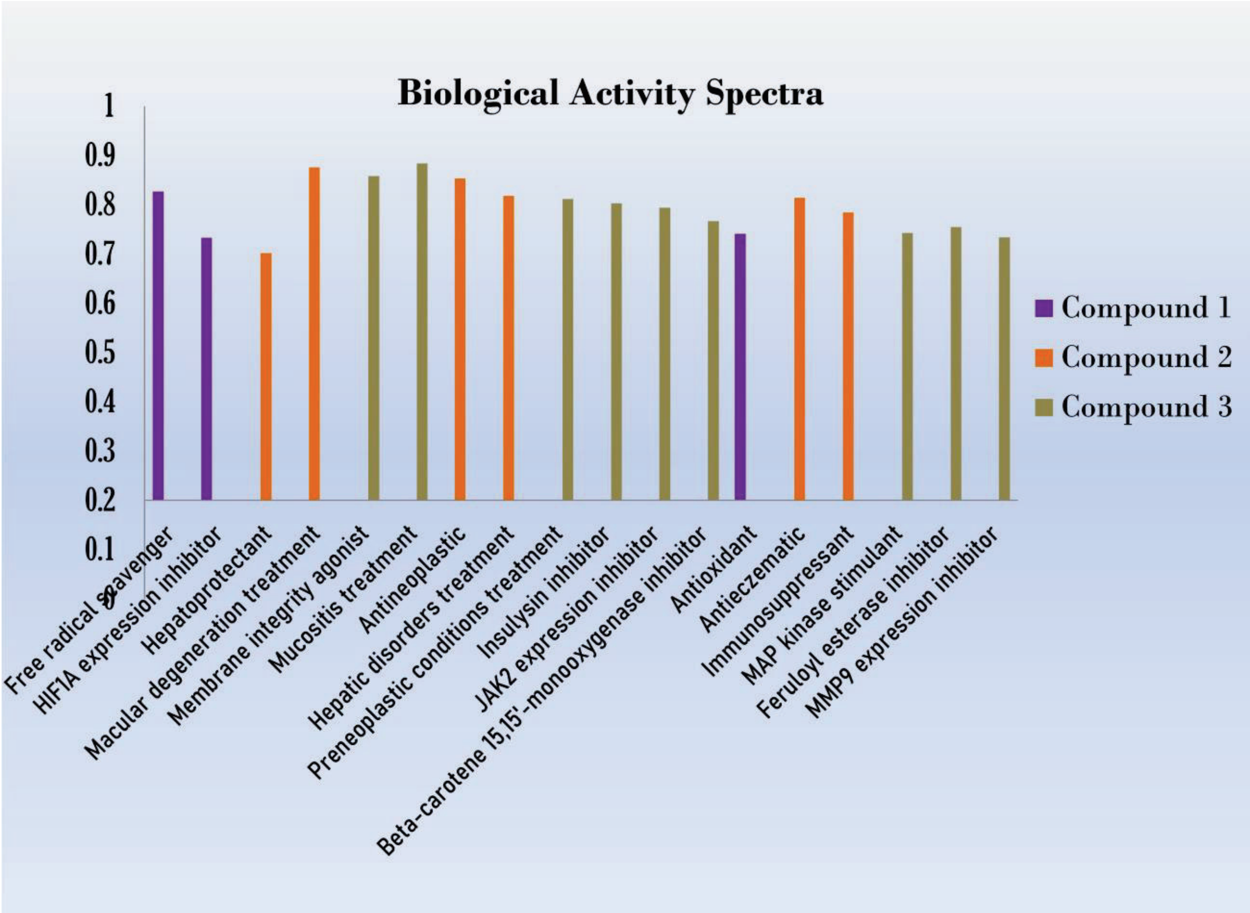

Figure S4. PASS activity predictions of the top 3 compounds, namely cis-Grossamide K, Daturafoliside O, and N-cis-feruloyltyramine.
