# Supplementary material for: Computer-assisted discovery of natural inhibitors for platelet-derived growth factor alpha as novel therapeutics for thyroid cancer
Source: Front Pharmacol. 2025 Jan 9;15:1512864. doi: 10.3389/fphar.2024.1512864 (PMC11754405; doi:10.3389/fphar.2024.1512864)
Supplement: Supplementary file 4 [file Table3.docx]

**Table S3.**  Medicinal chemistry analysis performed using the SwissADME server.

| **Sr. No.** | **Compound Name** | **Pains** | **Brenk** | **Lead-Likeness** | **Synthetic Accessibility** |
| --- | --- | --- | --- | --- | --- |
|  | cis-Grossamide K | 0 alert | 1 alert: polyene | No; 3 violations: MW>350, Rotors>7, XLOGP3>3.5 | 4.92 |
|  | Daturafoliside O | 0 alert | 1 alert: isolated_alkene | No; 2 violations: MW>350, XLOGP3>3.5 | 7.04 |
|  | N-cis-feruloyltyramine | 0 alert | 1 alert: michael_acceptor_1 | Yes | 2.55 |
|  | Maceneolignan H | 0 alert | 1 alert: isolated_alkene | No; 3 violations: MW>350, Rotors>7, XLOGP3>3.5 | 4.38 |
|  | Erythro-2-(4-allyl-2,6-dimethoxyphenoxy)-1-(3,4,5-trimethoxyphenyl) propan-1,3-diol | 0 alert | 1 alert: isolated_alkene | No; 2 violations: MW>350, Rotors>7 | 4.26 |
|  | Myrifralignan C | 0 alert | 0 alert | No; 3 violations: MW>350, Rotors>7, XLOGP3>3.5 | 4.01 |
|  | stigmasteryl-3-O-β-glucoside | 0 alert | 1 alert: isolated_alkene | No; 3 violations: MW>350, Rotors>7, XLOGP3>3.5 | 7.93 |
